# Differential Expression Profiling of Microspores During the Early Stages of Isolated Microspore Culture Using the Responsive Barley Cultivar Gobernadora

**DOI:** 10.1534/g3.118.200208

**Published:** 2018-03-12

**Authors:** Sébastien Bélanger, Suzanne Marchand, Pierre-Étienne Jacques, Blake Meyers, François Belzile

**Affiliations:** * Département de Phytologie and Institut de Biologie Intégrative et des Systèmes, Université Laval, Quebec City, Quebec, Canada, G1V 0A6; † Département de Biologie, Université de Sherbrooke, Quebec, Canada, J1K 2R1; ‡ Donald Danforth Plant Science Center, St. Louis, MO 63132; § Division of Plant Sciences, University of Missouri, Columbia, MO 65211

**Keywords:** barley, transcriptome analysis, isolated microspore culture

## Abstract

In barley, it is possible to induce embryogenesis in the haploid and uninucleate microspore to obtain a diploid plant that is perfectly homozygous. To change developmental fates in this fashion, microspores need to engage in cellular de-differentiation, interrupting the pollen formation, and restore totipotency prior to engaging in embryogenesis. In this work, we used the barley cultivar Gobernadora to characterize the transcriptome of microspores prior to (day 0) and immediately after (days 2 and 5) the application of a stress pretreatment. A deep RNA-seq analysis revealed that microspores at these three time points exhibit a transcriptome of ∼14k genes, ∼90% of which were shared. An expression analysis identified a total of 3,382 differentially expressed genes (DEGs); of these, 2,155 and 2,281 DEGs were respectively identified when contrasting expression at days 0 and 2 and at days 2 and 5. These define 8 expression profiles in which DEGs share a common up- or down-regulation at these time points. Up-regulation of numerous glutathione S-transferase and heat shock protein genes as well as down-regulation of ribosomal subunit protein genes was observed between days 0 and 2. The transition from microspores to developing embryos (days 2 *vs.* 5) was marked by the induction of transcription factor genes known to play important roles in early embryogenesis, numerous genes involved in hormone biosynthesis and plant hormonal signal transduction in addition to genes involved in secondary metabolism. This work sheds light on transcriptional changes accompanying an important developmental shift and provides candidate biomarkers for embryogenesis in barley.

Pollen formation can be divided into two developmental processes named sporogenesis and gametogenesis. Sporogenesis corresponds to the production of spores and occurs when a diploid mother cell undergoes meiosis to produce four haploid cells called microspores. These then typically undergo gametogenesis, *i.e.*, mitotic divisions and maturation resulting in a mature pollen grain composed of three nuclei (two sperm and one vegetative nucleus). The microspore, with its single nucleus, haploid set of chromosomes, functional chloroplasts and a formidable cellular plasticity, constitutes prize material for studying developmental shifts. Indeed, via anther or microspore culture, it is possible to change the developmental fate of a microspore in such a way that it engages in an embryogenic path leading to a new plant. It is possible to produce diploid plants that are perfectly homozygous (doubled haploid or DH) thanks to a spontaneous or induced doubling of the set of chromosomes present in the microspore during the development of these new plants (Germanà 2011a). Protocols for DH production have been developed and applied to various species such as rapeseed (*Brassica napus*), pepper (*Capsicum annuum* L.), tobacco (*Nicotiana tabacum* L.), wheat (*Triticum aestivum* L.), barley (*Hordeum vulgare* L.), rice (*Oryza sativa* L.) and several other species (Thomas *et al.* 2003; Dunwell 2010; Germanà 2011a). It has been reported that barley (Jacquard *et al.* 2003), wheat (Tuvesson *et al.* 2007), rapeseed (Custers 2003) and tobacco are model species due to their high response to embryogenic induction and their regeneration efficiency (Forster *et al.* 2007; Germanà 2011a,b; Soriano *et al.* 2013).

To change developmental fates, microspores need to engage in cellular de-differentiation, interrupting the transcriptional and translational activities leading to pollen formation (Maraschin *et al.* 2005), and restore totipotency (Elhiti *et al.* 2013) in view of engaging in a new developmental path, in this case embryogenesis (Hosp *et al.* 2007; Seifert *et al.* 2016). A few previous studies have characterized microspore gene expression to shed light on the mechanisms underlying microspore development in this context in barley (Vrinten *et al.* 1999; Maraschin *et al.* 2006; Muñoz-Amatriaín *et al.* 2006), wheat (Sánchez-Díaz *et al.* 2013; Seifert *et al.* 2016) and rapeseed (Joosen *et al.* 2007; Malik *et al.* 2007). In barley, experiments were performed either on isolated microspores (Vrinten *et al.* 1999; Maraschin *et al.* 2006) or whole anthers (Muñoz-Amatriaín *et al.* 2006). In wheat and rapeseed, gene expression profiling was performed on freshly and pretreated microspores in addition to embryogenic induced microspores (Joosen *et al.* 2007; Malik *et al.* 2007; Seifert *et al.* 2016). With the exception of Seifert *et al.* (2016) who used a comprehensive RNA-seq approach, all previous work was performed on candidate genes or using arrays that interrogated only a subset of all genes (from as few as ∼10 candidate genes to 21k). Nonetheless, these studies provided interesting glimpses into the main genes and metabolic processes involved in this striking change of developmental fate.

In barley, Maraschin *et al.* (2006) used a specially designed gene chip containing 1,421 ESTs isolated from the early stages of barley zygotic embryogenesis. This tool was used to analyze either freshly isolated or pretreated uninucleate microspores (following four days of heat and osmotic stress). Globally, a total of 96 differentially-expressed ESTs were identified; these coded for genes involved in the activation of protein degradation, starch and sugar hydrolysis, stress responses and cell signaling metabolisms as well as in the inhibition of programmed cell death (Maraschin *et al.* 2006). As for the work of Muñoz-Amatriaín *et al.* (2006), because it was carried out on whole anthers, it is impossible to know which transcripts were expressed in the microspores inside the anthers *vs.* the rest of the anther. Among closely related cereals, in wheat, Seifert *et al.* (2016) characterized the transcriptome of freshly isolated microspores, microspores after a pretreatment (4° for 10 days) and microspores in culture (for ∼8 days). These authors identified genes encoding transcription factors known to mark the induction of embryogenesis such as *AINTEGUMENTA-like 5* (*AIL5*) and *BABY BOOM* (*BBM*). A differential gene expression analysis identified a group of up-regulated genes involved in various epigenetic metabolisms such as DNA methylation, histone methylation and histone deacetylation.

Although barley was among the first species studied for gene expression of pretreated microspores and anthers, as described above, no comprehensive study has explored the entire transcriptome of isolated barley microspores engaging in induced embryogenesis. Consequently numerous aspects still remain obscure such as the synthetic and response pathways for various plant growth regulators as well as key transcription factors. In this work, our objective was to extensively characterize the transcriptome of barley microspores prior to (day 0) and immediately after (days 2 and 5) the application of a 48h pretreatment (thermal and osmotic stress) that efficiently induces embryogenesis in the highly responsive barley cultivar Gobernadora. To investigate key metabolic pathways involved in microspores at these stages of development, deep transcriptome sequencing was used to both catalog the genes expressed as well as those that were differentially expressed.

## MATERIAL & METHODS

### Plant materials

Donor plants of barley (H. vulgare ssp. vulgare cv Gobernadora, a two-row spring barley) were grown in a greenhouse and uniform immature spikes containing microspores at the mid-late to late-uninucleate stage were harvested as described by Esteves and Belzile (2014). We then isolated and purified microspores at three time points: Day 0 (freshly harvested spikes), Day 2 (immediately after completion of the pretreatment) and Day 5 (after three days in culture). Day 0 microspores were isolated from freshly harvested spikes containing haploid and uninucleate microspores and the uniformity of microspores was improved using gradient centrifugation (20% maltose-mannitol; 900x*g* at 12°). For day 2 and 5, the spikes were subjected to a 48-h pretreatment combining thermal (26°) and osmotic (0.3M; pH at 5.34) stresses. After pretreatment, microspores were harvested and purified using gradient centrifugation (as above). A ∼0.5M of these isolated microspores were collected as samples of day 2 while the remaining microspores were plated on a two-layer (solid-liquid) embryogenesis induction media developed by Li and Devaux (2003) and optimized by Esteves *et al.* (2014). The optimization consisted of: (i) use of 0.3 mg/l of Thidiazuron (Sigma-Aldrich, Oakville, Ontario, Canada) rather than BAP (at 1.0 mg/l), (ii) addition of 0.6 mg/l of Dicamba (Sigma-Aldrich, Oakville, Ontario, Canada), (iii) addition of 50 mg/l each of arabinogalactan (Sigma-Aldrich, Oakville, Ontario, Canada) and arabinogalactan proteins (Sigma-Aldrich, Oakville, Ontario, Canada) and (iv) removal of ovaries. Finally, to maximize the uniformity of the microspores harvested at day 5, we collected the liquid phase containing the microspores and enriched for embryogenic microspores using a 25% maltose-mannitol gradient centrifugation (300x*g*; 12°). Microspores were produced in four biological replicates and, after isolation, samples were immediately frozen in liquid nitrogen and kept at -80° prior to RNA isolation.

### Cellular fixation and microscopy of microspores

Freshly isolated microspores at the three time points were collected, fixed and DAPI stained for microscopy exactly as described in González-Melendi *et al.* (2005) except for the washing step that was performed for 15 min twice. Microscopy was performed at the Plate-forme d’Imagerie Moléculaire et de Microscopie of the Institut de Biologie Intégrative et des Systèmes (Université Laval, Québec, Canada) using 10 µl of stained microspores and observed in a Zeiss Axio Observer.Z1 (Zeiss, Gottingen, Germany) under a UV laser (excitation of 390/22 nm and emission of 460/50 nm).

### RNA isolation, library construction and sequencing

Large molecular weight RNA was differentially isolated from the small molecular weight RNA fraction using the SPLIT RNA extraction kit (Lexogen, Vienna, Austria) as per the manufacturer’s instructions. RNA quality was evaluated using the Agilent RNA 6000 Nano Kit on a Bioanalyser 2100 (Agilent Technologies, Santa Clara, CA, USA). Only RNA samples with an integrity number ≥7.0 were kept for RNA-seq library construction. Each sample was quantified using a Nanodrop 1000 spectrophotometer (Thermo Scientific, Wilmington, DE, USA) and, prior to constructing RNA-seq libraries, 1.5 µg of RNA was enriched in poly-A RNA using magnetic beads with poly-T oligonucleotides. Enriched poly-A RNA was used to construct libraries using the Illumina TrueSeq RNA sample prep kit v2 (llumina, San Diego, Ca, USA) as per the manufacturer’s instructions except that the RNA fragmentation step was performed during six minutes. Replicate libraries were indexed with a unique barcode identifier and then quantified and mixed to form a normalized 12-plex paired-end sequencing library. A single lane (50-nt paired-end reads) of an Illumina Hi-Sequation 2000 instrument (Illumina Inc, San Diego, CA, USA) was used to sequence the entire library at McGill University-Genome Quebec Innovation Centre (Montréal, Canada).

### Data processing, analysis of differential gene expression and gene clustering

Using Trimmomatic v0.33 (Bolger *et al.* 2014), raw paired-end reads were pre-processed at a Phred quality score threshold of ≥25 for the 3′ end and trimmed reads shorter than 25 nt were discarded. Trimmed reads were aligned to the barley reference genome (Hv_IBSC_PGSB_v2.dna.toplevel; ftp://ftp.ensemblgenomes.org/pub/release-36/) using Tophat v2.1.1 (Kim *et al.* 2013). Using HTSeq v0.6.1p1 (Anders *et al.* 2014), we calculated the number of reads mapped to exons using the reference transcriptome and generated a read-count matrix. Prior to performing differential gene expression analysis, we filtered the read-count matrix and kept only genes with ≥5 reads per million for a minimum of 4 samples and normalized gene expression based on the TMM method using edgeR (Robinson *et al.* 2010; Robinson and Oshlack 2010). In edgeR, we performed a multidimensional scaling (MDS) analysis to assess the degree of uniformity among replicates of the three developmental stages. Using edgeR, we examined the overlap among genes at the three stages of microspore development to identify genes exclusively expressed at a specific stage of development and clustered them under a category named ON/OFF genes. We then used edgeR to identify differentially expressed genes (DEGs) and measured the significance of expression changes using the generalized linear model (glm) test for two developmental transitions: (i) from day 0 to day 2 and (ii) from day 2 to day 5. Results were filtered and considered as significant if both log_2_FC ≥ |2.0| and *q-value* ≤ 0.01 were observed. Finally, DEGs were clustered in eight groups representing their expression pattern (while the expression of a gene can go up, down or remain unchanged) between days 0 to 5 (successively from day 0 to day 2 and from day 2 to day 5).

### Gene functional annotation

We retrieved functional annotation of barley genes using BioMart v0.7 available on Phytozome (https://phytozome.jgi.doe.gov) for KEGG functional orthologs (KO) and Panther description. Reference orthologous genes were retrieved using the online EMBL-EBI HMMER program (https://www.ebi.ac.uk/Tools/hmmer/) with the SwissProt database restricted to the *A. thaliana* and *O. sativa* species.

### Data availability

The complete set of raw and mapped RNA-seq reads were deposited in the Sequence Read Archives (SRA) under accession number SRP127768.

## RESULTS AND DISCUSSION

### Isolation and purification of microspores at three key stages of IMC

**Figure 1:**
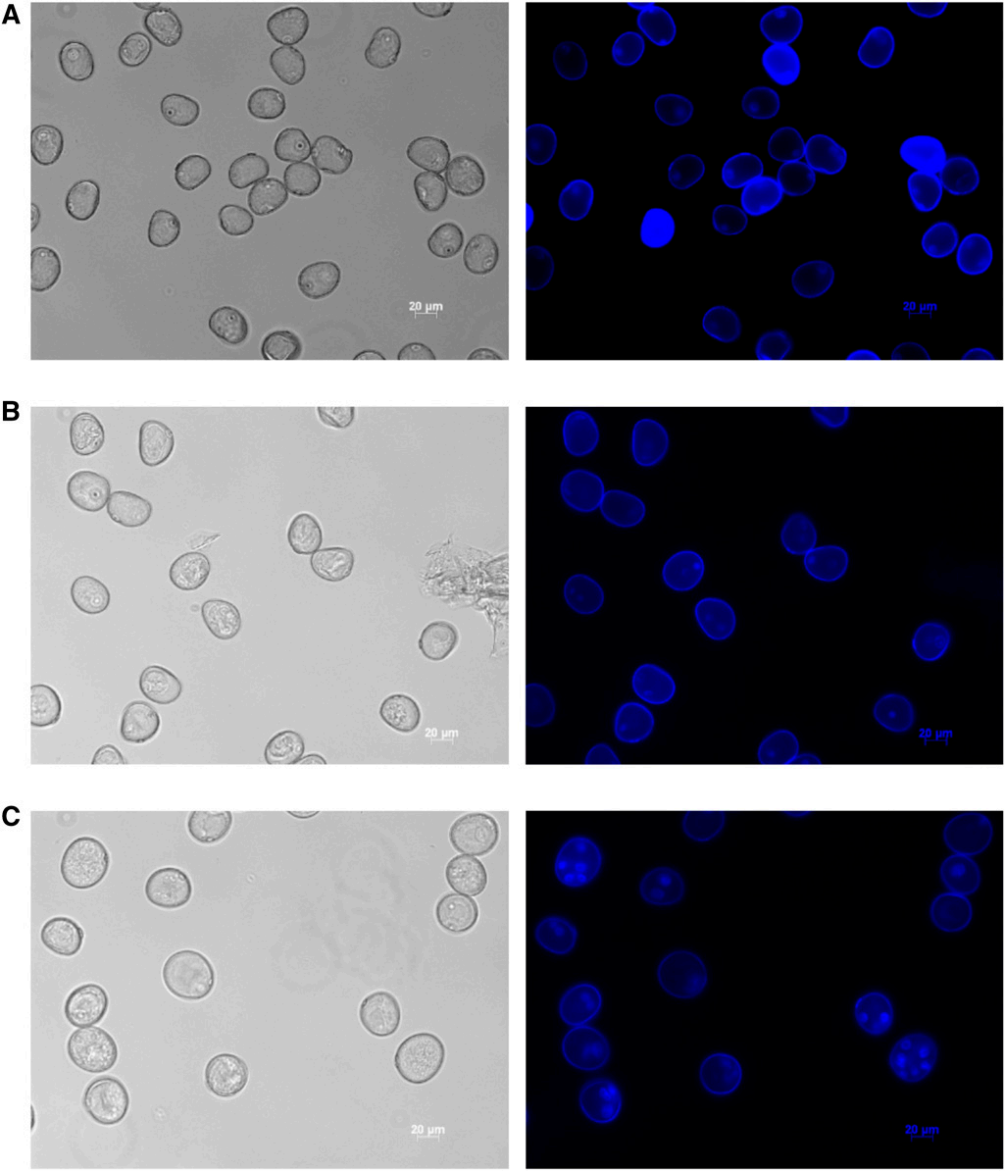
Representative samples of microspores obtained from the cv. Gobernadora at the three stages of early isolated microspore culture. (a) Mid-late to late uninucleate microspores (day 0), (b) enlarged microspores (day 2) and (c) star-like microspore to multi-cellular structure microspore phenotype (day 5). Images were captured using a Zeiss Apoptome microscope under UV laser illumination (excitation of 390/22 nm and emission of 460/50 nm) at a 20x magnification without staining (left) and with DAPI staining (right).

To better understand the changes in gene expression that underlie the developmental switch that occurs during isolate microspore culture (IMC) in barley, we used the cv. Gobernadora known as one of the most responsive genotypes in anther culture (Marchand *et al.* 2008) and we characterized the transcriptome of microspores at days 0, 2 and 5. As can be seen in Figure 1, without and with DAPI staining, on day 0, microspores were characterized by a single nucleus positioned across the cell wall (Figure 1a) corresponding to the late uninucleate stage known as the most embryogenic-responsive microspore (Kasha *et al.* 2001); on day 2, a little enlarged microspores showed a single nucleus migrating toward the center of the cells as well as a few microspores that had two nuclei (Figure 1b) and (iii) on day 5, some star-like microspores as well as many, more advanced, multicellular structures (MCSs) exhibiting 2 to 6 nuclei were seen (Figure 1c). No or very few damaged or dead cells were observed. Relative to the phenotypes described by Maraschin (2005), our samples can be described as highly similar to day 0, slightly more advanced at the enlarged microspore (EM) stage (day 2) and exceeded in many cases the star-like microspore (SL) stage (day 5) and mostly corresponded to the multicellular structure (MCS) stage indicating that microspores had engaged in embryogenesis. Such MCSs were typically observed 2 to 5 days later by Maraschin (2005). We suggest that the faster microspore development observed could be attributed to three factors: (i) a superior responsiveness of the genotype Gobernadora or (ii) the use of a more efficient stress pretreatment or (iii) the use of a better induction medium.

### Transcriptomes at three stages of isolated microspore culture

**Figure 2:**
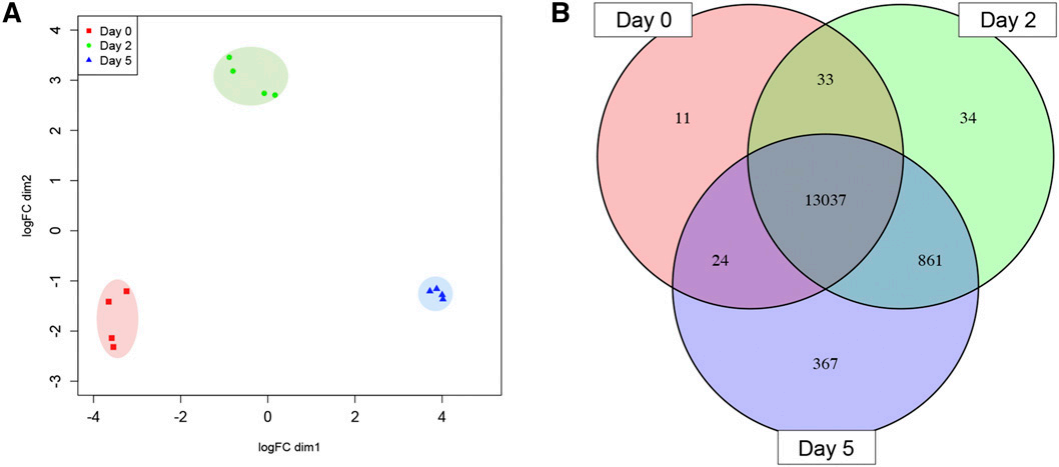
Variation in gene expression profiles within and between microspore samples. An MDS plot shows the high degree of uniformity among replicates of the same stage as well as the distinctness between stages (a) and a Venn diagram illustrates the high degree of overlap among genes expressed in the different cell types (b).

To obtain a comprehensive overview of the set of genes expressed in microspores during the early stages of IMC, we used an RNA-seq approach. Four biological replicates at each stage yielded a total of more than 279M 50-nt paired-end reads, for an average of ∼92M reads per cell type. To assess the degree of uniformity among replicates, we performed an MDS analysis based on genome-guided transcriptome assemblies. As can be seen in Figure 2a, we observed three highly distinctive and very tight clusters indicating that the different replicates were tremendously uniform and that each stage has a distinct transcriptomic signature. Even if some morphological heterogeneity was observed among day-5 microspore replicates, the tightest clustering of these replicates in the MDS plot suggested an enrichment of cells having a high homogeneity in their transcriptional response to embryogenesis induction.

Overall the analysis, a total of 14,367 genes was detected when summed across all three stages and the vast majority of these genes (13,037; 90.7%) were expressed at all three developmental stages (Figure 2b), although not necessarily in equal abundance. Among individual stages, we observed an increase in the number of genes expressed in microspores from a low of 13,105 at day 0, to 13,965 at day 2 and a maximum of 14,289 at day 5. Although small (2.9%), the number of genes expressed exclusively at a specific stage increased from 11 (day 0) to 34 (day 2) to 367 (day 5). Finally, the number of genes expressed in two of the stages was also relatively small (6.4%), ranging from 24 (day 0 and day 5) to 861 (day 2 and day 5). In the next section, we describe further some of the broad metabolic pathways in which genes expressed exclusively at a stage are involved. Mainly due to their microarray (1,421 ESTs) designed to interrogate genes expressed in early barley zygotic embryogenesis, Maraschin *et al.* (2006) detected a lower number of transcripts (418) representing only 2.9% of the genes interrogated in our work. In wheat microspores at comparable stages, Seifert *et al.* (2016) reported similar results both in terms of the number of genes (15,598) and the overlap of genes expressed across all developmental stages (11,765; 90.4%). These authors also observed an increase in the number of genes expressed in microspores from developmental stage 1 (14,470; similar to day 0 in this work) to stage 2 (14,666; day 2 here), while, in contrast, they reported a slightly decrease at stage 3 (13,128; similar to day 5). They also detected a small (4.5%) number of genes expressed exclusively at a single stage: 24, 11 and 666 in stages 1, 2 and 3, respectively. In conclusion, we succeeded in isolating highly uniform populations of microspores at three phenotypically distinct stages of IMC and, while each exhibited a distinct transcriptomic profile, the number of genes underlying these different profiles via their presence or absence was rather small (dozens to at most a thousand).

### **G**enes exclusively expressed in microspores at day 5 provided highlight About metabolism governing the embryogenesis induction

To provide a comprehensive overview of genes expressed exclusively at a specific stage of development, we explored metabolisms governed by these genes. While no or too low functional annotations were available for genes exclusively expressed in microspores at day 0 (only 11 genes) and 2 (34 genes), we limited the investigation to the 367 genes specifically expressed to microspores at day 5 and considered them as potential marks of the induction of embryogenesis. Interestingly, we observed genes (Table 1) encoding transcription factors such as *BABY BOOM* (*BBM*; HORVU3Hr1G089160), *AINTEGUMENTA-like 5* (*AIL5*; HORVU7Hr1G111060) and *WUSCHEL-RELATED HOMEOBOX 4* (*WUS*; HORVU5Hr1G022120). *BBM* and *AIL5* are both AP2/ERF transcription factors known to be expressed early and to play important roles in embryo development in *B. napus* (Boutilier *et al.* 2002) and *A. thaliana*, respectively (Tsuwamoto *et al.* 2010). Orthologs of these genes were recently reported to be turn on early during microspores culture in wheat (Seifert and al. 2016). *WUS* is a homeobox transcription factor reported to play an essential role in maintaining cells in a state of proliferation and responsiveness to other developmental cues (Mayer *et al.* 1998; Gallois *et al.* 2002; Smertenko and Bozhkov 2014) in addition to inducing the vegetative-to-embryonic transition in *A. thaliana* somatic embryogenesis (Zuo *et al.* 2002; Smertenko and Bozhkov 2014). Together, the expression induction of these three genes in microspores at day 5 suggests the microspore commitment in the embryogenesis developmental pathway and is consistent with the phenotype previously observed.

**Table 1:** Subset of genes that were newly and specifically in microspores at day 5

	**Reference gene**	**Reference specie**	**KEGG reference**	**Barley gene name**
**Transcription factor**				
Baby boom	BBM1	B. Napus		HORVU3Hr1G089160
Aintegumenta-like 5	AIL5	A. thaliana		HORVU7Hr1G111060
Wuschel-related homeobox 5	WOX5	O. sativa		HORVU5Hr1G022120
**Hormones biogenesis**				
Linoleate 13S-lipoxygenase	LOX8	O. sativa	EC:1.13.11.12	HORVU7Hr1G050660
Linoleate 13S-lipoxygenase	LOX8	O. sativa	EC:1.13.11.12	HORVU7Hr1G050670
Xanthoxin dehydrogenase	ABA2	A. thaliana	EC:1.1.1.288	HORVU2Hr1G015140
**Plant hormone signal transduction**				
Auxin-responsive protein	IAA15	O. sativa	K14484	HORVU1Hr1G025670
Auxin-responsive protein	IAA26	O. sativa	K14484	HORVU5Hr1G081180
Auxin-responsive protein	IAA20	O. sativa	K14484	HORVU7Hr1G026970
Indole-3-acetic acide-amido synthetase	GH3.2	O. sativa	K14487	HORVU1Hr1G066340
Indole-3-acetic acide-amido synthetase	GH3.4	O. sativa	K14487	HORVU3Hr1G074230
Cyclin D3	CYCD3-2	O. sativa	K14505	HORVU5Hr1G050270
Abscisic acid receptor PYR/PYL	PYL	A. thaliana	K14496	HORVU1Hr1G050110
Ethylene receptor 4	ETR4	O. sativa	EC:2.7.13.-	HORVU6Hr1G071860
Histidine kinase 2/3/4 (cytokinin receptor)	HK3	O. sativa	K14489	HORVU3Hr1G094870

Then, we inspected this set of 367 genes to look for other hallmarks of microspore transition not previously reported in microspore embryogenesis. The major finding was the detection of genes belonging to hormonal biosynthetic pathways in addition to genes involved in plant hormones signal transduction processes (Table 1). For hormone biogenesis (Table 1), two genes encoding the *linoleate 13S-lipoxygenase* enzyme (*LOX8*; HORVU7Hr1G050660 and HORVU7Hr1G050670) known as the first enzyme involved in the synthesis of jasmonic acid (Wasternack 2007) were detected. Similarly, a gene encoding xanthoxin dehydrogenase (*ABA2*; HORVU2Hr1G015140) enzyme, involved in the abscisic acid biogenesis pathway was also detected only at day 5. Of plant hormones signal transduction pathways, we detected numerous genes involved in the signal transduction mediated by the cytokinin, abscisic acid, auxin, ethylene and brassinosteroid (Table 1). Of these genes, we detected few hormonal receptors homologous to the *Arabidopsis PYL1* (HORVU1Hr1G050110) and the rice *ETR4* (HORVU6Hr1G071860) and *HK3* (HORVU3Hr1G094870) genes known to respectively encode abscisic acid, ethylene and a cytokinin receptors required to mediate the hormone transduction response. In addition, numerous genes involved in the signaling cascade related to auxin were detected such as *IAA15* (HORVU1Hr1G025670), *IAA26* (HORVU5Hr1G081180), *IAA20* (HORVU7Hr1G026970) genes as well as *GH3.2* (HORVU1Hr1G066340) and *GH3.4* (HORVU3Hr1G074230) genes known as induced by this signal transduction pathway. Finally, we detected the expression a gene homolog to the rice *CYCD3-2* (HORVU5Hr1G050270) gene known as induced by the brassinosteroid hormone signal transduction and involved on cell division (Hu *et al.* 2000).

Interestingly, Żur *et al.* (2015a and 2015b) reported that the endogenous level of natural hormones (auxin/cytokinin/ABA) and its balance with exogenously applied hormones can be crucial both for the yield and quality of microspore-derived embryos and they suggested that hormonal homeostasis might be one of the most important factors determining cell embryogenic competency. Also, it was demonstrated that an addition of these three hormones to microspores in culture significantly increased embryogenesis when applied a couple of hours from the beginning of microspore culture (Ahmadi *et al.* 2014). Since only cytokinin and auxin were added to our culture medium, our results suggest that microspores of barley cv. Gobernadora can potentially rapidly activate hormone biogenesis and mediate hormonal signal transduction to other hormones within the first days of culture. This could explain in part its high aptitude to form embryos. Together, these results suggest that the transition toward embryogenesis involves the expression of genes governing hormonal biogenesis and signal transduction pathways. These genes could potentially serve as biomarkers to compare the efficiency of induction of embryogenesis following various pretreatments or in different genetic backgrounds.

### DEG analysis and clustering

In addition to genes that were only detected at a specific stage, changes occurring in the abundance of transcripts could help understand the developmental shift undergone by these microspores. In principle, when the developmental fate of the uninucleate microspore is shifted from pollen formation to embryogenesis, we would expect to see a decrease in expression of genes associated with pollen formation and an increase in genes associated with embryogenesis. Thus, differentially expressed genes (DEGs) could shed light on the key metabolic changes driving this switch. Overall, a total of 3,382 DEGs (23.5%) were significantly over- or under-expressed; of these genes, 2,155 and 2,281 DEGs, respectively, were identified when contrasting expression at day 0 and day 2, and when comparing day 2 and day 5. For both categories of DEGs, most transcripts were up-regulated (80.4% and 66.7%, respectively). Our results are similar to those reported in wheat by Seifert *et al.* (2016) in terms of the number of DEGs when contrasting comparable stages of microspore development (6,385 out of 15,598), but contrast markedly in terms of up- or down-regulation. Indeed, these authors reported only 33.3% and 43.0% of up-regulated genes for the two transitions. This low proportion of up-regulated genes is rather atypical of what observed here or previously reported in barley (Maraschin *et al.* 2006).

**Figure 3:**
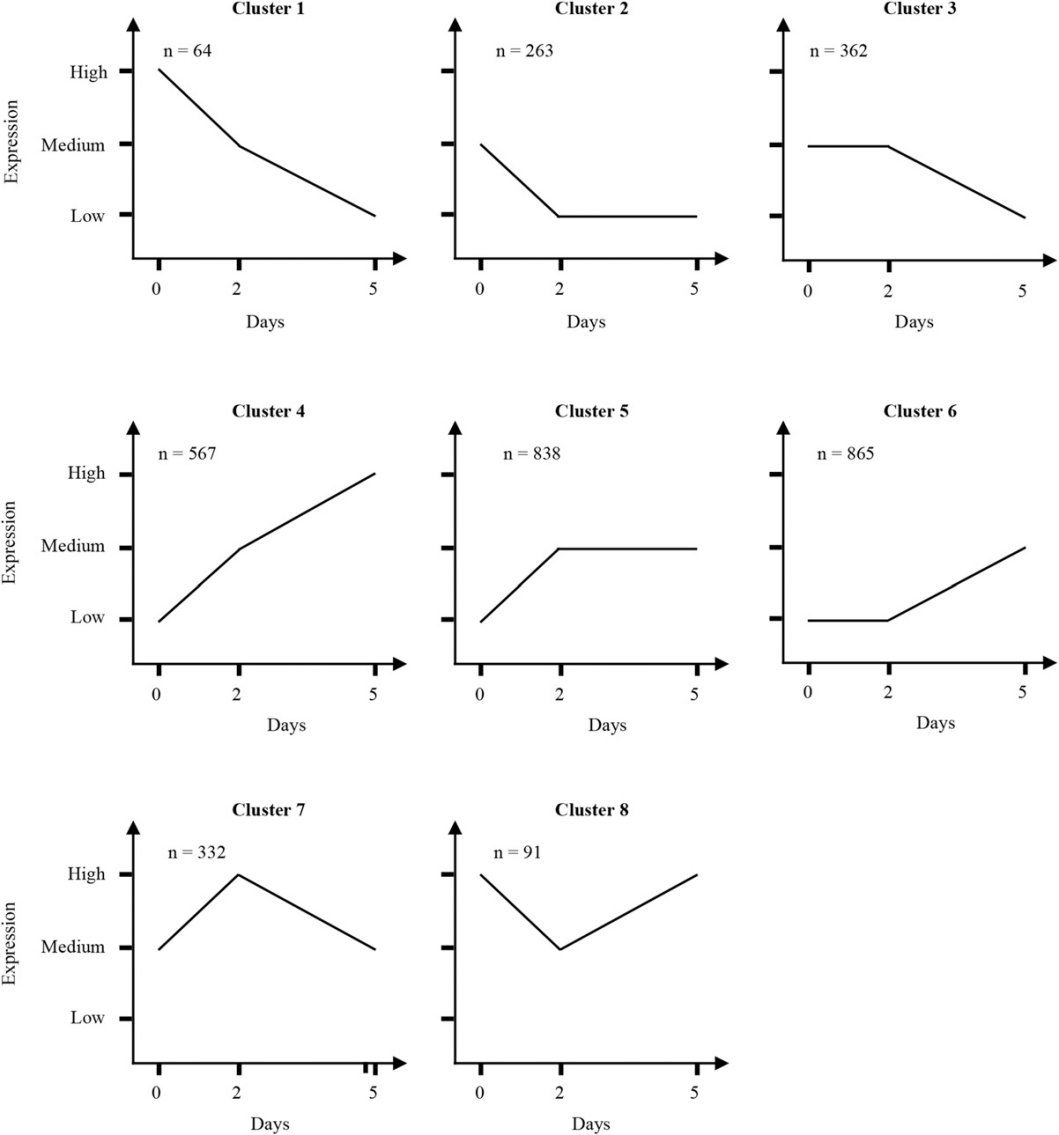
Schematic clusters representing different patterns of gene expression among differentially expressed genes (DEGs). Clusters were intuitively generated by integrating each gene expression pattern (expression of a gene can go up, down or remain unchanged) at both developmental transitions (from day 0 to day 2 and from day 2 to day 5) producing a total of eight different expression patterns.

To facilitate the analysis of this large set of DEGs, we grouped them into eight profiles (illustrated in Figure 3). Below, we describe broad metabolic changes associated with these various clusters of genes sharing a similar expression profile based on the KEGG and Panther annotations (the complete annotation is detailed in Table S1, Table S2, Table S3, Table S4, Table S5, Table S6, Table S7, and Table S8 corresponding to each gene clusters).

### Metabolisms controlled by differentially expressed genes in microspore from day 0 and 2

Of the eight clusters, three (4, 5 and 7) shared an up-regulation of genes between days 0 and 2, but only the latter two exhibited an increase occurring only at this stage. Among these, we found numerous genes belonging to four main categories: carbohydrate metabolism, hormone-modulated gene expression, proteolytic genes, cytoprotection and hormone biogenesis. These have been proposed to participate in the interruption of pollen development (Maraschin *et al.* 2005). Despite being reported in much greater numbers here, examples of such genes (especially among the first two categories) have already been described in barley by Maraschin *et al.* (2006). In what follows, we focus on genes involved in cytoprotection, proteolysis and genes related to hormone biogenesis, three previously poorly described categories.

Of genes involved in cytoprotection (Table 2), we detected nine genes coding *Glutathione S-transferases* (*GSTs*) and thirteen heat shock proteins (*HSPs*), both, known to be induced in response to stress and involved in cytoprotection. Specifically, GSTs act in the glutathione-mediated detoxification metabolism protecting cells against reactive oxygen species (ROS) (Chen and Singh 1999; Garretón *et al.* 2002). This result is concordant with the findings of Maraschin *et al.* (2006) who reported the induction of a *GST* gene in microspores subjected to stress. Heat shock proteins (*HSPs*) are known to act in various ways to protect proteins such as by assisting in refolding, preventing aggregation or by acting as a co-chaperone (Park and Seo 2015) Previously, members of the *HSP* family have been reported to be highly expressed in microspores subjected to heat and starvation stresses: *HSP90* in *Brassica* (Seguí-Simarro *et al.* 2003), *HSP70* in *Brassica* (Cordewener *et al.* 1997; Seguí-Simarro *et al.* 2003) and *Capsicum* (Bárány *et al.* 2001), as well as *HSP20* in tobacco (Zarsky *et al.* 1995). The two latter were detected in barley for the first time in this work, but with a distinct expression pattern. The 9 *HSP20* genes were up-regulated from day 0 to day 2 (Figure 3; Cluster 5) while the five *HSP70* genes were initially up-regulated and then down-regulated from day 2 to day 5 (Figure 3; cluster 7). In addition to *HSPs*, we detected a gene encoding heat shock transcription factor (*HSF*) known to increase the transcriptional response of *HSP* genes (barley homolog of the HSFA4A (HORVU1Hr1G081300) gene).

**Table 2:** List of up-regulated genes in microspores from day 0 to day 2

**Gene involved in cytoprotection**	**Reference gene**	**Reference specie**	**KEGG reference**	**Barley gene name**
Glutathione S-transferase	GSTUD	O. sativa	EC:2.5.1.18	HORVU1Hr1G021170
Glutathione S-transferase	GSTUI	O. sativa	EC:2.5.1.18	HORVU5Hr1G058000
Glutathione S-transferase	GSTUI	O. sativa	EC:2.5.1.18	HORVU1Hr1G049250
Glutathione S-transferase	GSTUI	O. sativa	EC:2.5.1.18	HORVU1Hr1G049190
Glutathione S-transferase	GSTUH	O. sativa	EC:2.5.1.18	HORVU3Hr1G095670
Glutathione S-transferase	GSTUP	O. sativa	EC:2.5.1.18	HORVU5Hr1G103420
Glutathione S-transferase	GSTU3	O. sativa	EC:2.5.1.18	HORVU4Hr1G057910
Glutathione S-transferase	GSTU8	O. sativa	EC:2.5.1.18	HORVU3Hr1G107350
Glutathione S-transferase	GSTU8	O. sativa	EC:2.5.1.18	HORVU1Hr1G064890
HSP20 family protein	HS16	O. sativa		HORVU0Hr1G020420
HSP20 family protein	HS16A	O. sativa		HORVU3Hr1G007500
HSP20 family protein	HS16A	O. sativa		HORVU3Hr1G007380
HSP20 family protein	HS16A	O. sativa		HORVU2Hr1G120170
HSP20 family protein	HS16A	O. sativa		HORVU3Hr1G006530
HSP20 family protein	HSP17A	O. sativa		HORVU4Hr1G060760
HSP20 family protein	HSP232	O. sativa		HORVU2Hr1G077710
HSP20 family protein	HSP219	O. sativa		HORVU4Hr1G015170
HSP20 family protein	HS26P	O. sativa		HORVU4Hr1G063350
HSP70 family protein 5	BIP5	O. sativa		HORVU2Hr1G122760
HSP70 family protein 5	BIP5	O. sativa		HORVU5Hr1G078400
HSP70 family protein 5	BIP5	O. sativa		HORVU7Hr1G098810
HSP70 family protein 5	MD37A	A. thaliana		HORVU7Hr1G107190

Some of the proteolytic genes identified encode components of the proteasome and the ubiquitin-mediated proteolysis system as well as proteases. We detected an induction of proteolytic genes homologous to components of the 26S proteasome regulatory complex such as the ATPase RPT4 (HORVU4Hr1G027260) and PSMD10 (HORVU3Hr1G023740; HORVU3Hr1G033250; HORVU7Hr1G029900) as well as the proteasome endopeptidase complex (HORVU5Hr1G109720). In addition, we detected a few genes encoding a ubiquitin-ligase (UB-E3) protein. While expressed, no changes in expression were detected for the ubiquitin-activating (UB-E1) and the ubiquitin-conjugating (UB-E2) enzymes. In addition, numerous proteases were detected such as the cathepsin B-like protease 2 (CATHB2; HORVU4Hr1G010300), the serine carboxypeptidase 1 (CBP1; HORVU3Hr1G096830), the aminopeptidase M1 (APM1; HORVU5Hr1G057330), the endopeptidase Clp (CLPR1; HORVU1Hr1G094480) and numerous the aspartyl proteases (14 genes) such as the HORVU3Hr1G056630 gene coding for a phytepsin protein previously reported by Maraschin and al (2006). In wheat, Seifert *et al.* (2016) did not report the activation of such enzymes and metabolisms; rather, they reported an over-representation of genes related to proteolysis among a cluster of down-regulated genes when microspores underwent the transition toward the third stage of development studied.

Three clusters (1, 2 and 8) shared a down-regulation of genes between days 0 and 2, but only the second exhibited a decrease occurring exclusively at this stage (genes that decrease between days 0 and 2 and stay low expressed). Among these, the most highly represented categories were genes encoding proteins involved in genetic information processing and DNA organization such as nucleosome assembly factors. For genetic information processing, many genes (Table 3) involved in translation coding the small ribosomal subunit (a total of 7 genes encoding distinct proteins) or the large ribosomal subunit (a total of 15 genes) were observed. Down-regulation of these genes (a total of 22) coding for ribosomal components upon application of a stress is consistent with previous reports (Rashid *et al.* 1982, Hoekstra *et al.* 1992, Telmer *et al.* 1995, Maraschin *et al.* 2005). Nonetheless, Maraschin *et al.* (2006) detected an increased expression of the gene coding the 60S ribosomal protein L26A. While a gene coding exactly the same ribosomal protein was not detected in our experiment, our more extensive data suggests strongly a down-regulation of genes coding ribosomal proteins. In agreement with our results, Malik *et al.* (2007) also observed a down-regulation of several genes encoding the small and large ribosomal subunits in *B. napus* microspores after three days of heat and osmotic pretreatment. In addition to ribosomal protein genes, we detected a down-regulation of three homologs of the *A. thaliana* NOP5A (HORVU2Hr1G022140 and HORVU1Hr1G083960) and FIB1 (HORVU6Hr1G091860) genes, respectively coding for the nucleolar protein 56 and the rRNA 2’-O-methyltransferase fibrillarin protein (Table 3), all known to be required for 60S ribosomal subunit biogenesis. Concordantly, Maraschin *et al.* (2006) also observed a down-regulation of the gene coding for the fibrillarin protein in barley microspores after stress pretreatment. For the second category, we detected a total of 7 genes (Table 3) encoding all core histone components of the nucleosome: histones H2A (HORVU3Hr1G116550, HORVU4Hr1G008800, HORVU4Hr1G058940, HORVU6Hr1G092280), H2B (HORVU3Hr1G086610), H3 (HORVU1Hr1G020050) and H4 (HORVU1Hr1G017830). Although that we observe few microspores having initiated a nuclear division at day 2, a reduction of expression for genes encoding these proteins may reflect the arrest of the pollen formation that involve two successive mitotic division during the gametogenesis.

**Table 3:** List of down-regulated genes in microspores from day 0 to day 5

	**Reference gene**	**Reference specie**	**KEGG reference**	**Barley gene name**
**Genes involved in translation**				
40S ribosomal protein	RS4	O. sativa		HORVU1Hr1G021720
40S ribosomal protein	RS62	A. thaliana		HORVU2Hr1G029890
40S ribosomal protein	RS8	O. sativa		HORVU2Hr1G067370
40S ribosomal protein	RS92	A. thaliana		HORVU2Hr1G028510
40S ribosomal protein	RS101	A. thaliana		HORVU3Hr1G111760
40S ribosomal protein	RS174	A. thaliana		HORVU1Hr1G042220
40S ribosomal protein	RS26	O. sativa		HORVU5Hr1G111820
60S ribosomal protein	RL81	A. thaliana		HORVU4Hr1G077020
60S ribosomal protein	RL81	A. thaliana		HORVU5Hr1G021730
60S ribosomal protein	RLA0	O. sativa		HORVU7Hr1G073720
60S ribosomal protein	RLA25	A. thaliana		HORVU0Hr1G004480
60S ribosomal protein	RL3	O. sativa		HORVU4Hr1G019980
60S ribosomal protein	RL4A	A. thaliana		HORVU4Hr1G075710
60S ribosomal protein	RL51	O. sativa		HORVU5Hr1G092630
60S ribosomal protein	RL63	A. thaliana		HORVU6Hr1G052600
60S ribosomal protein	R10A1	A. thaliana		HORVU3Hr1G084310
60S ribosomal protein	RL171	A. thaliana		HORVU5Hr1G052280
60S ribosomal protein	RL18A	O. sativa		HORVU1Hr1G088040
60S ribosomal protein	RL212	A. thaliana		HORVU4Hr1G084420
60S ribosomal protein	RL321	A. thaliana		HORVU5Hr1G075420
60S ribosomal protein	RL371	O. sativa		HORVU3Hr1G062590
60S ribosomal protein	RL371	O. sativa		HORVU7Hr1G081910
**Genetic information processing**				
Nucleolar protein 56	NOP5A	A. thaliana		HORVU2Hr1G022140
Nucleolar protein 56	NOP5A	A. thaliana		HORVU1Hr1G083960
rRNA 2’-O-methyltransferase fibrillarin	FIB1	A. thaliana	EC:2.1.1.-	HORVU6Hr1G091860
**Nucleosome assembly factors**				
Histone H2A	H2A5	O. sativa		HORVU4Hr1G058940
Histone H2A	H2A5	A. thaliana		HORVU3Hr1G116550
Histone H2A	H2AXA	O. sativa		HORVU4Hr1G008800
Histone H2A	H2AXB	O. sativa		HORVU6Hr1G092280
Histone H2B	H2B11	O. sativa		HORVU3Hr1G086610
Histone H3	H32	A. thaliana		HORVU1Hr1G020050
Histone H4	H4	A. thaliana		HORVU1Hr1G017830

### Metabolisms controlled by differentially expressed genes in microspore from day 2 and 5

When then expanded our investigation to the other up-regulated DEGs between days 2 and 5 (clusters 6 and 8), *i.e.*, those that were also expressed at earlier stages but less strongly. First, numerous genes encoding enzymes involved in secondary metabolism were found, including enzymes of the phenylpropanoid pathway. For instance, we detected many genes encoding enzymes such as *shikimate O-hydroxycinnamoyltransferase* (*HCT1*; HORVU2Hr1G086380), *phenylalanine ammonia-lyase* (*PAL2*; HORVU2Hr1G038140, HORVU2Hr1G038120, HORVU0Hr1G016330), *cinnamoyl-CoA reductase* (*CCR1*; HORVU7Hr1G030380) and *4-coumarate–CoA ligase* (*4CL3*; HORVU6Hr1G030390 and *4CL4*; HORVU7Hr1G111130). In addition to the one mentioned above, we detected two supplementary genes encoding a *linoleate 13S-lipoxygenase* (homologous to *LOX6* and *LOX8*) and a *linoleate 9S-lipoxygenase* (homologous to *LOX4*) (Table 1). In addition to these enzymes, we detected *allene oxide synthase* (*AOS1*; HORVU5Hr1G098090 and *AOS2*; HORVU4Hr1G066270), *allene oxide cyclase* (*AOC*; HORVU6Hr1G081000), acyl-CoA oxidase (*ACOX2*; HORVU7Hr1G029110 and *ACOX3*; HORVU7Hr1G083490) and 12-oxophytodienoic acid reductase (*OPR7*; HORVU7Hr1G095960) genes, all involved in jasmonic acid synthesis. Jacquard *et al.* (2009) similarly reported an up-regulation of genes encoding enzymes involved in the synthesis of jasmonic acid and in the phenylpropanoid pathway in barley anther culture. In contrast, in wheat, Seifert *et al.* (2016) did not report the activation of such enzymes and metabolisms. It remains too early to establish direct roles for such genes and pathways as functional analyses would need to be conducted to validate their function in microspore embryogenesis.

Previously, we reported a reduction in the expression of genes encoding both ribosomal protein subunits and nucleosome assembly components during the transition from day 0 to 2. It is interesting to observe a massive increase in the expression of genes encoding these proteins as the microspore transitions from day 2 to day 5. Indeed, we observed a total of 13 genes contributing to the synthesis of the small and large ribosomal subunits with, respectively, 2 and 11 genes (Table 4). However, these ribosomal genes encode the 30S and 50S subunits that make up the 80S ribosome found in organelles such as the chloroplast and mitochondria. In addition, we detected a total of 38 genes (Table 4) encoding all core histone components of the nucleosome: histones H2A (8 genes), H2B (5 genes), H3 (13 genes) and H4 (12 genes). Since we observe that a majority of microspores have initiated a nuclear division by day 5, an increase in the expression of genes encoding these proteins may reflect that the microspore is preparing for a first cell division as a first step in the embryogenic pathway.

**Table 4:** List of up-regulated genes in microspores from day 2 to day 5

	**Reference gene**	**Reference specie**	**KEGG reference**	**Barley gene name**
**Phenylpropanoid biogenesis pathway**				
Cinnamoyl-CoA reductase	CCR1	A. thaliana	EC:1.2.1.44	HORVU7Hr1G030380
4-coumarate–CoA ligase	4CL3	O. sativa	EC:6.2.1.12	HORVU6Hr1G030390
4-coumarate–CoA ligase	4CL4	O. sativa	EC:6.2.1.12	HORVU7Hr1G111130
Phenylalanine ammonia-lyase	PAL2	O. sativa	EC:4.3.1.24	HORVU2Hr1G038140
Phenylalanine ammonia-lyase	PAL2	O. sativa	EC:4.3.1.24	HORVU2Hr1G038120
Phenylalanine ammonia-lyase	PAL2	O. sativa	EC:4.3.1.24	HORVU0Hr1G016330
Shikimate O-hydroxycinnamoyltransferase	HCT1	O. sativa	EC:2.3.1.133	HORVU2Hr1G086380
**Hormones biogenesis**				
12-oxophytodienoic acid reductase	OPR7	O. sativa	EC:1.3.1.42	HORVU7Hr1G095960
Linoleate 9S-lipoxygenase 4	LOX4	O. sativa	EC:1.13.11.12	HORVU4Hr1G005920
Linoleate 13S-lipoxygenase 6	LOX6	O. sativa	EC:1.13.11.12	HORVU4Hr1G076570
Linoleate 13S-lipoxygenase 8	LOX8	O. sativa	EC:1.13.11.12	HORVU7Hr1G050680
Allene oxide synthase	AOS1	O. sativa	EC:4.2.1.92	HORVU5Hr1G098090
Allene oxide synthase	AOS2	O. sativa	EC:4.2.1.92	HORVU4Hr1G066270
Allene oxide cyclase	AOC	O. sativa	EC:5.3.99.6	HORVU6Hr1G081000
Acyl-CoA oxidase	ACOX2	A. thaliana	EC:1.3.3.6	HORVU7Hr1G029110
Acyl-CoA oxidase	ACOX3	A. thaliana	EC:1.3.3.6	HORVU7Hr1G083490
**Genes involved in translation**				
30S ribosomal protein S17, chloroplastic	RR17	O. sativa		HORVU7Hr1G115040
30S ribosomal protein S5, chloroplastic	RR5	A. thaliana		HORVU4Hr1G038570
50S ribosomal protein L10, chloroplastic	RK10	A. thaliana		HORVU4Hr1G057450
50S ribosomal protein L11, chloroplastic	RK11	A. thaliana		HORVU4Hr1G084830
50S ribosomal protein L12, chloroplastic	RK12	O. sativa		HORVU3Hr1G059810
50S ribosomal protein L13, chloroplastic	RK13	A. thaliana		HORVU3Hr1G071530
50S ribosomal protein L19-2, chloroplastic	RK192	A. thaliana		HORVU6Hr1G062040
50S ribosomal protein L27, chloroplastic	RK27	O. sativa		HORVU3Hr1G095330
50S ribosomal protein L28, chloroplastic	RK28	A. thaliana		HORVU1Hr1G000040
50S ribosomal protein L3-1, chloroplastic	RK3A	A. thaliana		HORVU6Hr1G018830
50S ribosomal protein L31, chloroplastic	RK31	A. thaliana		HORVU3Hr1G056580
50S ribosomal protein L6, chloroplastic	RK6	A. thaliana		HORVU4Hr1G040950
50S ribosomal protein L9, chloroplastic	RK9	A. thaliana		HORVU6Hr1G093030
**Nucleosome assembly factors**				
Histone H2A	H2AV2	O. sativa		HORVU1Hr1G035130
Histone H2A	H2A4	O. sativa		HORVU6Hr1G011490
Histone H2A	H2A5	O. sativa		HORVU7Hr1G100100
Histone H2A	H2A5	O. sativa		HORVU7Hr1G030120
Histone H2A	H2A5	O. sativa		HORVU1Hr1G005870
Histone H2A	H2A5	O. sativa		HORVU6Hr1G029220
Histone H2A	H2A5	O. sativa		HORVU6Hr1G009020
Histone H2A	H2AXB	O. sativa		HORVU7Hr1G112470
Histone H2B	H2B7	O. sativa		HORVU4Hr1G073130
Histone H2B	H2B7	O. sativa		HORVU1Hr1G058500
Histone H2B	H2B7	O. sativa		HORVU1Hr1G078530
Histone H2B	H2B9	O. sativa		HORVU1Hr1G085540
Histone H2B	H2B7	O. sativa		HORVU1Hr1G049920
Histone H3	H32	A. thaliana		HORVU4Hr1G067970
Histone H3	H32	A. thaliana		HORVU1Hr1G074340
Histone H3	H32	A. thaliana		HORVU1Hr1G022400
Histone H3	H32	A. thaliana		HORVU3Hr1G063270
Histone H3	H32	A. thaliana		HORVU6Hr1G031580
Histone H3	H32	A. thaliana		HORVU7Hr1G024990
Histone H3	H32	A. thaliana		HORVU7Hr1G025160
Histone H3	H32	A. thaliana		HORVU7Hr1G100450
Histone H3	H32	A. thaliana		HORVU1Hr1G073670
Histone H3	H32	A. thaliana		HORVU1Hr1G058490
Histone H3	H32	A. thaliana		HORVU7Hr1G032270
Histone H3	H32	A. thaliana		HORVU7Hr1G025330
Histone H3	H32	A. thaliana		HORVU1Hr1G080190
Histone H4	H4	A. thaliana		HORVU1Hr1G020040
Histone H4	H4	A. thaliana		HORVU1Hr1G029090
Histone H4	H4	A. thaliana		HORVU1Hr1G052030
Histone H4	H4	A. thaliana		HORVU2Hr1G097990
Histone H4	H4	A. thaliana		HORVU3Hr1G023460
Histone H4	H4	A. thaliana		HORVU6Hr1G011020
Histone H4	H4	A. thaliana		HORVU6Hr1G011710
Histone H4	H4	A. thaliana		HORVU6Hr1G029210
Histone H4	H4	A. thaliana		HORVU3Hr1G087170
Histone H4	H4	A. thaliana		HORVU1Hr1G080200
Histone H4	H4	A. thaliana		HORVU6Hr1G013530
Histone H4	H4	A. thaliana		HORVU5Hr1G087830
**DNA replication and repair**				
minichromosome maintenance protein	MCM2	O. sativa	EC:3.6.4.12	HORVU1Hr1G063700
minichromosome maintenance protein	MCM3	O. sativa	EC:3.6.4.12	HORVU1Hr1G070110
minichromosome maintenance protein	MCM6	O. sativa	EC:3.6.4.12	HORVU1Hr1G029770
minichromosome maintenance protein	MCM7	O. sativa	EC:3.6.4.12	HORVU5Hr1G028260

Some genes exhibiting the expression profile corresponding to cluster 8, successively down- and up-regulated from day 0 to day 2 and from day 2 to day 5 (Figure 3), were homologous to the rice minichromosome maintenance proteins *MCM2* (HORVU1Hr1G063700), *MCM3* (HORVU1Hr1G070110), *MCM6* (HORVU1Hr1G029770) and *MCM7* (HORVU5Hr1G028260). These are known components of a DNA helicase involved in DNA replication and cell cycle (Table 4). To date, minichromosome maintenance protein genes have not been reported in the microspore embryogenesis system. Nonetheless, evidence has been found that the *MCM2* (Ni *et al.* 2009) and *MCM7* (Springer *et al.* 2000; Holding and Springer 2002) genes were essential during the early stages of zygotic embryogenesis in *A. thaliana*. Interestingly, Ni *et al.* (2009) showed that a disruption of MCM2 gene is lethal early during embryogenesis and, by contrast, its over-expression results in an inhibition of endoreduplication. The expression profile of these genes in our system (down- then up-regulated) and the phenotype observed in our cells at day 5 (endoreduplicated cells) lead us to think that these genes might be a major regulators contributing to the induction of microspore embryogenesis.

### Concluding remarks

In barley, doubled haploid technology is widely used to develop new cultivars. Despite the wide use and economic impact of this technology, little is known about the metabolisms and pathways involved. The work presented here provides a descriptive and comprehensive overview of gene expression changes in the early stages of IMC in barley. While microspores were highly distinct phenotypically, we observed that among a gene set of ∼14k genes expressed across all three stages, a small set of ∼500 genes were uniquely expressed at a single stage. These included transcription factors associated with early embryogenesis as well as genes involved in the synthesis and response to growth regulators. Among differentially expressed genes, we saw signs of cellular responses to stress (decrease in translation, increase in GSTs, HSPs, secondary metabolism and hormone biogenesis and signal transduction). Thanks to the depth of this transcriptomic analysis, we are confident in stating that this is the most extensive characterization of the barley microspore transcriptome in IMC and provides candidate biomarkers for embryogenesis in barley.

## 

## Supplementary Material

Supplemental Material is available online at www.g3journal.org/lookup/suppl/doi:10.1534/g3.118.200208/-/DC1.
