# Supplementary material for: Differential Expression Profiling of Microspores During the Early Stages of Isolated Microspore Culture Using the Responsive Barley Cultivar Gobernadora
Source: G3 (Bethesda). 2018 Mar 12;8(5):1603–14. doi: 10.1534/g3.118.200208 (PMC5940152; doi:10.1534/g3.118.200208)
Supplement: Supplementary file 1 [file 1603TableS1.docx]

Supplementary Table 1: Gene functional annotation for genes in cluster 1

| Gene stable ID (cluster 1) | logFC D2-D0 | logFC D5-D2 | Gene function annotation |
| --- | --- | --- | --- |
| HORVU4Hr1G080390 | -3.70 | -3.54 | Acid phosphatase |
| HORVU5Hr1G076200 | -4.10 | -3.33 | Acid phosphatase |
| HORVU0Hr1G002950 | -4.28 | -2.02 | alpha/beta hydrolase fold-containing protein |
| HORVU0Hr1G010210 | -3.10 | -4.12 | Amino acid transporter |
| HORVU2Hr1G099530 | -4.02 | -2.59 | Amino acid transporter |
| HORVU2Hr1G099610 | -3.99 | -2.79 | Amino acid transporter |
| HORVU5Hr1G010470 | -3.11 | -4.23 | Amino acid transporter |
| HORVU7Hr1G032060 | -3.16 | -4.31 | Amino acid transporter |
| HORVU5Hr1G095030 | -3.61 | -3.83 | Ammonium transporter, Amt family |
| HORVU1Hr1G003200 | -2.89 | -3.99 | Beta-galactosidase |
| HORVU1Hr1G003210 | -2.46 | -3.76 | Beta-galactosidase |
| HORVU1Hr1G042180 | -2.56 | -3.11 | Flotillin |
| HORVU1Hr1G083470 | -3.92 | -3.18 | Fructokinase [EC:2.7.1.4] |
| HORVU5Hr1G114110 | -2.63 | -3.05 | Fructokinase [EC:2.7.1.4] |
| HORVU1Hr1G081940 | -4.49 | -2.50 | Glucosyl/glucoronosyl transferases |
| HORVU1Hr1G082020 | -2.82 | -3.32 | Glucosyl/glucoronosyl transferases |
| HORVU1Hr1G063560 | -4.47 | -3.67 | H+-transporting ATPase [EC:3.6.3.6] |
| HORVU2Hr1G030450 | -4.43 | -4.85 | ISP4 like protein |
| HORVU2Hr1G102890 | -4.35 | -5.08 | ISP4 like protein |
| HORVU0Hr1G021040 | -2.86 | -5.08 | Nitrate, fromate, iron dehydrogenase |
| HORVU3Hr1G110720 | -3.50 | -2.04 | Nuclear movement protein NUDC |
| HORVU4Hr1G088890 | -2.05 | -2.26 | Oligopeptide transporter |
| HORVU4Hr1G028510 | -2.87 | -2.56 | Periplasmic beta-glucosidase |
| HORVU6Hr1G032770 | -2.09 | -2.63 | Proprotein convertase subtilisin/kexin |
| HORVU1Hr1G079940 | -3.00 | -3.88 | RAG1-activating protein 1 |
| HORVU3Hr1G081290 | -3.34 | -2.47 | Ring finger domain-containing |
| HORVU3Hr1G093910 | -2.96 | -3.12 | Ring finger domain-containing |
| HORVU3Hr1G098880 | -2.43 | -2.32 | Ring finger domain-containing |
| HORVU5Hr1G092210 | -2.03 | -2.51 | S-phase kinase-associated protein 1 |
| HORVU7Hr1G113610 | -3.93 | -2.67 | Serin protease family S10 serine carboxypeptidase |
| HORVU7Hr1G011420 | -2.40 | -4.43 | Serine protease inhibitor, serpin |
| HORVU2Hr1G099040 | -3.36 | -2.20 | small subunit ribosomal protein S25e |
| HORVU2Hr1G012970 | -2.06 | -3.17 | Strictosidine synthase |
| HORVU5Hr1G043200 | -3.00 | -2.33 | Strictosidine synthase |
| HORVU6Hr1G059200 | -2.77 | -3.26 | Two-component response regulator ARR-A family |
| HORVU1Hr1G080630 | -2.32 | -2.12 | ubiquitin C |
| HORVU1Hr1G008130 | -3.57 | -2.19 | unknown |
| HORVU1Hr1G053270 | -2.47 | -2.07 | unknown |
| HORVU1Hr1G083350 | -3.31 | -2.26 | unknown |
| HORVU2Hr1G098040 | -2.83 | -2.22 | unknown |
| HORVU2Hr1G099680 | -3.67 | -2.89 | unknown |
| HORVU2Hr1G106630 | -4.73 | -4.04 | unknown |
| HORVU3Hr1G023690 | -2.80 | -3.35 | unknown |
| HORVU3Hr1G057740 | -2.13 | -2.50 | unknown |
| HORVU3Hr1G064420 | -4.27 | -3.19 | unknown |
| HORVU3Hr1G067230 | -2.43 | -4.04 | unknown |
| HORVU3Hr1G067510 | -2.98 | -3.01 | unknown |
| HORVU3Hr1G075260 | -2.76 | -3.66 | unknown |
| HORVU3Hr1G075270 | -3.33 | -3.19 | unknown |
| HORVU3Hr1G114900 | -3.27 | -3.45 | unknown |
| HORVU3Hr1G115010 | -2.07 | -3.71 | unknown |
| HORVU4Hr1G069070 | -2.55 | -2.28 | unknown |
| HORVU5Hr1G110670 | -2.01 | -2.90 | unknown |
| HORVU6Hr1G069330 | -2.33 | -2.32 | unknown |
| HORVU6Hr1G075130 | -2.33 | -2.26 | unknown |
| HORVU7Hr1G035230 | -2.11 | -2.52 | unknown |
| HORVU7Hr1G041320 | -3.11 | -3.02 | unknown |
| HORVU7Hr1G047750 | -2.41 | -3.14 | unknown |
| HORVU7Hr1G108030 | -2.89 | -3.51 | unknown |
| HORVU7Hr1G112970 | -2.55 | -2.80 | unknown |
| HORVU7Hr1G114630 | -6.44 | -4.81 | unknown |
| HORVU7Hr1G117580 | -2.57 | -2.49 | unknown |
| HORVU7Hr1G119320 | -2.16 | -2.55 | unknown |
| HORVU1Hr1G070790 | -3.26 | -3.76 | Zinc finger five domain containing protein |
