# Supplementary material for: Differential Expression Profiling of Microspores During the Early Stages of Isolated Microspore Culture Using the Responsive Barley Cultivar Gobernadora
Source: G3 (Bethesda). 2018 Mar 12;8(5):1603–14. doi: 10.1534/g3.118.200208 (PMC5940152; doi:10.1534/g3.118.200208)
Supplement: Supplementary file 2 [file 1603TableS2.docx]

Supplementary Table 2: Gene functional annotation for genes in cluster 2

| Gene stable ID (cluster 2) | logFC D2-D0 | logFC D5-D2 | Gene function annotation |
| --- | --- | --- | --- |
| HORVU3Hr1G072950 | -2,11 | -0,07 | 2-deoxyglucose-6-phosphate phosphatase 2 |
| HORVU2Hr1G005140 | -2,37 | -1,94 | 2-hydroxyacid dehydrogenase |
| HORVU5Hr1G111820 | -2,10 | 0,53 | 40S ribosomal protein S26e |
| HORVU5Hr1G006780 | -2,14 | 0,55 | 5-methyltetrahydropteroyltriglutamate--homocysteine methyltransferase |
| HORVU4Hr1G002270 | -2,12 | 0,07 | 5-methyltetrahydropteroyltriglutamate--homocysteine methyltransferase [EC:2.1.1.14] |
| HORVU1Hr1G021720 | -2,08 | 0,61 | 30S/40S ribosomal protein S4e |
| HORVU2Hr1G028510 | -2,72 | -0,94 | 30S/40S ribosomal protein S9e |
| HORVU2Hr1G029890 | -2,02 | -0,12 | 40S ribosomal protein S6e |
| HORVU2Hr1G067370 | -2,09 | 0,29 | 40S ribosomal protein S8e |
| HORVU3Hr1G111760 | -2,02 | 0,80 | 40S ribosomal protein S10e |
| HORVU1Hr1G042220 | -2,01 | 0,40 | 40S ribosomal protein S17e |
| HORVU4Hr1G077020 | -2,64 | -1,60 | 50S ribosomal protein L2 |
| HORVU5Hr1G021730 | -2,23 | 0,90 | 50S ribosomal protein L2 |
| HORVU7Hr1G073720 | -2,06 | 0,43 | 60S acid ribosomal protein |
| HORVU0Hr1G004480 | -2,26 | 0,36 | 60S ribosomal protein LP2 |
| HORVU4Hr1G019980 | -2,09 | 0,57 | 60S ribosomal protein L3 |
| HORVU4Hr1G075710 | -2,27 | 0,60 | 60S ribosomal protein L4 |
| HORVU5Hr1G092630 | -2,15 | 0,65 | 60S ribosomal protein L5e |
| HORVU6Hr1G052600 | -2,02 | 0,52 | 60S ribosomal protein L6e |
| HORVU3Hr1G084310 | -2,29 | 0,43 | 60S ribosomal protein L10Ae |
| HORVU5Hr1G052280 | -2,10 | 0,58 | 60S ribosomal protein L17 |
| HORVU1Hr1G088040 | -2,03 | 0,43 | 60S ribosomal protein L18Ae |
| HORVU4Hr1G084420 | -2,27 | 0,59 | 60S ribosomal protein L21 |
| HORVU5Hr1G075420 | -2,01 | 0,54 | 60S ribosomal protein L32 and DNA-directed RNA polymerase |
| HORVU3Hr1G062590 | -2,08 | 0,16 | 60S ribosomal protein L37Ae |
| HORVU7Hr1G081910 | -2,04 | -0,19 | 60S ribosomal protein L37Ae |
| HORVU6Hr1G091950 | -2,12 | 0,65 | aconitate hydratase 1 [EC:4.2.1.3] |
| HORVU5Hr1G117900 | -2,18 | 0,64 | actin, other eukaryote |
| HORVU5Hr1G018080 | -2,15 | -0,02 | adenine phosphoribosyltransferase [EC:2.4.2.7] |
| HORVU5Hr1G037200 | -2,07 | -1,59 | aldehyde dehydrogenase (NAD+) [EC:1.2.1.3] |
| HORVU2Hr1G125630 | -2,74 | -1,24 | alkylated DNA repair protein alkB homolog 5 |
| HORVU1Hr1G048430 | -2,18 | 0,71 | alpha,alpha-trehalase [EC:3.2.1.28] |
| HORVU3Hr1G082280 | -3,00 | -0,13 | amidophosphoribosyltransferase [EC:2.4.2.14] |
| HORVU5Hr1G019110 | -3,31 | -0,60 | amino acid transporter |
| HORVU7Hr1G038330 | -3,25 | 1,67 | amino acid transporter |
| HORVU7Hr1G074640 | -3,02 | -0,14 | amino acid transporter |
| HORVU1Hr1G092960 | -2,76 | 1,13 | amino acid transporter |
| HORVU2Hr1G088420 | -4,88 | 0,31 | ammonium transporter |
| HORVU2Hr1G088380 | -4,83 | 0,26 | ammonium transporter |
| HORVU5Hr1G084490 | -2,77 | -1,64 | aspartyl proteases |
| HORVU4Hr1G082240 | -2,15 | 1,31 | band 7 protein-related |
| HORVU5Hr1G065470 | -3,15 | 0,01 | beta catenin-related armadillo repeat-containing |
| HORVU2Hr1G113270 | -4,07 | 1,26 | beta-aspartyl-peptidase (threonine type) [EC:3.4.19.5] |
| HORVU3Hr1G089520 | -2,05 | 1,66 | beta-glucosidase [EC:3.2.1.21] |
| HORVU6Hr1G087460 | -2,06 | -1,41 | betaine-aldehyde dehydrogenase [EC:1.2.1.8] |
| HORVU2Hr1G088840 | -2,76 | 1,78 | BRCA1-associated RING domain protein 1 [EC:6.3.2.19] |
| HORVU5Hr1G076190 | -2,64 | -0,32 | calcium-activated chloride channel regulator |
| HORVU3Hr1G106950 | -2,96 | 0,52 | calcium-binding protein CML |
| HORVU5Hr1G043460 | -2,08 | -0,06 | calcium-binding protein CML |
| HORVU0Hr1G001270 | -2,33 | 0,60 | calmodulin |
| HORVU4Hr1G082040 | -2,70 | 0,39 | catalase [EC:1.11.1.6] |
| HORVU3Hr1G084800 | -2,09 | 0,46 | cell division control protein 6 |
| HORVU1Hr1G080160 | -3,75 | 1,27 | cell division cycle 2-like [EC:2.7.11.22]; cell division protein kinase |
| HORVU6Hr1G012510 | -2,02 | 0,23 | cellular nucleic acid-binding protein |
| HORVU2Hr1G027370 | -2,34 | 0,50 | centromere/microtubule binding protein CBF5 |
| HORVU2Hr1G027400 | -2,19 | 0,44 | centromere/microtubule binding protein CBF5 |
| HORVU1Hr1G072810 | -2,26 | -0,94 | circadian protein CLOCK/ARNT/BMAL/PAS |
| HORVU6Hr1G087330 | -2,80 | 0,49 | clathrin assembly protein |
| HORVU1Hr1G064210 | -2,17 | 1,44 | cold shock domain containing proteins |
| HORVU3Hr1G092680 | -4,39 | 1,32 | coniferyl-aldehyde dehydrogenase [EC:1.2.1.68] |
| HORVU7Hr1G048620 | -2,06 | -1,46 | copine |
| HORVU2Hr1G077670 | -2,49 | 0,95 | CPG binding protein |
| HORVU4Hr1G013320 | -2,52 | 1,21 | cyclin A |
| HORVU5Hr1G045040 | -2,19 | 0,83 | cytochrome B5 |
| HORVU2Hr1G058800 | -2,32 | 1,56 | cytosine-specific methyltransferase |
| HORVU3Hr1G074280 | -2,09 | 1,22 | dCMP deaminase [EC:3.5.4.12] |
| HORVU1Hr1G091090 | -2,10 | -0,03 | delta14-sterol reductase [EC:1.3.1.70] |
| HORVU4Hr1G005810 | -2,03 | 1,17 | denticleless |
| HORVU0Hr1G039040 | -2,34 | 1,17 | DNA (cytosine-5-)-methyltransferase [EC:2.1.1.37] |
| HORVU7Hr1G077400 | -2,73 | 0,38 | DNA cross-link repair protein PSO2/SNM1-related |
| HORVU7Hr1G102300 | -2,03 | 1,54 | DNA polymerase 2 alpha 70 kDa |
| HORVU2Hr1G069780 | -2,12 | 1,90 | DNA primase large subunit [EC:2.7.7.-] |
| HORVU2Hr1G014660 | -2,16 | 0,26 | DNA repair and recombination protein RAD54 [EC:3.6.4.-] |
| HORVU5Hr1G087670 | -2,07 | 1,11 | DNA repair dead helicase RAD3/XP-D |
| HORVU2Hr1G031130 | -2,30 | 1,30 | eukaryotic translation initiation factor 2C |
| HORVU0Hr1G012490 | -2,03 | 1,49 | eukaryotic translation initiation factor 2C |
| HORVU7Hr1G082480 | -2,16 | 1,42 | exonuclease 1 [EC:3.1.-.-] |
| HORVU6Hr1G060570 | -3,52 | -0,73 | FAS-associated protein |
| HORVU5Hr1G078930 | -7,16 | -0,57 | flotillin |
| HORVU6Hr1G033350 | -2,34 | 0,17 | fumarylacetoacetase [EC:3.7.1.2] |
| HORVU2Hr1G089710 | -2,28 | 0,98 | FXNA-related |
| HORVU6Hr1G074030 | -2,43 | -1,16 | glutamine synthetase [EC:6.3.1.2] |
| HORVU2Hr1G032670 | -3,42 | -1,83 | glycerophosphoryl diester phosphodiesterase [EC:3.1.4.46] |
| HORVU2Hr1G110110 | -2,35 | 0,89 | glycine hydroxymethyltransferase [EC:2.1.2.1] |
| HORVU0Hr1G005260 | -2,49 | -0,81 | glycosyltransferase 8 |
| HORVU2Hr1G091280 | -2,04 | -1,73 | glycosyltransferase 8 |
| HORVU1Hr1G083710 | -2,21 | -0,04 | guanine nucleotide-binding protein subunit beta-2-like 1 protein |
| HORVU6Hr1G081460 | -2,48 | 0,49 | heat shock 70kDa protein 1/8 |
| HORVU5Hr1G021300 | -2,01 | 0,69 | heat shock 70kDa protein 1/8 |
| HORVU5Hr1G072420 | -2,17 | 0,68 | heat shock protein 90kDa |
| HORVU6Hr1G017910 | -2,46 | 1,80 | heat shock transcription factor |
| HORVU6Hr1G011950 | -2,06 | 1,90 | histone acetyltransferase |
| HORVU5Hr1G041720 | -2,19 | 1,84 | histone acetyltransferase 1 [EC:2.3.1.48] |
| HORVU6Hr1G092280 | -2,42 | 1,57 | histone H2A |
| HORVU3Hr1G116550 | -2,19 | 1,33 | histone H2A |
| HORVU4Hr1G058940 | -2,18 | 1,90 | histone H2A |
| HORVU4Hr1G008800 | -2,11 | 1,13 | histone H2A |
| HORVU3Hr1G086610 | -2,10 | 1,30 | histone H2B |
| HORVU1Hr1G020050 | -2,55 | 1,54 | histone H3 |
| HORVU1Hr1G017830 | -2,30 | 1,98 | histone H4 |
| HORVU5Hr1G084160 | -2,71 | 1,32 | histone-binding protein RBBP4 |
| HORVU7Hr1G121630 | -2,05 | -0,29 | hypothetical protein F36F12.3 |
| HORVU1Hr1G076570 | -6,35 | -1,56 | interleukin-1 receptor-associated kinase 1 [EC:2.7.11.1] |
| HORVU5Hr1G079160 | -2,24 | 1,23 | kelch-related |
| HORVU3Hr1G097530 | -9,74 | -0,20 | KUP system potassium uptake protein |
| HORVU7Hr1G029650 | -2,02 | 0,64 | LIM domain-binding protein |
| HORVU2Hr1G085880 | -2,22 | 0,76 | lupus La protein |
| HORVU7Hr1G056460 | -2,16 | 1,07 | lupus La protein |
| HORVU0Hr1G021970 | -2,33 | 0,44 | methyltransferase |
| HORVU5Hr1G110180 | -3,09 | -1,28 | MFS transporter, PHS family, inorganic phosphate transporter |
| HORVU5Hr1G110220 | -2,97 | -0,93 | MFS transporter, PHS family, inorganic phosphate transporter |
| HORVU5Hr1G084050 | -2,05 | 0,77 | minichromosome maintenance protein 10 |
| HORVU2Hr1G096250 | -8,34 | 1,05 | multi-copper oxidase |
| HORVU7Hr1G105620 | -3,44 | -1,13 | multi-copper oxidase |
| HORVU7Hr1G027270 | -2,09 | 0,22 | multidrug resistance protein, MATE family |
| HORVU3Hr1G079490 | -2,45 | 1,59 | myb proto-oncogene protein, plant |
| HORVU7Hr1G090120 | -2,21 | -1,38 | myb-like DNA-binding protein |
| HORVU1Hr1G072140 | -2,64 | -0,50 | NADPH oxidase |
| HORVU4Hr1G050160 | -2,30 | 0,77 | nardilysin [EC:3.4.24.61]; metalloprotease |
| HORVU3Hr1G056170 | -2,30 | 1,29 | nuclear LIM interactor-interacting factor |
| HORVU2Hr1G022140 | -2,34 | 1,36 | nucleolar protein 56 |
| HORVU1Hr1G083960 | -2,13 | 0,88 | nucleolar protein 56 |
| HORVU7Hr1G068630 | -2,47 | 0,49 | nucleolin |
| HORVU7Hr1G071600 | -2,64 | -1,10 | oligopeptide transporter |
| HORVU5Hr1G096870 | -2,19 | 0,73 | origin recognition complex subunit 5 |
| HORVU7Hr1G097570 | -2,07 | 0,93 | para-aminobenzoate synthetase [EC:2.6.1.85] |
| HORVU3Hr1G075970 | -2,76 | 0,80 | pectate lyase [EC:4.2.2.2] |
| HORVU4Hr1G024280 | -7,40 | 0,86 | phospholipase |
| HORVU4Hr1G002500 | -2,62 | 0,60 | pre-rRNA-processing protein TSR2 |
| HORVU3Hr1G019670 | -2,32 | 0,82 | profilin |
| HORVU1Hr1G061760 | -2,70 | 1,76 | proliferating cell nuclear antigen |
| HORVU5Hr1G074040 | -2,64 | 1,79 | proliferating cell nuclear antigen |
| HORVU1Hr1G065140 | -2,43 | 1,57 | proliferating cell nuclear antigen |
| HORVU7Hr1G062710 | -2,14 | 1,34 | proliferating cell nuclear antigen |
| HORVU5Hr1G087880 | -2,75 | 0,54 | putative transport protein |
| HORVU1Hr1G047820 | -2,30 | 1,02 | pyridoxine 4-dehydrogenase [EC:1.1.1.65] |
| HORVU7Hr1G094270 | -2,28 | 1,27 | QRI2 protein |
| HORVU3Hr1G089630 | -2,58 | 0,74 | RAS GTPase-activating protein-binding protein |
| HORVU7Hr1G074870 | -2,34 | 0,81 | replication factor C DNA polymerase III famma-TAU subunit |
| HORVU1Hr1G082010 | -2,54 | 0,68 | reticulon |
| HORVU6Hr1G091860 | -2,43 | 0,90 | rRNA 2'-O-methyltransferase fibrillarin [EC:2.1.1.-] |
| HORVU5Hr1G064040 | -2,15 | -1,43 | S-adenosylmethionine decarboxylase [EC:4.1.1.50] |
| HORVU5Hr1G084140 | -2,10 | 0,35 | S-phase kinase-associated protein 1 |
| HORVU2Hr1G100360 | -3,77 | 0,95 | SAUR family protein |
| HORVU1Hr1G047390 | -2,01 | 1,75 | SAUR family protein |
| HORVU2Hr1G102410 | -2,05 | 1,82 | senataxin [EC:3.6.4.-] |
| HORVU7Hr1G059850 | -7,25 | 1,98 | serine carboxypeptidase 1 [EC:3.4.16.-] |
| HORVU7Hr1G094190 | -9,34 | -0,15 | serine protease family S10 serine carboxypeptidase |
| HORVU3Hr1G033560 | -7,52 | -0,26 | serine protease family S10 serine carboxypeptidase |
| HORVU4Hr1G016730 | -2,03 | 0,73 | serine protease family S10 serine carboxypeptidase |
| HORVU7Hr1G038950 | -2,03 | 1,06 | serine protease family S1C HTRA-related |
| HORVU3Hr1G020090 | -3,04 | 0,93 | serine protease inhibitor, serpin |
| HORVU3Hr1G026870 | -2,35 | -0,14 | serine-threonine protein kinase |
| HORVU0Hr1G015980 | -2,33 | -1,33 | serine-threonine protein kinase |
| HORVU2Hr1G016750 | -2,75 | -0,70 | serine-threonine protein kinase |
| HORVU4Hr1G083080 | -2,00 | 1,86 | serine-threonine protein kinase |
| HORVU3Hr1G004890 | -2,14 | 0,38 | seven in absentia homolog |
| HORVU5Hr1G120280 | -2,28 | 0,06 | sister chromatid cohesion protein DCC1 |
| HORVU2Hr1G021020 | -2,58 | 1,07 | sodium/hydrogen exchanger |
| HORVU6Hr1G070780 | -2,50 | 1,35 | solute carrier family 25 (mitochondrial carrier; adenine nucleotide translocator) |
| HORVU7Hr1G114550 | -2,27 | -1,61 | speckle-type POZ protein |
| HORVU3Hr1G065520 | -6,96 | -0,79 | strictosidine synthase |
| HORVU6Hr1G072930 | -4,70 | 1,81 | sulfate transporter |
| HORVU0Hr1G001880 | -3,80 | 1,87 | SWI/SNF-related matrix-associated actin-dependant regulator of chromatin |
| HORVU3Hr1G087250 | -3,34 | 0,38 | TBC1 domain family member; GTPase-activating protein |
| HORVU1Hr1G071360 | -2,01 | -0,29 | TBC1 domain family member; GTPase-activating protein |
| HORVU4Hr1G004280 | -2,29 | 1,01 | tesmin/TSO1-RELATED |
| HORVU7Hr1G064820 | -2,22 | 1,02 | testis-specific Y-encoded protein |
| HORVU7Hr1G064790 | -2,10 | 0,91 | testis-specific Y-encoded protein |
| HORVU4Hr1G074650 | -2,64 | 1,31 | timeless-interacting protein |
| HORVU4Hr1G003650 | -2,35 | -1,17 | TPR repeat |
| HORVU1Hr1G066200 | -2,27 | -0,28 | transcription initiation factor TFIIB |
| HORVU1Hr1G073940 | -5,36 | 0,37 | transcriptional adaptor 2 (ADA2) |
| HORVU4Hr1G072010 | -2,13 | 0,06 | transitional endoplasmic reticulum ATPase |
| HORVU4Hr1G060330 | -2,58 | 1,82 | tropine dehydrogenase [EC:1.1.1.206] |
| HORVU5Hr1G123530 | -2,52 | 0,28 | troponin C-akin-1 protein |
| HORVU1Hr1G023660 | -2,03 | 0,64 | ubiquitin |
| HORVU3Hr1G110970 | -2,36 | -0,88 | ubiquitin C |
| HORVU3Hr1G113820 | -2,10 | 0,55 | UDP-apiose/xylose synthase |
| HORVU5Hr1G080860 | -3,09 | 1,28 | UDP-glucose 4-epimerase [EC:5.1.3.2] |
| HORVU2Hr1G105360 | -8,64 | 0,89 | unknown |
| HORVU5Hr1G076000 | -8,63 | -1,06 | unknown |
| HORVU2Hr1G034310 | -7,66 | -0,28 | unknown |
| HORVU1Hr1G085230 | -7,62 | -0,33 | unknown |
| HORVU2Hr1G092170 | -6,10 | -1,40 | unknown |
| HORVU2Hr1G089170 | -5,28 | -0,81 | unknown |
| HORVU0Hr1G013830 | -4,61 | -1,54 | unknown |
| HORVU7Hr1G028830 | -4,52 | -1,46 | unknown |
| HORVU5Hr1G078490 | -3,99 | -1,72 | unknown |
| HORVU3Hr1G049860 | -3,96 | -1,45 | unknown |
| HORVU1Hr1G052040 | -3,96 | 1,68 | unknown |
| HORVU3Hr1G024210 | -3,89 | 0,81 | unknown |
| HORVU4Hr1G010490 | -3,86 | -0,32 | unknown |
| HORVU3Hr1G036960 | -3,86 | -0,38 | unknown |
| HORVU2Hr1G116420 | -3,82 | 0,78 | unknown |
| HORVU6Hr1G024700 | -3,75 | -1,13 | unknown |
| HORVU2Hr1G097170 | -3,70 | -1,11 | unknown |
| HORVU2Hr1G096100 | -3,41 | 1,50 | unknown |
| HORVU5Hr1G041930 | -3,28 | 1,40 | unknown |
| HORVU3Hr1G066880 | -3,10 | -0,21 | unknown |
| HORVU3Hr1G019760 | -3,06 | -0,48 | unknown |
| HORVU2Hr1G087070 | -3,04 | -0,96 | unknown |
| HORVU3Hr1G109880 | -3,04 | -0,62 | unknown |
| HORVU1Hr1G043620 | -3,00 | -1,43 | unknown |
| HORVU0Hr1G040420 | -2,98 | -0,69 | unknown |
| EPlHVUG00000039832 | -2,94 | -0,85 | unknown |
| HORVU1Hr1G083340 | -2,89 | 0,00 | unknown |
| HORVU7Hr1G035050 | -2,89 | 0,22 | unknown |
| HORVU3Hr1G062730 | -2,79 | 0,53 | unknown |
| HORVU3Hr1G029310 | -2,78 | -0,10 | unknown |
| HORVU6Hr1G084390 | -2,78 | 1,77 | unknown |
| HORVU2Hr1G039960 | -2,74 | 1,83 | unknown |
| HORVU3Hr1G092400 | -2,72 | -1,05 | unknown |
| HORVU2Hr1G090380 | -2,69 | -1,37 | unknown |
| HORVU3Hr1G106710 | -2,65 | -1,97 | unknown |
| HORVU7Hr1G112960 | -2,59 | -0,41 | unknown |
| HORVU2Hr1G018070 | -2,59 | -1,55 | unknown |
| HORVU7Hr1G000260 | -2,58 | -1,88 | unknown |
| HORVU1Hr1G095250 | -2,55 | -0,02 | unknown |
| HORVU7Hr1G119190 | -2,53 | -1,85 | unknown |
| HORVU5Hr1G088360 | -2,53 | 0,94 | unknown |
| HORVU5Hr1G091810 | -2,50 | 1,71 | unknown |
| HORVU2Hr1G079780 | -2,48 | 0,55 | unknown |
| HORVU1Hr1G052010 | -2,46 | 0,23 | unknown |
| HORVU3Hr1G117270 | -2,43 | 0,43 | unknown |
| HORVU3Hr1G096450 | -2,39 | 1,44 | unknown |
| HORVU1Hr1G026400 | -2,31 | 1,04 | unknown |
| HORVU5Hr1G079730 | -2,29 | 1,94 | unknown |
| HORVU2Hr1G097760 | -2,29 | -0,85 | unknown |
| HORVU5Hr1G123200 | -2,28 | -0,54 | unknown |
| HORVU3Hr1G076370 | -2,27 | 0,91 | unknown |
| HORVU2Hr1G107140 | -2,27 | -1,78 | unknown |
| HORVU5Hr1G080170 | -2,27 | 0,94 | unknown |
| HORVU3Hr1G089370 | -2,26 | -1,11 | unknown |
| HORVU5Hr1G101500 | -2,25 | 0,84 | unknown |
| HORVU3Hr1G069410 | -2,23 | -0,47 | unknown |
| HORVU1Hr1G095140 | -2,22 | 0,89 | unknown |
| HORVU5Hr1G080580 | -2,19 | 0,58 | unknown |
| HORVU2Hr1G105700 | -2,18 | 0,50 | unknown |
| HORVU6Hr1G075150 | -2,17 | -0,85 | unknown |
| HORVU2Hr1G019990 | -2,16 | -1,76 | unknown |
| HORVU5Hr1G054380 | -2,10 | 1,30 | unknown |
| HORVU4Hr1G065080 | -2,09 | 0,28 | unknown |
| HORVU3Hr1G070150 | -2,09 | -0,95 | unknown |
| HORVU5Hr1G110530 | -2,09 | -0,38 | unknown |
| HORVU4Hr1G017860 | -2,08 | -1,19 | unknown |
| HORVU6Hr1G084810 | -2,07 | -0,46 | unknown |
| HORVU2Hr1G082340 | -2,07 | 1,03 | unknown |
| HORVU3Hr1G014530 | -2,06 | -0,96 | unknown |
| HORVU7Hr1G119250 | -2,06 | 0,88 | unknown |
| HORVU7Hr1G114250 | -2,05 | 1,52 | unknown |
| HORVU3Hr1G001360 | -2,02 | 0,41 | unknown |
| HORVU4Hr1G021110 | -2,02 | -0,64 | unknown |
| HORVU2Hr1G106480 | -2,02 | 0,22 | unknown |
| HORVU4Hr1G033720 | -2,02 | 1,60 | unknown |
| HORVU2Hr1G105930 | -2,01 | 1,95 | unknown |
| HORVU5Hr1G110760 | -2,69 | -0,59 | unknown |
| HORVU5Hr1G000700 | -2,57 | 0,88 | vesicle-associated membrane protein 7 |
| HORVU5Hr1G081900 | -2,30 | -0,36 | WNK lysine deficient protein kinase [EC:2.7.11.1] |
| HORVU2Hr1G025980 | -2,01 | -0,52 | xylulokinase [EC:2.7.1.17]; sugar kinase |
| HORVU3Hr1G062620 | -2,15 | 0,95 | YTH (YT521-B Homology) domain-containing |
| HORVU7Hr1G117770 | -11,10 | 0,46 | zinc finger five domain containing protein |
| HORVU1Hr1G036440 | -2,38 | 0,90 | zinc finger five domain containing protein |
