# Supplementary material for: Differential Expression Profiling of Microspores During the Early Stages of Isolated Microspore Culture Using the Responsive Barley Cultivar Gobernadora
Source: G3 (Bethesda). 2018 Mar 12;8(5):1603–14. doi: 10.1534/g3.118.200208 (PMC5940152; doi:10.1534/g3.118.200208)
Supplement: Supplementary file 3 [file 1603TableS3.docx]

Supplementary Table 3: Gene functional annotation for genes in cluster 3

| Gene stable ID (cluster 3) | logFC D2-D0 | logFC D5-D2 | Gene function annotation |
| --- | --- | --- | --- |
| HORVU2Hr1G077120 | 0,65 | -2,69 | 1,4-alpha-glucan branching enzyme [EC:2.4.1.18]; alpha-amylase |
| HORVU2Hr1G099580 | 1,88 | -2,24 | 26S proteasome regulatory subunit N12 |
| HORVU3Hr1G069990 | -1,77 | -2,97 | 3-5 exonuclease |
| HORVU2Hr1G016730 | 0,35 | -2,27 | 5'-AMP-activated protein kinase, regulatory beta subunit |
| HORVU2Hr1G005420 | -0,06 | -2,83 | acetyl-CoA C-acyltransferase |
| HORVU5Hr1G055570 | 0,07 | -3,22 | acid phosphatase |
| HORVU7Hr1G045470 | 1,49 | -2,45 | aconitase |
| HORVU3Hr1G091280 | -0,70 | -3,15 | aldose 1-epimerase [EC:5.1.3.3] |
| HORVU7Hr1G027860 | 1,73 | -2,38 | alpha-amylase |
| HORVU6Hr1G075240 | -1,45 | -4,11 | alpha-galactosidase/alpha-N-acetylgalactosaminidase |
| HORVU2Hr1G017080 | 0,65 | -3,05 | alpha/beta hydrolase fold-containing protein |
| HORVU2Hr1G029840 | 1,85 | -2,67 | alpha/beta hydrolase fold-containing protein |
| HORVU5Hr1G103940 | -1,01 | -2,50 | AN1-type zing finger protein |
| HORVU2Hr1G036250 | 0,06 | -2,23 | AN1-type zing finger protein |
| HORVU4Hr1G086960 | 0,05 | -2,19 | AP endonuclease |
| HORVU7Hr1G016870 | -1,53 | -3,26 | ATP-binding cassette transporter |
| HORVU3Hr1G105140 | -0,33 | -2,75 | ATP-binding cassette transporter |
| HORVU4Hr1G087670 | 1,25 | -4,70 | ATP-binding cassette transporter (ABCA) |
| HORVU5Hr1G123460 | 1,53 | -2,87 | ATP-dependent CLP protease |
| HORVU4Hr1G014120 | -0,81 | -2,17 | ATP-dependent Clp protease ATP-binding subunit ClpC |
| HORVU5Hr1G041530 | 1,67 | -2,98 | ATP-dependent CLP protease (CEREBLON) |
| HORVU1Hr1G093600 | 0,42 | -4,16 | BCS1 AAA-type ATPase |
| HORVU7Hr1G105580 | 1,10 | -2,23 | BEIGE/BEACH-related |
| HORVU7Hr1G105700 | 1,10 | -2,74 | BEIGE/BEACH-related |
| HORVU7Hr1G001070 | -1,19 | -2,33 | beta-fructofuranosidase [EC:3.2.1.26] |
| HORVU4Hr1G011000 | 0,43 | -2,16 | beta-fructofuranosidase [EC:3.2.1.26] |
| HORVU2Hr1G082150 | 1,83 | -2,47 | beta-glucosidase [EC:3.2.1.21] |
| HORVU2Hr1G119350 | 1,93 | -2,77 | beta-glucosidase [EC:3.2.1.21] |
| HORVU6Hr1G034630 | 1,98 | -3,10 | beta-mannan synthase [EC:2.4.1.32] |
| HORVU3Hr1G102890 | -0,20 | -2,51 | Ca2+-transporting ATPase [EC:3.6.3.8] |
| HORVU3Hr1G088310 | 1,04 | -2,11 | calcium binding protein 39 |
| HORVU1Hr1G092690 | 0,03 | -3,65 | calcium-activated chloride channel regulator |
| HORVU3Hr1G052770 | 1,25 | -3,24 | calcium-binding protein CML |
| HORVU3Hr1G079040 | 1,86 | -3,17 | calcium-dependent protein kinase [EC:2.7.11.1]; serine/threonine-protein kinase |
| HORVU5Hr1G103830 | 1,87 | -3,04 | calcium-dependent protein kinase [EC:2.7.11.1]; serine/threonine-protein kinase |
| HORVU4Hr1G026290 | -1,43 | -2,48 | calmodulin |
| HORVU6Hr1G018420 | 0,23 | -2,29 | calnexin |
| HORVU5Hr1G116590 | -0,54 | -2,80 | calreticulin |
| HORVU1Hr1G081400 | 1,45 | -2,92 | cell cycle control protein 50 |
| HORVU4Hr1G000920 | -0,64 | -2,21 | chaperone ATP11 |
| HORVU3Hr1G094820 | 1,71 | -2,37 | chitinase |
| HORVU3Hr1G081920 | -0,18 | -2,34 | chloride channel 7 |
| HORVU1Hr1G080520 | -1,23 | -2,24 | choline/ethanolamine kinase [EC:2.7.1.32 2.7.1.82] |
| HORVU5Hr1G000970 | -0,68 | -2,56 | coiled-coil-helix-coiled-coil-helix domain containing 2/NUR77 |
| HORVU2Hr1G022680 | 0,98 | -2,61 | complex I intermediated-associated protein 30 |
| HORVU5Hr1G125030 | -0,55 | -2,71 | copper transport protein ATOX1-related |
| HORVU3Hr1G072940 | 1,14 | -4,19 | copper transport protein ATOX1-related |
| HORVU0Hr1G022680 | -1,53 | -2,55 | CTX-related type I transmembrane protein |
| HORVU5Hr1G065950 | 0,40 | -2,21 | cyclic nucleotide gated channel, other eukaryote |
| HORVU5Hr1G092890 | 1,08 | -3,88 | cyclic nucleotide gated channel, other eukaryote |
| HORVU1Hr1G018730 | 1,67 | -2,48 | cyclic nucleotide gated channel, other eukaryote |
| HORVU7Hr1G035770 | -1,23 | -3,10 | cyclin-dependent kinase inhibitor I |
| HORVU3Hr1G090840 | 1,58 | -2,03 | cytochrome c |
| HORVU2Hr1G015430 | -0,68 | -2,42 | D-alanine—D-alanine ligase |
| HORVU3Hr1G030390 | 1,45 | -4,43 | dimethylaniline monooxygenase |
| HORVU2Hr1G040130 | 1,54 | -3,07 | disulfide oxidoreductase |
| HORVU3Hr1G098970 | 1,42 | -2,61 | DNA (cytosine-5-)-methyltransferase 3-related |
| HORVU5Hr1G116070 | 0,83 | -3,21 | DNAJ homolog subfamily C member |
| HORVU4Hr1G056070 | 1,39 | -2,42 | DNAJ homolog subfamily C member |
| HORVU7Hr1G079030 | 0,42 | -2,26 | Dolichyl-phosphate-mannose-protein mannosyltransferase |
| HORVU7Hr1G101130 | -1,46 | -3,15 | DUF292 |
| HORVU2Hr1G093390 | 1,76 | -2,65 | engulfment and cell motility |
| HORVU2Hr1G041590 | -1,84 | -2,53 | equilibrative nucleoside transporter |
| HORVU4Hr1G034860 | 1,32 | -2,26 | ethanolaminephosphotransferase |
| HORVU2Hr1G017640 | -0,22 | -2,47 | eukaryotic translation initiation factor |
| HORVU4Hr1G021040 | 1,46 | -2,24 | exocyst complex component 7 |
| HORVU4Hr1G002040 | -1,17 | -2,61 | exotosin (heparan sulfate glycosyltransferase)-related |
| HORVU6Hr1G060490 | 1,85 | -2,33 | extended synaptotagmin-related |
| HORVU5Hr1G097030 | 1,56 | -3,65 | extensin, proline-rich protein |
| HORVU5Hr1G100890 | -0,13 | -2,37 | fatty acid hydrolase |
| HORVU7Hr1G045950 | 0,14 | -2,85 | fimbrin/plastin |
| HORVU4Hr1G084520 | 0,82 | -2,75 | fringe-related |
| HORVU6Hr1G030530 | -0,08 | -2,20 | glucose-6-phosphate 1-epimerase [EC:5.1.3.15]; apospory-associated protein C-related |
| HORVU5Hr1G094450 | -0,66 | -2,92 | glucosyl/glucuronosyl transferases |
| HORVU7Hr1G085200 | 1,41 | -2,65 | glucosyl/glucuronosyl transferases |
| HORVU3Hr1G079720 | 1,21 | -2,18 | glycosyl hydrolase |
| HORVU4Hr1G060440 | 1,95 | -2,32 | glycosyltransferase; digalactosyldiacylglycerol synthase [EC:2.4.1.241] |
| HORVU7Hr1G012380 | 1,32 | -2,96 | glycosyltransferase; granule-bound starch synthase [EC:2.4.1.242] |
| HORVU3Hr1G093830 | 0,18 | -2,46 | glycosyltransferase; sucrose-phosphate synthase [EC:2.4.1.14] |
| HORVU3Hr1G068070 | 1,75 | -3,12 | guanyl-nucleotide exchange factor |
| HORVU7Hr1G114340 | -1,59 | -4,10 | H+-transporting ATPase [EC:3.6.3.6] |
| HORVU4Hr1G077430 | 1,96 | -2,45 | H+-transporting ATPase [EC:3.6.3.6] |
| HORVU0Hr1G016920 | 0,99 | -2,10 | heat shock protein 70; hypoxia up-regulated 1 |
| HORVU2Hr1G034120 | -1,49 | -3,39 | heparanase-related |
| HORVU2Hr1G034090 | -1,39 | -3,89 | heparanase-related; heparanase 1 [EC:3.2.1.-] |
| HORVU6Hr1G007470 | -0,88 | -2,05 | histone H3 |
| HORVU7Hr1G115920 | 1,49 | -2,27 | homogentisate solanesyltransferase |
| HORVU5Hr1G082440 | -0,71 | -2,48 | HVA22-like proteins |
| HORVU3Hr1G014210 | -0,01 | -4,45 | Hypothetical protein B0507.2 |
| HORVU2Hr1G012850 | 1,37 | -2,45 | IAA-amino acid hydrolase [EC:3.5.1.-] |
| HORVU7Hr1G100520 | -1,07 | -2,07 | inhibitor of apoptosis |
| HORVU7Hr1G037300 | 0,48 | -4,01 | inositol 5-phosphatase |
| HORVU1Hr1G002820 | 1,27 | -3,54 | inositol 5-phosphatase |
| HORVU3Hr1G079850 | 1,64 | -3,82 | inositol 5-phosphatase |
| HORVU6Hr1G060750 | 0,72 | -2,16 | KDEL (LYS-ASP-GLU-LEU) containing-related |
| HORVU7Hr1G056230 | -0,15 | -2,82 | KELCH-related |
| HORVU3Hr1G104870 | 0,38 | -2,22 | KUP system potassium uptake protein; osmotic stress potassium transporter |
| HORVU5Hr1G072680 | -0,47 | -3,24 | large subunit ribosomal protein L40e |
| HORVU7Hr1G091910 | 1,67 | -2,80 | large subunit ribosomal protein LP2 |
| HORVU2Hr1G007610 | 1,01 | -3,23 | leucine-rich repeat-containing protein |
| HORVU5Hr1G125340 | 1,15 | -2,26 | leucine-rich repeat-containing protein |
| HORVU5Hr1G125350 | 1,25 | -2,50 | leucine-rich repeat-containing protein |
| HORVU5Hr1G054000 | 0,12 | -2,02 | light-harvesting complex II chlorophyll a/b binding protein 7 |
| HORVU3Hr1G064450 | 0,95 | -2,62 | lipid phosphate phosphatase |
| HORVU3Hr1G062350 | 0,89 | -2,01 | lung seven transmembrane receptor |
| HORVU7Hr1G092250 | 1,61 | -2,12 | lysosomal acid lipase/cholesteryl ester hydrolase [EC:3.1.1.13] |
| HORVU4Hr1G021620 | -0,34 | -2,95 | lysosomal Pro-X carboxypeptidase [EC:3.4.16.2]; protease S28 PRO-X carboxypeptidase-related |
| HORVU4Hr1G032440 | 0,85 | -2,41 | MADS box protein |
| HORVU7Hr1G074050 | -0,22 | -2,63 | magnesium and cobalt transport protein/mirochondrial import membrane translocase subunit TIM8 |
| HORVU7Hr1G078330 | 0,89 | -2,78 | manganese transport protein |
| HORVU1Hr1G091110 | 1,18 | -3,31 | member of 'GDXG' family of lipolytic enzymes |
| HORVU1Hr1G073050 | -0,56 | -2,34 | membrane associated ring finger |
| HORVU7Hr1G074970 | -1,04 | -2,31 | methyltransferase |
| HORVU3Hr1G088050 | -0,27 | -2,39 | methyltransferase |
| HORVU4Hr1G033650 | 0,07 | -2,88 | methyltransferase |
| HORVU3Hr1G117540 | 1,25 | -2,53 | methyltransferase (uncharacterized) |
| HORVU4Hr1G080730 | -0,47 | -4,27 | MFS transporter, PHS family, inorganic phosphate transporter |
| HORVU3Hr1G039040 | 0,04 | -2,35 | mitochondrial import receptor subunit TOM40 |
| HORVU2Hr1G062700 | -0,48 | -2,84 | mitogen-activated kinase |
| HORVU6Hr1G029780 | -0,28 | -2,24 | mitogen-activated kinase; oxidative-stress responsive protein 1 [EC:2.7.11.1] |
| HORVU5Hr1G042400 | 1,33 | -2,22 | MKIAA1688 protein |
| HORVU3Hr1G097070 | 0,77 | -2,16 | monolysocardiolipin acyltransferase [EC:2.3.1.-] |
| HORVU3Hr1G080360 | 1,60 | -3,25 | multi-copped oxidase |
| HORVU1Hr1G068960 | 1,90 | -2,92 | multidrug resistance protein |
| HORVU6Hr1G091850 | -1,86 | -3,37 | multidrug resistance protein, MATE family |
| HORVU4Hr1G087590 | 1,13 | -3,15 | MYC |
| HORVU1Hr1G080240 | 0,85 | -2,00 | myosin V |
| HORVU5Hr1G125430 | 1,83 | -2,62 | myosin V |
| HORVU5Hr1G080140 | -1,03 | -2,75 | myrosinase binding protein-related |
| HORVU7Hr1G105060 | -1,35 | -2,31 | NEP1/MRA1 |
| HORVU5Hr1G089450 | 0,04 | -3,03 | nucleolar complex 2 and RAD4-related |
| HORVU3Hr1G111060 | 0,77 | -3,80 | nucleolar complex 2 and RAD4-related |
| HORVU4Hr1G090780 | 0,15 | -2,51 | nucleoporin-related |
| HORVU7Hr1G012680 | 1,35 | -2,80 | O-methyltransferase-related |
| HORVU3Hr1G072310 | 1,52 | -2,55 | optic atrophy 3 protein (OPA3) |
| HORVU6Hr1G085760 | 1,98 | -2,66 | organic solute transporter-related |
| HORVU7Hr1G050750 | -0,94 | -2,18 | osmotic stress potassium transporter |
| HORVU1Hr1G021750 | 1,71 | -2,74 | peripheral-type benzodiazepine receptor |
| HORVU4Hr1G060510 | 1,79 | -2,15 | peroxisomal membrane protein 2, PXMP2 (MPV17) |
| HORVU6Hr1G086580 | 0,21 | -2,26 | phosphatidylinositol glycan, class T |
| HORVU1Hr1G078350 | -1,34 | -2,24 | phosphoethanolamine N-methyltransferase [EC:2.1.1.103] |
| HORVU2Hr1G023390 | 0,32 | -2,13 | polyketide synthase-related |
| HORVU4Hr1G009550 | 0,67 | -2,37 | probable membrane protein DUF221-related |
| HORVU1Hr1G062630 | 0,89 | -2,57 | probable membrane protein DUF221-related |
| HORVU3Hr1G070490 | 0,87 | -2,84 | profilin |
| HORVU1Hr1G062050 | 0,63 | -2,86 | proprotein convertase subtilisin/kexin |
| HORVU3Hr1G031940 | 1,40 | -2,01 | protease family S26 mitochondrial inner membrane protease-related |
| HORVU1Hr1G020420 | -0,73 | -3,28 | protein glucosyltransferase [EC:2.4.1.-] |
| HORVU5Hr1G068610 | 0,00 | -3,32 | protein phosphatase 2C |
| HORVU3Hr1G064680 | 1,05 | -3,27 | protein transport protein SEC61 subunit alpha |
| HORVU7Hr1G047000 | -0,01 | -2,93 | pyrophosphate--fructose-6-phosphate 1-phosphotransferase [EC:2.7.1.90]; phosphofructokinase |
| HORVU7Hr1G117490 | -0,62 | -3,75 | RAG1-activating protein 1 |
| HORVU2Hr1G083590 | 0,21 | -2,88 | regulator of chromosome condensation |
| HORVU6Hr1G068810 | 1,30 | -2,37 | replication factor C/DNA polymerase III gamma-TAU subunit |
| HORVU4Hr1G062120 | 1,71 | -2,75 | RHO/RAC/CDC GTPase-activating protein |
| HORVU5Hr1G084510 | -0,18 | -2,02 | ribonuclease P 25 kDa subunit-related |
| HORVU2Hr1G081020 | 1,65 | -2,22 | ribosomal protein L7AE family member |
| HORVU4Hr1G025400 | -1,33 | -2,78 | ring finger domain-containing |
| HORVU5Hr1G038840 | 1,45 | -3,65 | RNA binding protein pumilio-related |
| HORVU3Hr1G082880 | -0,81 | -3,43 | RNA slicing protein MRS2, mitochondrial |
| HORVU5Hr1G110840 | -1,79 | -4,22 | S-phase kinase-associated protein 1 |
| HORVU2Hr1G082530 | 0,29 | -2,77 | SEC14 related protein |
| HORVU6Hr1G017580 | 0,29 | -3,68 | SEC14 related protein |
| HORVU4Hr1G014220 | 0,14 | -2,66 | SEC3/Syntaxin-related |
| HORVU1Hr1G034420 | 0,23 | -2,03 | SENTRIN/SUMO-specific protease PROTEASE |
| HORVU3Hr1G028520 | 0,40 | -2,37 | SENTRIN/SUMO-specific protease PROTEASE |
| HORVU5Hr1G069390 | 1,47 | -2,32 | Serine protease family S10 serine carboxypeptidase |
| HORVU7Hr1G116190 | 1,19 | -2,71 | Serine/threonine protein kinase |
| HORVU7Hr1G089510 | -0,62 | -2,15 | Serine/threonine protein kinase |
| HORVU1Hr1G070100 | 0,44 | -2,54 | Serine/threonine protein kinase |
| HORVU6Hr1G058780 | 1,93 | -2,00 | Serine/threonine protein kinase |
| HORVU1Hr1G021880 | -1,96 | -2,05 | seven in absentia homolog |
| HORVU7Hr1G037910 | -0,30 | -2,19 | shikimate kinase [EC:2.7.1.71] |
| HORVU6Hr1G025010 | -0,69 | -2,70 | SHN (SHINE), DNA binding / transcription factor |
| HORVU6Hr1G071960 | -1,23 | -3,29 | solute carrier family 35 |
| HORVU7Hr1G085180 | 0,42 | -2,46 | solute carrier family 35 member B1 (UDP-galactose transporter-related protein 1) (UGTREL1) |
| HORVU3Hr1G076890 | -0,47 | -3,40 | sphingosine kinase [EC:2.7.1.91] |
| HORVU6Hr1G002050 | 1,50 | -4,97 | steroidogenic acute regulatory protein (STAR) |
| HORVU6Hr1G072300 | 1,86 | -2,66 | sterol regulatory element-binding protein |
| HORVU2Hr1G013460 | 0,10 | -3,03 | strictosidine synthase-related |
| HORVU1Hr1G057260 | 0,58 | -2,05 | stromal membrane-associated protein |
| HORVU7Hr1G111530 | 1,56 | -3,28 | sulfate transporter |
| HORVU5Hr1G056780 | -0,50 | -4,23 | syntaxin of plants SYP7 |
| HORVU7Hr1G109940 | 1,08 | -2,29 | TBC1 domain family member GTPase-activating protein |
| HORVU4Hr1G055900 | 1,61 | -2,31 | tetratricopeptide repeat protein, TPR |
| HORVU2Hr1G014360 | 1,39 | -3,10 | thiamine pyrophosphate enzymes |
| HORVU2Hr1G014390 | 1,43 | -3,36 | thiamine pyrophosphate enzymes |
| HORVU4Hr1G007890 | 0,90 | -2,05 | threonine dehydratase [EC:4.3.1.19] |
| HORVU3Hr1G071030 | -0,86 | -2,09 | transcription factor GATA |
| HORVU2Hr1G011690 | 0,72 | -2,02 | transitional endoplasmic reticulum ATPase |
| HORVU2Hr1G097300 | 1,43 | -3,45 | trehalose-6-phosphate synthase |
| HORVU3Hr1G003410 | -0,46 | -4,49 | tryptophan synthase alpha chain [EC:4.2.1.20] |
| HORVU2Hr1G104440 | 0,01 | -2,46 | ubiquitin C |
| HORVU2Hr1G104410 | 1,71 | -3,35 | ubiquitin C |
| HORVU5Hr1G069620 | 0,17 | -2,88 | ubiquitin carboxyl-terminal hydrolase 36/42 [EC:3.1.2.15] |
| HORVU2Hr1G049570 | 1,00 | -5,47 | UDP-glucose pyrophosphorylase |
| HORVU2Hr1G018050 | -1,96 | -2,55 | unknown |
| HORVU6Hr1G034020 | -1,91 | -3,36 | unknown |
| HORVU3Hr1G116010 | -1,74 | -2,66 | unknown |
| HORVU5Hr1G104480 | -1,71 | -2,15 | unknown |
| HORVU5Hr1G078960 | -1,67 | -2,36 | unknown |
| HORVU3Hr1G020280 | -1,59 | -2,75 | unknown |
| HORVU7Hr1G085640 | -1,53 | -2,91 | unknown |
| HORVU4Hr1G082770 | -1,53 | -2,42 | unknown |
| HORVU1Hr1G083310 | -1,50 | -2,40 | unknown |
| HORVU7Hr1G095270 | -1,38 | -2,13 | unknown |
| HORVU5Hr1G103470 | -1,37 | -4,31 | unknown |
| HORVU5Hr1G000380 | -1,35 | -4,41 | unknown |
| HORVU2Hr1G105570 | -1,30 | -2,84 | unknown |
| HORVU2Hr1G118520 | -1,30 | -2,70 | unknown |
| HORVU1Hr1G017190 | -1,14 | -3,56 | unknown |
| HORVU2Hr1G098220 | -1,11 | -2,50 | unknown |
| HORVU6Hr1G073390 | -1,05 | -3,29 | unknown |
| HORVU7Hr1G092980 | -1,03 | -2,55 | unknown |
| HORVU6Hr1G086000 | -1,02 | -3,07 | unknown |
| HORVU0Hr1G000500 | -0,97 | -3,26 | unknown |
| HORVU4Hr1G013630 | -0,95 | -2,10 | unknown |
| HORVU2Hr1G088440 | -0,80 | -3,97 | unknown |
| HORVU2Hr1G114000 | -0,73 | -3,02 | unknown |
| HORVU5Hr1G054870 | -0,70 | -3,26 | unknown |
| HORVU3Hr1G013970 | -0,69 | -2,36 | unknown |
| HORVU4Hr1G010150 | -0,66 | -3,92 | unknown |
| HORVU3Hr1G068400 | -0,61 | -2,51 | unknown |
| HORVU6Hr1G087620 | -0,59 | -3,98 | unknown |
| HORVU2Hr1G109010 | -0,56 | -2,30 | unknown |
| HORVU6Hr1G012030 | -0,53 | -4,08 | unknown |
| HORVU7Hr1G012920 | -0,52 | -2,20 | unknown |
| HORVU7Hr1G008410 | -0,52 | -3,08 | unknown |
| HORVU2Hr1G025570 | -0,52 | -2,25 | unknown |
| HORVU3Hr1G068350 | -0,52 | -2,94 | unknown |
| HORVU6Hr1G087610 | -0,51 | -4,39 | unknown |
| HORVU2Hr1G045590 | -0,51 | -2,44 | unknown |
| HORVU6Hr1G039290 | -0,49 | -2,60 | unknown |
| HORVU2Hr1G019420 | -0,48 | -3,01 | unknown |
| HORVU3Hr1G007150 | -0,45 | -2,01 | unknown |
| HORVU2Hr1G029480 | -0,43 | -2,17 | unknown |
| HORVU7Hr1G105780 | -0,38 | -3,34 | unknown |
| HORVU2Hr1G035200 | -0,38 | -2,55 | unknown |
| HORVU7Hr1G006390 | -0,35 | -2,52 | unknown |
| HORVU0Hr1G004890 | -0,28 | -2,08 | unknown |
| HORVU3Hr1G069860 | -0,25 | -2,78 | unknown |
| HORVU1Hr1G054520 | -0,23 | -3,14 | unknown |
| HORVU4Hr1G070320 | -0,23 | -2,09 | unknown |
| HORVU7Hr1G115880 | -0,22 | -3,38 | unknown |
| HORVU1Hr1G034650 | -0,21 | -3,19 | unknown |
| HORVU1Hr1G075970 | -0,21 | -2,01 | unknown |
| HORVU0Hr1G005690 | -0,20 | -6,99 | unknown |
| HORVU7Hr1G017060 | -0,19 | -2,71 | unknown |
| HORVU2Hr1G027750 | -0,18 | -2,42 | unknown |
| HORVU2Hr1G106720 | -0,18 | -3,55 | unknown |
| HORVU6Hr1G058890 | -0,15 | -2,16 | unknown |
| HORVU7Hr1G115640 | -0,14 | -2,19 | unknown |
| HORVU2Hr1G105800 | -0,11 | -2,70 | unknown |
| HORVU5Hr1G069180 | -0,08 | -2,78 | unknown |
| HORVU7Hr1G097110 | -0,08 | -2,41 | unknown |
| HORVU2Hr1G034570 | -0,05 | -2,13 | unknown |
| HORVU4Hr1G066870 | -0,05 | -3,24 | unknown |
| HORVU2Hr1G073060 | 0,03 | -4,15 | unknown |
| HORVU6Hr1G070690 | 0,05 | -2,88 | unknown |
| HORVU4Hr1G049350 | 0,07 | -2,18 | unknown |
| HORVU2Hr1G009680 | 0,09 | -3,04 | unknown |
| HORVU1Hr1G053300 | 0,09 | -2,77 | unknown |
| HORVU3Hr1G096720 | 0,09 | -2,08 | unknown |
| HORVU1Hr1G063440 | 0,12 | -3,32 | unknown |
| HORVU7Hr1G034430 | 0,15 | -2,74 | unknown |
| HORVU5Hr1G032660 | 0,17 | -2,48 | unknown |
| HORVU5Hr1G119720 | 0,18 | -2,35 | unknown |
| HORVU2Hr1G040000 | 0,19 | -2,10 | unknown |
| HORVU6Hr1G072100 | 0,20 | -3,27 | unknown |
| HORVU2Hr1G005880 | 0,21 | -2,20 | unknown |
| HORVU3Hr1G017080 | 0,23 | -2,20 | unknown |
| HORVU4Hr1G019310 | 0,27 | -3,33 | unknown |
| HORVU6Hr1G090050 | 0,29 | -3,25 | unknown |
| HORVU5Hr1G112030 | 0,32 | -4,43 | unknown |
| HORVU5Hr1G103040 | 0,37 | -2,15 | unknown |
| HORVU3Hr1G017480 | 0,38 | -2,07 | unknown |
| HORVU3Hr1G080310 | 0,41 | -2,00 | unknown |
| HORVU6Hr1G028590 | 0,41 | -2,06 | unknown |
| HORVU0Hr1G004900 | 0,43 | -3,61 | unknown |
| HORVU5Hr1G067680 | 0,44 | -2,35 | unknown |
| HORVU0Hr1G003030 | 0,45 | -3,16 | unknown |
| HORVU4Hr1G037380 | 0,46 | -4,32 | unknown |
| HORVU5Hr1G057690 | 0,48 | -3,98 | unknown |
| HORVU4Hr1G067020 | 0,48 | -2,66 | unknown |
| HORVU4Hr1G087520 | 0,48 | -4,37 | unknown |
| HORVU4Hr1G080030 | 0,50 | -3,36 | unknown |
| HORVU1Hr1G081770 | 0,50 | -2,18 | unknown |
| HORVU3Hr1G030090 | 0,52 | -2,36 | unknown |
| HORVU7Hr1G043880 | 0,53 | -2,46 | unknown |
| HORVU2Hr1G077110 | 0,55 | -2,11 | unknown |
| HORVU6Hr1G025480 | 0,57 | -2,31 | unknown |
| HORVU2Hr1G123730 | 0,58 | -3,18 | unknown |
| HORVU7Hr1G094950 | 0,58 | -2,06 | unknown |
| HORVU3Hr1G115230 | 0,60 | -2,38 | unknown |
| HORVU4Hr1G057790 | 0,66 | -2,43 | unknown |
| HORVU7Hr1G026860 | 0,67 | -2,12 | unknown |
| HORVU3Hr1G112250 | 0,67 | -4,18 | unknown |
| HORVU3Hr1G039300 | 0,83 | -2,27 | unknown |
| HORVU3Hr1G058150 | 0,84 | -2,81 | unknown |
| HORVU6Hr1G020790 | 0,85 | -2,70 | unknown |
| HORVU4Hr1G008810 | 0,86 | -3,17 | unknown |
| HORVU3Hr1G029370 | 0,90 | -2,89 | unknown |
| HORVU2Hr1G107060 | 0,91 | -2,07 | unknown |
| HORVU0Hr1G029310 | 0,92 | -2,12 | unknown |
| HORVU5Hr1G105780 | 0,93 | -2,20 | unknown |
| HORVU7Hr1G002050 | 0,93 | -2,83 | unknown |
| HORVU3Hr1G037040 | 0,95 | -2,02 | unknown |
| HORVU3Hr1G052010 | 0,97 | -2,38 | unknown |
| HORVU2Hr1G039200 | 0,97 | -2,52 | unknown |
| HORVU2Hr1G112440 | 1,01 | -2,49 | unknown |
| HORVU4Hr1G026810 | 1,01 | -2,12 | unknown |
| HORVU4Hr1G085130 | 1,02 | -3,16 | unknown |
| HORVU1Hr1G094250 | 1,05 | -2,10 | unknown |
| HORVU4Hr1G083850 | 1,07 | -2,01 | unknown |
| HORVU3Hr1G036600 | 1,07 | -2,26 | unknown |
| HORVU0Hr1G016830 | 1,09 | -2,32 | unknown |
| HORVU7Hr1G043230 | 1,10 | -2,52 | unknown |
| HORVU5Hr1G086480 | 1,11 | -2,95 | unknown |
| HORVU4Hr1G039870 | 1,11 | -2,11 | unknown |
| HORVU1Hr1G045510 | 1,12 | -4,15 | unknown |
| HORVU4Hr1G074110 | 1,14 | -2,19 | unknown |
| HORVU2Hr1G100250 | 1,16 | -2,28 | unknown |
| HORVU5Hr1G015430 | 1,17 | -2,21 | unknown |
| HORVU0Hr1G024130 | 1,18 | -2,53 | unknown |
| HORVU1Hr1G094240 | 1,21 | -4,02 | unknown |
| HORVU6Hr1G010990 | 1,25 | -2,61 | unknown |
| HORVU6Hr1G070740 | 1,25 | -2,49 | unknown |
| HORVU4Hr1G087350 | 1,26 | -2,42 | unknown |
| HORVU2Hr1G015710 | 1,26 | -2,91 | unknown |
| HORVU5Hr1G046020 | 1,28 | -2,30 | unknown |
| HORVU1Hr1G051700 | 1,28 | -2,55 | unknown |
| HORVU2Hr1G072060 | 1,31 | -2,94 | unknown |
| HORVU6Hr1G023790 | 1,32 | -3,11 | unknown |
| HORVU0Hr1G023890 | 1,33 | -2,04 | unknown |
| HORVU3Hr1G113930 | 1,34 | -4,31 | unknown |
| HORVU4Hr1G084020 | 1,35 | -2,20 | unknown |
| HORVU6Hr1G082220 | 1,39 | -3,53 | unknown |
| HORVU5Hr1G039660 | 1,39 | -2,02 | unknown |
| HORVU1Hr1G093500 | 1,44 | -2,60 | unknown |
| HORVU1Hr1G040130 | 1,49 | -2,08 | unknown |
| HORVU3Hr1G115940 | 1,51 | -2,09 | unknown |
| HORVU3Hr1G109250 | 1,58 | -3,77 | unknown |
| HORVU5Hr1G092750 | 1,58 | -2,55 | unknown |
| HORVU6Hr1G072260 | 1,59 | -3,12 | unknown |
| HORVU2Hr1G114280 | 1,60 | -2,00 | unknown |
| HORVU2Hr1G072890 | 1,62 | -2,28 | unknown |
| HORVU1Hr1G056470 | 1,65 | -2,11 | unknown |
| HORVU4Hr1G058410 | 1,72 | -3,38 | unknown |
| HORVU5Hr1G067230 | 1,75 | -2,07 | unknown |
| HORVU3Hr1G000110 | 1,80 | -2,23 | unknown |
| HORVU3Hr1G030840 | 1,87 | -3,31 | unknown |
| HORVU4Hr1G008680 | 1,89 | -2,12 | unknown |
| HORVU7Hr1G027380 | 1,92 | -3,38 | unknown |
| HORVU7Hr1G025110 | 1,93 | -2,04 | unknown |
| HORVU7Hr1G075130 | 1,94 | -2,24 | unknown |
| HORVU5Hr1G103060 | 1,98 | -2,41 | unknown |
| HORVU1Hr1G072750 | 1,96 | -2,09 | uracil phosphoribosyltransferase [EC:2.4.2.9] |
| HORVU7Hr1G110830 | 0,05 | -2,73 | villin |
| HORVU3Hr1G067610 | 0,71 | -3,22 | voltage-dependent anion-selective channel |
| HORVU2Hr1G119220 | -0,60 | -3,81 | WD repeat domain 44 |
| HORVU6Hr1G065020 | -0,11 | -2,28 | WNK lysine deficient protein kinase [EC:2.7.11.1] |
| HORVU7Hr1G035590 | 0,67 | -2,42 | xyloglucan fucosyltransferase [EC:2.4.1.-] |
