# Supplementary material for: Differential Expression Profiling of Microspores During the Early Stages of Isolated Microspore Culture Using the Responsive Barley Cultivar Gobernadora
Source: G3 (Bethesda). 2018 Mar 12;8(5):1603–14. doi: 10.1534/g3.118.200208 (PMC5940152; doi:10.1534/g3.118.200208)
Supplement: Supplementary file 4 [file 1603TableS4.docx]

Supplementary Table 4: Gene functional annotation for genes in cluster 4

| Gene stable ID (cluster 4) | logFC D2-D0 | logFC D5-D2 | Gene function annotation |
| --- | --- | --- | --- |
| HORVU2Hr1G094230 | 3,20 | 3,94 | 1-aminocyclopropane-1-carboxylate synthase [EC:4.4.1.14] |
| HORVU5Hr1G023640 | 2,89 | 2,65 | 1,4-beta-D-xylan synthase [EC:2.4.2.24] |
| HORVU7Hr1G036070 | 3,58 | 3,48 | 12-oxophytodienoic acid reductase [EC:1.3.1.42] |
| HORVU5Hr1G034820 | 2,90 | 5,97 | 2-oxoisovalerate dehydrogenase E1 component, alpha subunit [EC:1.2.4.4] |
| HORVU6Hr1G027650 | 2,02 | 2,62 | 4-hydroxyphenylpyruvate dioxygenase [EC:1.13.11.27] |
| HORVU1Hr1G017240 | 2,14 | 3,54 | 5'-AMP-activated protein kinase, catalytic alpha subunit [EC:2.7.11.11] |
| HORVU1Hr1G056510 | 2,14 | 2,02 | 50S ribosomal protein L1 |
| HORVU3Hr1G108680 | 2,67 | 6,16 | achaete-scute transcription factor |
| HORVU5Hr1G076250 | 3,59 | 3,59 | acid phosphatase |
| HORVU3Hr1G097860 | 5,11 | 4,60 | adenylyl-sulfate reductase (glutathione) [EC:1.8.4.9] |
| HORVU0Hr1G012940 | 5,36 | 3,07 | alcohol dehydrogenase; cinnamyl-alcohol dehydrogenase [EC:1.1.1.195] |
| HORVU6Hr1G087220 | 2,47 | 2,56 | alcohol dehydrogenase; cinnamyl-alcohol dehydrogenase [EC:1.1.1.195] |
| HORVU2Hr1G092530 | 2,37 | 5,88 | aldehyde dehydrogenase |
| HORVU2Hr1G082700 | 2,53 | 5,33 | aldo/keto reductase |
| HORVU2Hr1G082740 | 3,55 | 5,25 | aldo/keto reductase |
| HORVU1Hr1G048530 | 3,37 | 2,70 | alpha/beta hydrolase fold-containing protein |
| HORVU7Hr1G076910 | 3,77 | 2,70 | alpha/beta hydrolase fold-containing protein |
| HORVU1Hr1G094180 | 4,03 | 6,92 | alpha/beta hydrolase fold-containing protein |
| HORVU6Hr1G005400 | 4,61 | 2,15 | alpha/beta hydrolase fold-containing protein |
| HORVU4Hr1G019600 | 2,97 | 6,47 | amidase |
| HORVU7Hr1G074660 | 2,93 | 4,74 | amino acid transporter |
| HORVU2Hr1G069060 | 3,00 | 4,99 | AMP-activated protein, gamma regulatory subunit |
| HORVU5Hr1G122820 | 4,41 | 2,74 | ankyrin repeat protein |
| HORVU4Hr1G079230 | 3,07 | 3,66 | aquaporin TIP |
| HORVU5Hr1G105840 | 6,20 | 2,78 | asparagine synthase |
| HORVU5Hr1G040970 | 4,02 | 4,95 | asparagine synthase; glutamine amidotransferase |
| HORVU7Hr1G089290 | 4,71 | 4,77 | aspartate aminotransferase, mitchondrial [EC:2.6.1.1] |
| HORVU4Hr1G088530 | 2,22 | 6,35 | aspartyl proteases |
| HORVU7Hr1G006700 | 2,58 | 5,32 | aspartyl proteases |
| HORVU3Hr1G009980 | 3,47 | 3,97 | aspartyl proteases |
| HORVU7Hr1G116810 | 3,90 | 4,66 | aspartyl proteases |
| HORVU3Hr1G091680 | 4,30 | 5,04 | aspartyl proteases |
| HORVU0Hr1G016890 | 4,47 | 6,23 | aspartyl proteases |
| HORVU7Hr1G118020 | 4,61 | 6,27 | aspartyl proteases |
| HORVU5Hr1G070400 | 3,06 | 3,26 | ATP-binding cassette transporter |
| HORVU2Hr1G090960 | 3,64 | 7,88 | ATP-binding cassette transporter |
| HORVU7Hr1G063020 | 2,03 | 7,26 | ATP-binding cassette transporter |
| HORVU1Hr1G087460 | 2,45 | 3,60 | auxin response factor |
| HORVU1Hr1G066340 | 3,60 | 8,40 | auxin responsive GH3 gene family |
| HORVU5Hr1G081180 | 2,07 | 6,80 | auxin-responsive protein IAA |
| HORVU3Hr1G022540 | 2,79 | 3,88 | auxin-responsive protein IAA |
| HORVU5Hr1G106350 | 3,84 | 3,65 | auxin-responsive protein IAA |
| HORVU5Hr1G107410 | 2,59 | 3,85 | BCS1 AAA-type ATPase |
| HORVU7Hr1G098220 | 2,95 | 3,97 | BCS1 AAA-type ATPase |
| HORVU1Hr1G063390 | 5,56 | 2,61 | BCS1 AAA-type ATPase |
| HORVU1Hr1G048760 | 3,02 | 2,55 | beta catenin-related armadillo repeat-containing |
| HORVU3Hr1G081300 | 3,81 | 6,31 | beta catenin-related armadillo repeat-containing |
| HORVU4Hr1G063640 | 4,13 | 2,76 | beta catenin-related armadillo repeat-containing |
| HORVU7Hr1G038370 | 2,39 | 2,45 | beta-1,3-galactosyltransferase [EC:2.4.1.-] |
| HORVU4Hr1G005300 | 2,03 | 4,39 | beta-1,3-N-acetylglucosaminyltransferase |
| HORVU4Hr1G005270 | 2,51 | 4,55 | beta-1,3-N-acetylglucosaminyltransferase |
| HORVU5Hr1G108670 | 2,62 | 2,40 | beta-1,3-N-acetylglucosaminyltransferase |
| HORVU4Hr1G086300 | 6,31 | 2,41 | beta-fructofuranosidase [EC:3.2.1.26] |
| HORVU7Hr1G086690 | 2,67 | 5,63 | beta-galactosidase |
| HORVU5Hr1G077920 | 4,89 | 3,58 | beta-glucosidase [EC:3.2.1.21] |
| HORVU7Hr1G082280 | 4,02 | 4,36 | caffeic acid 3-O-methyltransferase [EC:2.1.1.68]; flavonol 3-O-methyltransferase [EC:2.1.1.76] |
| HORVU2Hr1G088110 | 4,09 | 3,13 | calcium-activated chloride channel regulator |
| HORVU7Hr1G115410 | 2,59 | 2,36 | calcium-binding protein CML |
| HORVU3Hr1G109230 | 3,91 | 2,97 | calcium-binding protein CML |
| HORVU5Hr1G085800 | 3,96 | 3,82 | calcium-binding protein CML |
| HORVU5Hr1G014060 | 4,18 | 4,45 | calcium-binding protein CML |
| HORVU2Hr1G046660 | 2,44 | 2,38 | calcium-dependent protein kinase [EC:2.7.11.1]; serine/threonine-proteine kinase |
| HORVU1Hr1G053080 | 3,07 | 3,05 | carotenoid epsilon-ring hydroxylase |
| HORVU0Hr1G039050 | 5,28 | 2,39 | centaurin/ARF |
| HORVU2Hr1G023940 | 2,08 | 2,31 | CGI-141-related/lipase containing protein |
| HORVU1Hr1G072250 | 2,54 | 7,54 | chitinase |
| HORVU2Hr1G043890 | 2,09 | 7,31 | chitinase [EC:3.2.1.14] |
| HORVU7Hr1G113270 | 5,10 | 3,40 | chitinase [EC:3.2.1.14] |
| HORVU3Hr1G064470 | 5,21 | 4,05 | chitinase [EC:3.2.1.14] |
| HORVU3Hr1G113120 | 3,58 | 4,49 | chitinase-related |
| HORVU2Hr1G085280 | 5,68 | 2,78 | chitinase-related |
| HORVU3Hr1G018530 | 5,43 | 2,15 | chromate reductase |
| HORVU4Hr1G063830 | 2,47 | 2,57 | cofilin |
| HORVU5Hr1G111870 | 2,62 | 2,30 | copine |
| HORVU2Hr1G032280 | 3,14 | 3,23 | copper transport protein ATOX1-related |
| HORVU2Hr1G118230 | 3,54 | 3,80 | copper transport protein ATOX1-related |
| HORVU2Hr1G118060 | 3,61 | 2,62 | copper transport protein ATOX1-related |
| HORVU3Hr1G034110 | 3,78 | 4,98 | copper transport protein ATOX1-related |
| HORVU2Hr1G011060 | 3,84 | 4,00 | copper transport protein ATOX1-related |
| HORVU7Hr1G121820 | 3,17 | 3,77 | cyclin B |
| HORVU4Hr1G016990 | 2,87 | 3,92 | cysteine desulfurylase |
| HORVU5Hr1G089970 | 3,77 | 4,70 | cysteine protease family C1-related |
| HORVU2Hr1G044680 | 2,22 | 4,62 | cysteine-rich repeat secretory protein |
| HORVU2Hr1G044590 | 2,54 | 4,23 | cysteine-rich repeat secretory protein |
| HORVU2Hr1G044520 | 3,79 | 4,29 | cysteine-rich repeat secretory protein |
| HORVU2Hr1G044510 | 4,20 | 3,22 | cysteine-rich repeat secretory protein |
| HORVU3Hr1G028620 | 6,04 | 2,37 | cytochrome P450, family 710, subfamily A |
| HORVU3Hr1G018860 | 3,15 | 4,01 | cytochrome P450, family 90, subfamily D, polypeptide 2 (steroid 3-oxidase) [EC:1.14.-.-] |
| HORVU4Hr1G006930 | 3,02 | 4,24 | DELLA protein |
| HORVU7Hr1G087900 | 2,45 | 2,74 | diacylglycerol O-acyltransferase 1 [EC:2.3.1.20 2.3.1.75 2.3.1.76] |
| HORVU1Hr1G062080 | 2,61 | 3,46 | dienelactone hydrolase |
| HORVU4Hr1G072260 | 7,63 | 3,17 | dimethylaniline monooxygenase |
| HORVU3Hr1G071800 | 3,16 | 3,64 | DUF814 |
| HORVU3Hr1G025090 | 4,66 | 2,72 | dynamin |
| HORVU3Hr1G073190 | 4,65 | 2,25 | dynein light chain |
| HORVU4Hr1G056260 | 3,50 | 3,75 | E3 ubiquitin-protein ligase SIAH1 [EC:6.3.2.19] |
| HORVU5Hr1G072700 | 5,89 | 2,21 | EF-hand calcium-binding domain containing protein |
| HORVU6Hr1G022270 | 2,92 | 2,83 | endo-1,4-beta-glucanase |
| HORVU2Hr1G085800 | 3,51 | 4,17 | endo-1,4-beta-glucanase |
| HORVU6Hr1G077010 | 3,78 | 3,74 | endo-1,4-beta-glucanase |
| HORVU5Hr1G066720 | 5,22 | 3,12 | epididymal membrane protein E9-related |
| HORVU4Hr1G077360 | 2,05 | 5,92 | EREBP-like factor |
| HORVU1Hr1G060060 | 3,11 | 7,20 | EREBP-like factor |
| HORVU2Hr1G018830 | 5,84 | 2,50 | EREBP-like factor |
| HORVU4Hr1G006260 | 2,58 | 4,19 | ethylene receptor [EC:2.7.13.-]; two-component sensor histidine kinase |
| HORVU6Hr1G071860 | 3,92 | 7,14 | ethylene receptor [EC:2.7.13.-]; two-component sensor histidine kinase |
| HORVU4Hr1G051680 | 4,11 | 3,79 | ethylene-insensitive protein 3 |
| HORVU2Hr1G045050 | 4,44 | 2,20 | eukaryotic translation initiation factor 2C |
| HORVU7Hr1G054020 | 2,67 | 4,77 | exocyst complex protein EXO70 |
| HORVU4Hr1G079010 | 2,55 | 5,32 | exostosin(heparan sulfate glycosyltransferase)-related |
| HORVU7Hr1G095810 | 2,11 | 2,58 | extracellular signal-regulated kinase 1/2 [EC:2.7.11.24]; mitogen-activated protein kinase |
| HORVU5Hr1G017260 | 2,77 | 3,37 | F-BOX and WD40 domain protein |
| HORVU6Hr1G027930 | 4,68 | 4,85 | flotillin |
| HORVU2Hr1G079960 | 2,91 | 5,24 | gamma glutamyl transpeptidases |
| HORVU1Hr1G090020 | 3,61 | 2,92 | glucose-6-phosphate 1-epimerase [EC:5.1.3.15] |
| HORVU3Hr1G035730 | 4,18 | 4,09 | glucose-methanol-choline (GMC) oxidoreductase |
| HORVU1Hr1G081890 | 2,06 | 6,39 | glucosyl/glucuronosyl transferases |
| HORVU5Hr1G111040 | 2,57 | 5,03 | glucosyl/glucuronosyl transferases |
| HORVU2Hr1G090200 | 2,97 | 4,67 | glucosyl/glucuronosyl transferases |
| HORVU3Hr1G021810 | 3,06 | 4,88 | glucosyl/glucuronosyl transferases |
| HORVU3Hr1G023400 | 3,30 | 3,91 | glucosyl/glucuronosyl transferases |
| HORVU7Hr1G101710 | 4,09 | 5,67 | glucosyl/glucuronosyl transferases |
| HORVU2Hr1G090170 | 4,58 | 2,61 | glucosyl/glucuronosyl transferases |
| HORVU2Hr1G028580 | 4,77 | 2,39 | glucosyl/glucuronosyl transferases |
| HORVU6Hr1G077860 | 4,89 | 2,99 | glucosyl/glucuronosyl transferases |
| HORVU4Hr1G063660 | 5,22 | 2,27 | glucosyl/glucuronosyl transferases |
| HORVU6Hr1G035370 | 5,22 | 2,28 | glucosyl/glucuronosyl transferases |
| HORVU4Hr1G007610 | 2,69 | 3,18 | glutamine synthetase [EC:6.3.1.2] |
| HORVU3Hr1G061080 | 3,21 | 2,00 | glutaredoxin |
| HORVU0Hr1G017160 | 3,73 | 3,92 | glutaredoxin |
| HORVU1Hr1G021140 | 2,51 | 4,09 | glutathione S-transferase [EC:2.5.1.18] |
| HORVU5Hr1G045850 | 2,54 | 2,70 | glutathione S-transferase [EC:2.5.1.18] |
| HORVU1Hr1G017500 | 2,86 | 2,44 | glutathione S-transferase [EC:2.5.1.18] |
| HORVU3Hr1G024500 | 2,97 | 2,10 | glutathione S-transferase [EC:2.5.1.18] |
| HORVU1Hr1G049270 | 3,66 | 4,87 | glutathione S-transferase [EC:2.5.1.18] |
| HORVU5Hr1G103430 | 4,58 | 2,65 | glutathione S-transferase [EC:2.5.1.18] |
| HORVU1Hr1G049280 | 4,81 | 3,27 | glutathione S-transferase [EC:2.5.1.18] |
| HORVU1Hr1G049210 | 4,82 | 4,10 | glutathione S-transferase [EC:2.5.1.18] |
| HORVU1Hr1G049070 | 5,31 | 3,07 | glutathione S-transferase [EC:2.5.1.18] |
| HORVU0Hr1G019300 | 5,33 | 2,04 | glutathione S-transferase [EC:2.5.1.18] |
| HORVU1Hr1G021180 | 6,17 | 3,44 | glutathione S-transferase [EC:2.5.1.18] |
| HORVU4Hr1G057740 | 6,37 | 2,80 | glutathione S-transferase [EC:2.5.1.18] |
| HORVU6Hr1G026810 | 5,09 | 2,94 | glutathione S-transferase [EC:2.5.1.18] |
| HORVU3Hr1G002920 | 2,30 | 5,46 | glycosyltransferase |
| HORVU7Hr1G048570 | 2,59 | 3,74 | glycosyltransferase |
| HORVU7Hr1G048430 | 2,85 | 3,65 | glycosyltransferase |
| HORVU1Hr1G004980 | 5,76 | 2,32 | glycosyltransferase |
| HORVU3Hr1G058900 | 7,01 | 2,23 | glycosyltransferase |
| HORVU1Hr1G036120 | 2,97 | 3,87 | glycosyltransferase 14 |
| HORVU4Hr1G004260 | 4,78 | 3,54 | GMP synthase |
| HORVU4Hr1G029760 | 2,27 | 2,02 | GTP-binding protein HflX |
| HORVU3Hr1G027430 | 2,35 | 4,15 | glulonolactone oxidase |
| HORVU7Hr1G075760 | 4,86 | 3,28 | glulonolactone oxidase |
| HORVU4Hr1G059860 | 2,39 | 4,83 | haloacid dehalogenase-like hydrolase |
| HORVU4Hr1G006100 | 2,71 | 3,72 | haloacid dehalogenase-like hydrolase |
| HORVU5Hr1G068320 | 4,72 | 3,73 | heat shock transcription factor, other eukaryote |
| HORVU4Hr1G090090 | 7,81 | 2,76 | heat shock transcription factor, other eukaryote |
| HORVU4Hr1G068830 | 2,21 | 3,28 | heparanase 1 [EC:3.2.1.-] |
| HORVU7Hr1G012850 | 2,92 | 2,79 | histone H1/5 |
| HORVU2Hr1G106970 | 2,04 | 6,64 | homeobox-leucine zipper protein |
| HORVU4Hr1G065900 | 2,83 | 5,56 | homeobox-leucine zipper protein |
| HORVU5Hr1G103890 | 2,93 | 4,54 | indole-3-acetaldehyde oxidase [EC:1.2.3.7]; xanthine dehydrogenase |
| HORVU5Hr1G106190 | 4,32 | 3,14 | indole-3-glycerol-phosphate lyase [EC:4.1.2.8]; ser/thr dehydratase, trp synthase |
| HORVU6Hr1G066640 | 2,22 | 2,95 | inhibitor of apoptosis |
| HORVU6Hr1G020210 | 2,94 | 3,56 | interleukin-1 receptor-associated kinase 1 [EC:2.7.11.1] |
| HORVU4Hr1G081590 | 4,02 | 3,42 | interleukin-1 receptor-associated kinase 1 [EC:2.7.11.1]; chitinase-related |
| HORVU1Hr1G025530 | 5,05 | 2,30 | lactoylglutathione lyase (glyoxalase I) |
| HORVU4Hr1G051780 | 4,58 | 4,88 | late embryogenesis abundant (LEA) |
| HORVU1Hr1G082820 | 2,04 | 6,72 | late embryogenesis abundant (LEA) |
| HORVU2Hr1G075930 | 2,50 | 6,47 | late embryogenesis abundant (LEA) |
| HORVU3Hr1G113640 | 3,21 | 3,15 | leucine-rich repeat-containing protein |
| HORVU0Hr1G008580 | 3,45 | 5,19 | leucine-rich repeat-containing protein; disease resistance protein RPS2 |
| HORVU5Hr1G099350 | 2,46 | 4,28 | long-chain acyl-CoA synthetase [EC:6.2.1.3] |
| HORVU2Hr1G031410 | 2,99 | 3,22 | lung seven transmembrane receptor |
| HORVU2Hr1G120980 | 2,86 | 2,45 | lysophospholipase III [EC:3.1.1.5]; lecithin-cholesterol acyltransferase-related |
| HORVU3Hr1G006600 | 2,54 | 2,37 | lyst-interacting protein LIP5 (dopamine responsive protein DRG-1) |
| HORVU2Hr1G101690 | 3,06 | 3,77 | mamary turmor virus receptor homolog 1,2 (MTVR1, 2) |
| HORVU3Hr1G101210 | 2,09 | 2,57 | member of 'GDXG' family of lipolytic enzymes |
| HORVU5Hr1G098770 | 2,41 | 5,99 | member of 'GDXG' family of lipolytic enzymes |
| HORVU4Hr1G062060 | 4,17 | 5,74 | member of 'GDXG' family of lipolytic enzymes |
| HORVU2Hr1G095070 | 2,64 | 4,13 | methyltransferase |
| HORVU1Hr1G070690 | 2,12 | 3,50 | MFS transporter, UMF1 family |
| HORVU1Hr1G086450 | 5,73 | 3,33 | MFS transporter, UMF1 family; pirin-related |
| HORVU1Hr1G078710 | 2,33 | 3,69 | mitogen-activated kinase |
| HORVU3Hr1G065610 | 3,60 | 4,18 | mitogen-activated kinase |
| HORVU1Hr1G064440 | 5,49 | 3,40 | mlo protein |
| HORVU4Hr1G072300 | 4,91 | 3,99 | monooxygenase |
| HORVU4Hr1G072310 | 4,93 | 2,38 | monooxygenase |
| HORVU7Hr1G000150 | 2,61 | 4,47 | multi-copper oxidase |
| HORVU3Hr1G004360 | 4,14 | 5,39 | multi-copper oxidase |
| HORVU4Hr1G078230 | 3,69 | 2,45 | multidrug resistance protein, MATE family |
| HORVU1Hr1G074260 | 3,52 | 3,99 | myb proto-oncogene protein, plant |
| HORVU3Hr1G082210 | 5,77 | 5,72 | myb proto-oncogene protein, plant |
| HORVU3Hr1G066390 | 7,64 | 2,82 | MYC |
| HORVU3Hr1G050290 | 2,66 | 2,42 | myosin V |
| HORVU7Hr1G056490 | 4,33 | 2,48 | myrosinase binding protein-related |
| HORVU5Hr1G097440 | 2,26 | 8,39 | N-terminal acetyltransferase |
| HORVU5Hr1G078630 | 3,23 | 5,56 | NADPH oxidase |
| HORVU6Hr1G052500 | 3,29 | 2,69 | NIMA (never in mitosis gene a)-related kinase [EC:2.7.11.1] |
| HORVU6Hr1G003300 | 4,72 | 5,88 | nitrate reductase (NADPH) [EC:1.7.1.3] |
| HORVU1Hr1G062380 | 5,37 | 2,79 | nitrate, fromate, iron dehydrogenase |
| HORVU4Hr1G075830 | 2,17 | 2,38 | nuclear transcription factor Y, alpha |
| HORVU5Hr1G094700 | 4,00 | 2,96 | nucleoporin-related |
| HORVU7Hr1G086170 | 5,19 | 2,53 | oligopeptide transporter-related |
| HORVU5Hr1G067380 | 3,19 | 2,44 | osmotic stress potassium transporter |
| HORVU2Hr1G071570 | 2,56 | 6,91 | osmotic stress potassium transporter |
| HORVU2Hr1G082370 | 2,22 | 5,83 | oxidoreductase, 20G-FE(II) oxygenase family protein |
| HORVU1Hr1G090870 | 2,58 | 3,92 | oxidoreductase, 20G-FE(II) oxygenase family protein |
| HORVU5Hr1G065620 | 3,28 | 6,21 | oxidoreductase, 20G-FE(II) oxygenase family protein |
| HORVU1Hr1G056210 | 4,32 | 2,44 | oxidoreductase, 20G-FE(II) oxygenase family protein |
| HORVU6Hr1G084030 | 2,15 | 5,06 | pectinesterase [EC:3.1.1.11] |
| HORVU2Hr1G032220 | 4,34 | 4,01 | pectinesterase [EC:3.1.1.11] |
| HORVU1Hr1G057880 | 3,10 | 3,19 | peptide chain release factor eRF subunit 1 |
| HORVU4Hr1G084920 | 2,09 | 3,17 | peroxidase [EC:1.11.1.7] |
| HORVU0Hr1G002840 | 2,26 | 8,31 | peroxidase [EC:1.11.1.7] |
| HORVU1Hr1G016660 | 2,64 | 4,86 | peroxidase [EC:1.11.1.7] |
| HORVU4Hr1G022270 | 2,69 | 5,32 | peroxidase [EC:1.11.1.7] |
| HORVU3Hr1G074920 | 2,96 | 5,08 | peroxidase [EC:1.11.1.7] |
| HORVU6Hr1G021520 | 3,64 | 2,04 | peroxidase [EC:1.11.1.7] |
| HORVU6Hr1G014050 | 4,58 | 6,39 | peroxidase [EC:1.11.1.7] |
| HORVU3Hr1G074960 | 5,28 | 4,77 | peroxidase [EC:1.11.1.7] |
| HORVU2Hr1G064460 | 5,83 | 3,64 | peroxidase [EC:1.11.1.7] |
| HORVU3Hr1G077580 | 6,48 | 2,48 | peroxidase [EC:1.11.1.7] |
| HORVU3Hr1G037630 | 2,10 | 7,41 | phenazine biosynthesis protein |
| HORVU2Hr1G089440 | 2,80 | 5,23 | phenylalanine ammonia-lyase [EC:4.3.1.24]; histidine ammonia-lyase |
| HORVU4Hr1G062440 | 2,12 | 5,31 | phosphoenolpyruvate carboxykinase (ATP) [EC:4.1.1.49] |
| HORVU7Hr1G089960 | 2,50 | 2,38 | phospholipase D [EC:3.1.4.4] |
| HORVU0Hr1G015950 | 3,14 | 4,12 | phytoene dehydrogenase |
| HORVU1Hr1G077820 | 2,94 | 4,31 | poly [ADP-ribose] polymerase [EC:2.4.2.30]; DNA ligase |
| HORVU7Hr1G042240 | 2,64 | 4,70 | PQQ oxidoreductase-related |
| HORVU1Hr1G048860 | 5,94 | 4,83 | prephenate dehydratase (P protein) |
| HORVU1Hr1G051200 | 2,38 | 2,17 | probable membrane protein DUF221-related |
| HORVU2Hr1G060390 | 2,79 | 3,52 | probable membrane protein DUF221-related |
| HORVU3Hr1G045270 | 4,87 | 2,08 | probable membrane protein DUF221-related |
| HORVU3Hr1G074820 | 2,05 | 2,64 | proprotein convertase subtilisin/kexin |
| HORVU2Hr1G094660 | 2,63 | 4,55 | proprotein convertase subtilisin/kexin |
| HORVU4Hr1G013170 | 3,89 | 5,92 | proprotein convertase subtilisin/kexin |
| HORVU1Hr1G074820 | 4,48 | 4,04 | proprotein convertase subtilisin/kexin |
| HORVU4Hr1G085590 | 5,02 | 3,85 | proprotein convertase subtilisin/kexin |
| HORVU7Hr1G036000 | 3,93 | 2,06 | protease family S9B,C dipeptidyl-peptidase IV-related |
| HORVU5Hr1G113240 | 2,36 | 4,14 | protein phosphatase 2C |
| HORVU4Hr1G060370 | 3,26 | 3,15 | protein phosphatase 2C [EC:3.1.3.16] |
| HORVU0Hr1G016180 | 2,92 | 4,58 | putative drug exporter of the RND superfamily |
| HORVU1Hr1G061160 | 7,06 | 2,13 | pyruvate,orthophosphate dikinase [EC:2.7.9.1] |
| HORVU6Hr1G055960 | 2,86 | 2,93 | RAG1-activating protein 1 |
| HORVU7Hr1G030160 | 4,60 | 4,85 | RAG1-activating protein 1 |
| HORVU6Hr1G076210 | 2,29 | 4,17 | Ras-related C3 botulinum toxin substrate 1 |
| HORVU1Hr1G076880 | 2,83 | 5,78 | Ras-related C3 botulinum toxin substrate 1 |
| HORVU4Hr1G081670 | 4,11 | 3,34 | respiratory burst oxidase [EC:1.6.3.- 1.11.1.-] |
| HORVU3Hr1G069780 | 3,30 | 3,04 | respiratory burst oxidase [EC:1.6.3.- 1.11.1.-]; NADPH OXIDASE |
| HORVU1Hr1G095170 | 3,01 | 3,04 | Rho GDP-dissociation inhibitor |
| HORVU4Hr1G027850 | 2,10 | 3,16 | rhomboid-related |
| HORVU7Hr1G056630 | 3,32 | 3,95 | rhomboid-related |
| HORVU1Hr1G038130 | 3,90 | 5,32 | RING FINGER and CHY ZINC FINGER domain-containing protein 1 |
| HORVU6Hr1G091800 | 2,61 | 3,26 | RING FINGER containing protein |
| HORVU5Hr1G016710 | 2,17 | 3,91 | RING FINGER containing protein |
| HORVU6Hr1G066050 | 2,70 | 3,56 | RING FINGER containing protein |
| HORVU5Hr1G082830 | 3,43 | 2,26 | RING FINGER containing protein |
| HORVU3Hr1G028440 | 4,02 | 4,07 | RING FINGER containing protein |
| HORVU2Hr1G113930 | 4,10 | 2,61 | RING FINGER containing protein |
| HORVU7Hr1G057650 | 4,57 | 2,47 | RING FINGER containing protein |
| HORVU5Hr1G069980 | 4,78 | 4,16 | RING FINGER containing protein |
| HORVU6Hr1G009820 | 5,61 | 4,41 | RING FINGER containing protein |
| HORVU6Hr1G077610 | 5,91 | 4,92 | RING FINGER containing protein |
| HORVU6Hr1G074220 | 2,13 | 2,18 | RNA-dependent RNA polymerase 1 [EC:2.7.7.48] |
| HORVU1Hr1G075150 | 2,80 | 3,32 | SEC14 related protein |
| HORVU7Hr1G107070 | 3,45 | 3,32 | SEC14 related protein |
| HORVU4Hr1G073120 | 5,04 | 5,59 | seed maturation family protein |
| HORVU2Hr1G093800 | 2,60 | 2,13 | senataxin [EC:3.6.4.-]; DNA2/NAM7 helicase family |
| HORVU6Hr1G066280 | 2,75 | 6,87 | senataxin [EC:3.6.4.-]; DNA2/NAM7 helicase family |
| HORVU4Hr1G071040 | 5,80 | 2,71 | serine O-acetyltransferase [EC:2.3.1.30] |
| HORVU5Hr1G080090 | 3,06 | 4,31 | serine protease family S10 serine carboxypeptidase |
| HORVU6Hr1G059550 | 3,81 | 2,66 | serine protease family S10 serine carboxypeptidase |
| HORVU4Hr1G013540 | 2,14 | 3,42 | serine/threonine-protein kinase SRK2 [EC:2.7.11.1] |
| HORVU5Hr1G065370 | 4,85 | 4,00 | serine/threonine-protein kinase; 5'-AMP-activated protein kinase, catalytic alpha subunit [EC:2.7.11.11] |
| HORVU7Hr1G118090 | 3,67 | 4,19 | shikimate O-hydroxycinnamoyltransferase [EC:2.3.1.133] |
| HORVU5Hr1G067800 | 2,42 | 5,46 | signal peptidase I [EC:3.4.21.89]; protease family S26 mitochondrial inner membrane protease-related |
| HORVU4Hr1G079710 | 3,84 | 2,46 | solute carrier family 13 member |
| HORVU7Hr1G007220 | 2,56 | 2,56 | sucrose synthase [EC:2.4.1.13] |
| HORVU6Hr1G094880 | 2,72 | 2,14 | sucrose synthase [EC:2.4.1.13] |
| HORVU4Hr1G075200 | 5,09 | 3,48 | sugar transporter |
| HORVU5Hr1G081040 | 4,47 | 2,93 | TBC1 domain family member GTPase-activating protein |
| HORVU7Hr1G090100 | 2,90 | 3,73 | thiamine biosynthetic enzyme |
| HORVU2Hr1G015850 | 4,61 | 2,60 | TPR repeat containing protein; SGT-1-related |
| HORVU1Hr1G050560 | 4,79 | 2,60 | transcription factor MYC2 |
| HORVU1Hr1G055900 | 3,64 | 3,80 | transcriptional adaptor 2 (ADA2)-related |
| HORVU5Hr1G060460 | 3,05 | 4,87 | thehalose-6-phosphate synthase |
| HORVU2Hr1G029290 | 2,76 | 3,92 | trehalose-phosphatase [EC:3.1.3.12] |
| HORVU7Hr1G037140 | 4,21 | 6,39 | trehalose-phosphatase [EC:3.1.3.12] |
| HORVU7Hr1G118570 | 5,54 | 2,34 | unknown |
| HORVU5Hr1G084900 | 2,05 | 7,11 | unknown |
| HORVU7Hr1G101590 | 2,08 | 6,11 | unknown |
| HORVU2Hr1G027470 | 2,09 | 4,71 | unknown |
| HORVU2Hr1G023110 | 2,09 | 3,24 | unknown |
| HORVU5Hr1G022120 | 2,10 | 5,75 | unknown |
| HORVU5Hr1G061730 | 2,10 | 2,66 | unknown |
| HORVU7Hr1G051150 | 2,11 | 4,04 | unknown |
| HORVU1Hr1G024070 | 2,12 | 4,27 | unknown |
| HORVU5Hr1G069480 | 2,12 | 2,62 | unknown |
| HORVU2Hr1G094040 | 2,12 | 3,62 | unknown |
| HORVU4Hr1G002650 | 2,13 | 6,89 | unknown |
| HORVU3Hr1G072970 | 2,13 | 5,19 | unknown |
| HORVU6Hr1G077750 | 2,13 | 3,68 | unknown |
| HORVU7Hr1G085650 | 2,17 | 6,07 | unknown |
| HORVU6Hr1G073260 | 2,18 | 3,27 | unknown |
| HORVU5Hr1G023800 | 2,18 | 2,19 | unknown |
| HORVU1Hr1G057680 | 2,18 | 7,43 | unknown |
| HORVU4Hr1G052870 | 2,18 | 2,75 | unknown |
| HORVU1Hr1G029180 | 2,19 | 3,26 | unknown |
| HORVU6Hr1G053710 | 2,19 | 5,02 | unknown |
| HORVU7Hr1G019700 | 2,20 | 3,32 | unknown |
| HORVU2Hr1G017370 | 2,21 | 2,85 | unknown |
| HORVU1Hr1G057240 | 2,22 | 2,07 | unknown |
| HORVU2Hr1G099890 | 2,23 | 2,64 | unknown |
| HORVU2Hr1G036280 | 2,24 | 2,90 | unknown |
| HORVU4Hr1G072560 | 2,24 | 5,07 | unknown |
| HORVU4Hr1G050700 | 2,24 | 2,57 | unknown |
| HORVU2Hr1G112830 | 2,25 | 9,50 | unknown |
| HORVU1Hr1G047930 | 2,25 | 8,82 | unknown |
| HORVU4Hr1G051320 | 2,25 | 5,74 | unknown |
| HORVU1Hr1G071430 | 2,26 | 4,70 | unknown |
| HORVU4Hr1G066310 | 2,26 | 4,18 | unknown |
| HORVU5Hr1G047820 | 2,26 | 2,85 | unknown |
| HORVU2Hr1G017270 | 2,27 | 8,86 | unknown |
| HORVU6Hr1G031700 | 2,28 | 2,89 | unknown |
| HORVU5Hr1G024410 | 2,29 | 4,90 | unknown |
| HORVU3Hr1G083680 | 2,29 | 3,65 | unknown |
| HORVU3Hr1G074100 | 2,29 | 7,31 | unknown |
| HORVU4Hr1G002800 | 2,30 | 2,53 | unknown |
| HORVU7Hr1G108240 | 2,30 | 6,19 | unknown |
| HORVU4Hr1G060260 | 2,30 | 2,45 | unknown |
| HORVU3Hr1G032230 | 2,31 | 6,10 | unknown |
| HORVU4Hr1G020010 | 2,31 | 7,78 | unknown |
| HORVU2Hr1G017350 | 2,33 | 3,08 | unknown |
| HORVU5Hr1G044610 | 2,34 | 6,63 | unknown |
| HORVU7Hr1G075060 | 2,38 | 5,28 | unknown |
| HORVU5Hr1G059010 | 2,44 | 3,71 | unknown |
| HORVU4Hr1G014270 | 2,45 | 2,04 | unknown |
| HORVU5Hr1G084820 | 2,46 | 4,45 | unknown |
| HORVU4Hr1G043990 | 2,46 | 4,91 | unknown |
| HORVU5Hr1G075510 | 2,47 | 2,56 | unknown |
| HORVU5Hr1G007670 | 2,48 | 2,54 | unknown |
| HORVU5Hr1G064620 | 2,48 | 4,28 | unknown |
| HORVU4Hr1G075090 | 2,49 | 2,26 | unknown |
| HORVU7Hr1G075040 | 2,49 | 3,13 | unknown |
| HORVU4Hr1G063270 | 2,51 | 2,89 | unknown |
| HORVU4Hr1G081210 | 2,51 | 4,41 | unknown |
| HORVU6Hr1G064440 | 2,51 | 4,92 | unknown |
| HORVU4Hr1G067450 | 2,52 | 2,85 | unknown |
| HORVU7Hr1G038590 | 2,53 | 2,22 | unknown |
| HORVU3Hr1G116350 | 2,55 | 4,42 | unknown |
| HORVU4Hr1G065180 | 2,57 | 3,46 | unknown |
| HORVU2Hr1G064160 | 2,57 | 5,83 | unknown |
| HORVU1Hr1G015770 | 2,58 | 2,47 | unknown |
| HORVU1Hr1G080510 | 2,59 | 2,28 | unknown |
| HORVU7Hr1G042000 | 2,60 | 5,70 | unknown |
| HORVU3Hr1G081050 | 2,63 | 2,56 | unknown |
| HORVU1Hr1G058070 | 2,64 | 2,06 | unknown |
| HORVU3Hr1G030650 | 2,66 | 4,91 | unknown |
| HORVU3Hr1G049640 | 2,66 | 4,54 | unknown |
| HORVU1Hr1G088300 | 2,71 | 3,17 | unknown |
| HORVU3Hr1G026500 | 2,71 | 2,04 | unknown |
| HORVU1Hr1G072290 | 2,71 | 5,10 | unknown |
| HORVU2Hr1G111780 | 2,72 | 5,93 | unknown |
| HORVU7Hr1G108680 | 2,73 | 7,21 | unknown |
| HORVU5Hr1G118460 | 2,73 | 4,20 | unknown |
| HORVU6Hr1G028790 | 2,73 | 4,34 | unknown |
| HORVU7Hr1G072960 | 2,74 | 4,17 | unknown |
| HORVU1Hr1G007660 | 2,74 | 6,61 | unknown |
| HORVU4Hr1G012480 | 2,74 | 4,53 | unknown |
| HORVU4Hr1G071100 | 2,74 | 6,88 | unknown |
| HORVU4Hr1G027660 | 2,75 | 5,94 | unknown |
| HORVU7Hr1G055410 | 2,76 | 2,07 | unknown |
| HORVU3Hr1G019240 | 2,79 | 5,46 | unknown |
| HORVU2Hr1G036050 | 2,79 | 2,55 | unknown |
| HORVU2Hr1G098390 | 2,81 | 2,31 | unknown |
| HORVU5Hr1G103460 | 2,84 | 3,75 | unknown |
| HORVU4Hr1G011740 | 2,85 | 3,14 | unknown |
| HORVU7Hr1G047150 | 2,85 | 3,02 | unknown |
| HORVU3Hr1G080770 | 2,94 | 3,99 | unknown |
| HORVU2Hr1G015140 | 2,95 | 4,81 | unknown |
| HORVU4Hr1G010030 | 2,96 | 2,18 | unknown |
| HORVU7Hr1G110570 | 2,96 | 4,41 | unknown |
| HORVU7Hr1G085060 | 2,97 | 4,07 | unknown |
| HORVU6Hr1G031640 | 2,99 | 2,03 | unknown |
| HORVU5Hr1G114230 | 2,99 | 4,52 | unknown |
| HORVU2Hr1G124010 | 3,00 | 2,62 | unknown |
| HORVU6Hr1G018050 | 3,02 | 2,31 | unknown |
| HORVU1Hr1G005800 | 3,02 | 3,95 | unknown |
| HORVU5Hr1G125460 | 3,05 | 4,05 | unknown |
| HORVU4Hr1G013910 | 3,05 | 5,76 | unknown |
| HORVU1Hr1G082100 | 3,07 | 2,17 | unknown |
| HORVU4Hr1G078210 | 3,08 | 5,97 | unknown |
| HORVU5Hr1G064010 | 3,08 | 3,61 | unknown |
| HORVU2Hr1G094510 | 3,09 | 2,54 | unknown |
| HORVU7Hr1G017940 | 3,10 | 8,28 | unknown |
| HORVU2Hr1G098330 | 3,10 | 2,37 | unknown |
| HORVU4Hr1G052140 | 3,10 | 2,33 | unknown |
| HORVU5Hr1G061480 | 3,10 | 4,03 | unknown |
| HORVU7Hr1G007540 | 3,11 | 6,75 | unknown |
| HORVU5Hr1G067760 | 3,11 | 8,14 | unknown |
| HORVU1Hr1G046940 | 3,11 | 4,99 | unknown |
| HORVU0Hr1G002390 | 3,13 | 2,10 | unknown |
| HORVU3Hr1G085680 | 3,14 | 3,84 | unknown |
| HORVU3Hr1G011990 | 3,15 | 5,53 | unknown |
| HORVU1Hr1G089310 | 3,16 | 4,45 | unknown |
| HORVU5Hr1G068070 | 3,18 | 3,83 | unknown |
| HORVU4Hr1G060000 | 3,24 | 2,69 | unknown |
| HORVU5Hr1G018920 | 3,27 | 2,92 | unknown |
| HORVU2Hr1G064290 | 3,30 | 5,05 | unknown |
| HORVU5Hr1G082310 | 3,30 | 4,00 | unknown |
| HORVU6Hr1G078470 | 3,33 | 2,15 | unknown |
| HORVU2Hr1G091360 | 3,34 | 5,17 | unknown |
| HORVU7Hr1G098370 | 3,35 | 3,38 | unknown |
| HORVU2Hr1G080180 | 3,36 | 6,03 | unknown |
| HORVU2Hr1G035160 | 3,39 | 3,51 | unknown |
| HORVU5Hr1G081950 | 3,40 | 6,84 | unknown |
| HORVU6Hr1G035880 | 3,40 | 2,51 | unknown |
| HORVU3Hr1G022780 | 3,42 | 4,77 | unknown |
| HORVU7Hr1G108150 | 3,42 | 6,86 | unknown |
| HORVU6Hr1G067060 | 3,43 | 4,63 | unknown |
| HORVU3Hr1G058830 | 3,47 | 2,51 | unknown |
| HORVU2Hr1G032890 | 3,50 | 4,47 | unknown |
| HORVU2Hr1G113560 | 3,50 | 4,71 | unknown |
| HORVU3Hr1G074910 | 3,53 | 3,87 | unknown |
| HORVU1Hr1G092680 | 3,53 | 6,15 | unknown |
| HORVU2Hr1G008140 | 3,54 | 4,41 | unknown |
| HORVU1Hr1G059300 | 3,56 | 2,71 | unknown |
| HORVU2Hr1G092510 | 3,56 | 2,79 | unknown |
| HORVU5Hr1G068450 | 3,56 | 2,30 | unknown |
| HORVU3Hr1G090410 | 3,56 | 3,98 | unknown |
| HORVU5Hr1G060620 | 3,59 | 5,47 | unknown |
| HORVU4Hr1G083200 | 3,61 | 3,32 | unknown |
| HORVU2Hr1G083430 | 3,63 | 2,81 | unknown |
| HORVU7Hr1G109290 | 3,64 | 2,40 | unknown |
| HORVU7Hr1G033820 | 3,64 | 2,05 | unknown |
| HORVU5Hr1G036590 | 3,65 | 2,19 | unknown |
| HORVU4Hr1G022970 | 3,65 | 5,58 | unknown |
| HORVU5Hr1G122410 | 3,65 | 5,12 | unknown |
| HORVU5Hr1G046520 | 3,65 | 5,94 | unknown |
| HORVU5Hr1G013510 | 3,66 | 2,58 | unknown |
| HORVU4Hr1G073840 | 3,66 | 5,76 | unknown |
| HORVU4Hr1G075110 | 3,68 | 4,21 | unknown |
| HORVU1Hr1G080640 | 3,69 | 2,56 | unknown |
| HORVU7Hr1G050530 | 3,70 | 4,29 | unknown |
| HORVU3Hr1G031850 | 3,71 | 4,59 | unknown |
| HORVU2Hr1G105190 | 3,71 | 2,03 | unknown |
| HORVU3Hr1G005800 | 3,76 | 4,94 | unknown |
| HORVU1Hr1G077170 | 3,76 | 4,76 | unknown |
| HORVU6Hr1G089510 | 3,77 | 5,03 | unknown |
| HORVU2Hr1G079580 | 3,79 | 3,20 | unknown |
| HORVU6Hr1G077770 | 3,79 | 4,04 | unknown |
| HORVU1Hr1G059290 | 3,82 | 4,75 | unknown |
| HORVU3Hr1G068120 | 3,84 | 2,42 | unknown |
| HORVU1Hr1G008580 | 3,84 | 5,69 | unknown |
| HORVU4Hr1G089670 | 3,86 | 4,71 | unknown |
| HORVU4Hr1G000830 | 3,87 | 2,34 | unknown |
| HORVU6Hr1G025830 | 3,90 | 2,93 | unknown |
| HORVU1Hr1G064780 | 3,90 | 3,62 | unknown |
| HORVU5Hr1G077010 | 3,93 | 4,96 | unknown |
| HORVU2Hr1G004480 | 3,93 | 3,73 | unknown |
| HORVU6Hr1G034380 | 3,96 | 2,90 | unknown |
| HORVU7Hr1G056430 | 3,97 | 2,44 | unknown |
| HORVU5Hr1G005180 | 3,98 | 3,11 | unknown |
| HORVU1Hr1G026520 | 4,03 | 3,42 | unknown |
| HORVU7Hr1G085390 | 4,03 | 5,88 | unknown |
| HORVU2Hr1G034370 | 4,04 | 5,63 | unknown |
| HORVU6Hr1G080490 | 4,04 | 2,30 | unknown |
| HORVU2Hr1G083490 | 4,05 | 2,47 | unknown |
| HORVU2Hr1G032690 | 4,05 | 3,04 | unknown |
| HORVU1Hr1G050480 | 4,06 | 2,11 | unknown |
| HORVU3Hr1G016970 | 4,09 | 5,16 | unknown |
| HORVU6Hr1G058290 | 4,10 | 3,03 | unknown |
| HORVU2Hr1G078960 | 4,12 | 2,89 | unknown |
| HORVU4Hr1G078390 | 4,13 | 3,14 | unknown |
| HORVU4Hr1G021320 | 4,16 | 2,11 | unknown |
| HORVU2Hr1G085500 | 4,17 | 5,65 | unknown |
| HORVU5Hr1G024160 | 4,19 | 3,65 | unknown |
| HORVU5Hr1G080450 | 4,20 | 2,95 | unknown |
| HORVU5Hr1G045860 | 4,22 | 4,40 | unknown |
| HORVU5Hr1G021390 | 4,23 | 4,03 | unknown |
| HORVU5Hr1G063270 | 4,24 | 3,49 | unknown |
| HORVU2Hr1G098140 | 4,27 | 3,24 | unknown |
| HORVU5Hr1G081500 | 4,34 | 4,44 | unknown |
| HORVU3Hr1G086510 | 4,35 | 2,43 | unknown |
| HORVU1Hr1G046370 | 4,35 | 7,78 | unknown |
| HORVU6Hr1G088880 | 4,37 | 2,71 | unknown |
| HORVU2Hr1G122610 | 4,39 | 3,62 | unknown |
| HORVU5Hr1G117000 | 4,45 | 3,92 | unknown |
| HORVU7Hr1G083850 | 4,46 | 5,04 | unknown |
| HORVU4Hr1G088780 | 4,47 | 5,49 | unknown |
| HORVU7Hr1G036380 | 4,51 | 3,64 | unknown |
| HORVU5Hr1G115750 | 4,52 | 4,76 | unknown |
| HORVU2Hr1G094160 | 4,53 | 2,45 | unknown |
| HORVU3Hr1G022340 | 4,54 | 3,96 | unknown |
| HORVU7Hr1G081590 | 4,58 | 3,03 | unknown |
| HORVU4Hr1G063240 | 4,60 | 2,70 | unknown |
| HORVU3Hr1G022260 | 4,64 | 2,02 | unknown |
| HORVU4Hr1G063160 | 4,66 | 3,40 | unknown |
| HORVU7Hr1G096360 | 4,66 | 5,12 | unknown |
| HORVU0Hr1G006830 | 4,70 | 5,39 | unknown |
| HORVU6Hr1G053090 | 4,74 | 5,14 | unknown |
| HORVU3Hr1G036970 | 4,77 | 2,52 | unknown |
| HORVU7Hr1G007610 | 4,78 | 3,16 | unknown |
| HORVU5Hr1G115870 | 4,79 | 5,69 | unknown |
| HORVU5Hr1G121570 | 4,79 | 3,58 | unknown |
| HORVU7Hr1G091800 | 4,81 | 6,03 | unknown |
| HORVU3Hr1G051610 | 4,82 | 3,89 | unknown |
| HORVU1Hr1G087380 | 4,84 | 4,18 | unknown |
| HORVU2Hr1G123370 | 4,88 | 3,01 | unknown |
| HORVU7Hr1G043620 | 4,88 | 2,33 | unknown |
| HORVU3Hr1G062430 | 4,89 | 3,43 | unknown |
| HORVU5Hr1G065630 | 4,91 | 4,66 | unknown |
| HORVU0Hr1G016380 | 4,93 | 3,52 | unknown |
| HORVU7Hr1G092710 | 5,00 | 2,19 | unknown |
| HORVU0Hr1G007340 | 5,06 | 3,83 | unknown |
| HORVU2Hr1G065750 | 5,07 | 4,27 | unknown |
| HORVU6Hr1G077780 | 5,07 | 2,05 | unknown |
| HORVU4Hr1G063690 | 5,13 | 3,15 | unknown |
| HORVU3Hr1G035650 | 5,14 | 4,46 | unknown |
| HORVU4Hr1G013460 | 5,20 | 4,26 | unknown |
| HORVU2Hr1G035020 | 5,22 | 4,67 | unknown |
| HORVU5Hr1G111460 | 5,26 | 3,34 | unknown |
| HORVU2Hr1G112120 | 5,27 | 4,48 | unknown |
| HORVU4Hr1G060280 | 5,28 | 3,85 | unknown |
| HORVU5Hr1G084700 | 5,32 | 4,41 | unknown |
| HORVU3Hr1G060500 | 5,33 | 4,40 | unknown |
| HORVU7Hr1G098010 | 5,34 | 2,95 | unknown |
| HORVU0Hr1G000780 | 5,35 | 4,12 | unknown |
| HORVU2Hr1G036120 | 5,38 | 3,08 | unknown |
| HORVU7Hr1G100090 | 5,40 | 6,73 | unknown |
| HORVU2Hr1G065000 | 5,43 | 5,64 | unknown |
| HORVU2Hr1G015150 | 5,45 | 3,98 | unknown |
| HORVU0Hr1G023200 | 5,48 | 2,15 | unknown |
| HORVU2Hr1G034670 | 5,49 | 4,38 | unknown |
| HORVU2Hr1G035870 | 5,49 | 3,87 | unknown |
| HORVU3Hr1G043300 | 5,55 | 2,36 | unknown |
| HORVU5Hr1G045640 | 5,62 | 3,17 | unknown |
| HORVU7Hr1G093940 | 5,69 | 3,92 | unknown |
| HORVU6Hr1G075270 | 5,93 | 2,60 | unknown |
| HORVU7Hr1G105150 | 5,98 | 2,86 | unknown |
| HORVU7Hr1G089160 | 5,98 | 2,63 | unknown |
| HORVU2Hr1G122580 | 6,14 | 4,05 | unknown |
| HORVU3Hr1G106850 | 6,16 | 2,65 | unknown |
| HORVU2Hr1G042520 | 6,41 | 2,56 | unknown |
| HORVU1Hr1G065150 | 6,52 | 2,58 | unknown |
| HORVU2Hr1G028670 | 6,66 | 2,64 | unknown |
| HORVU3Hr1G016860 | 6,78 | 2,71 | unknown |
| HORVU2Hr1G119500 | 6,80 | 2,69 | unknown |
| HORVU6Hr1G021870 | 7,08 | 3,43 | unknown |
| HORVU5Hr1G071720 | 8,14 | 2,64 | unknown |
| HORVU5Hr1G013290 | 2,33 | 3,78 | von willebrand factor, type A domain containing |
| HORVU6Hr1G066870 | 2,34 | 5,90 | WDSAM1 protein |
| HORVU4Hr1G064070 | 2,44 | 3,46 | WDSAM1 protein |
| HORVU7Hr1G073100 | 2,61 | 2,93 | WDSAM1 protein |
| HORVU2Hr1G074130 | 4,38 | 2,37 | WDSAM1 protein |
| HORVU7Hr1G080950 | 5,82 | 3,39 | WRKY transcription factor 22 |
| HORVU1Hr1G070250 | 5,12 | 3,49 | WRKY transcription factor 33 |
| HORVU0Hr1G006800 | 2,01 | 3,48 | ZINC FINGER five domain containing protein |
| HORVU3Hr1G069070 | 2,12 | 6,99 | ZINC FINGER five domain containing protein |
| HORVU6Hr1G035210 | 2,47 | 5,59 | ZINC FINGER five domain containing protein |
| HORVU3Hr1G010920 | 2,57 | 4,58 | ZINC FINGER five domain containing protein |
| HORVU1Hr1G023510 | 2,82 | 2,40 | ZINC FINGER five domain containing protein |
| HORVU7Hr1G038400 | 3,50 | 3,64 | ZINC FINGER five domain containing protein |
| HORVU1Hr1G084390 | 3,64 | 6,25 | ZINC FINGER five domain containing protein |
| HORVU5Hr1G125000 | 5,80 | 5,94 | ZINC FINGER five domain containing protein |
| HORVU6Hr1G076660 | 6,40 | 2,09 | ZINC FINGER five domain containing protein |
