# Supplementary material for: Differential Expression Profiling of Microspores During the Early Stages of Isolated Microspore Culture Using the Responsive Barley Cultivar Gobernadora
Source: G3 (Bethesda). 2018 Mar 12;8(5):1603–14. doi: 10.1534/g3.118.200208 (PMC5940152; doi:10.1534/g3.118.200208)
Supplement: Supplementary file 5 [file 1603TableS5.docx]

Supplementary Table 5: Gene functional annotation for genes in cluster 5

| Gene stable ID (cluster 5) | logFC D2-D0 | logFC D5-D2 | Gene function annotation |
| --- | --- | --- | --- |
| HORVU5Hr1G068330 | 4,32 | 1,64 | (+)-abscisic acid 8'-hydroxylase [EC:1.14.13.93] |
| HORVU2Hr1G060010 | 2,36 | -1,55 | (S)-2-hydroxy-acid oxidase [EC:1.1.3.15] |
| HORVU2Hr1G032330 | 7,20 | 1,52 | [heparan sulfate]-glucosamine 3-sulfotransferase 1 [EC:2.8.2.23] |
| HORVU4Hr1G044140 | 2,23 | -0,52 | 1,2-diacylglycerol 3-beta-galactosyltransferase [EC:2.4.1.46] |
| HORVU1Hr1G083380 | 3,34 | 1,06 | 1,2-dihydroxy-3-keto-5-methylthiopentene dioxygenase [EC:1.13.11.53 1.13.11.54] |
| HORVU1Hr1G054430 | 2,25 | 1,85 | 2-deoxyglucose-6-phosphate phosphatase 2 |
| HORVU4Hr1G007340 | 2,25 | -0,27 | 26S proteasome regulatory subunit, ATPase 3, interacting protein |
| HORVU3Hr1G006910 | 4,34 | 1,22 | 3-oxoacyl-[acyl-carrier protein] reductase [EC:1.1.1.100] |
| HORVU5Hr1G052320 | 2,87 | -0,32 | 4-nitrophenylphosphatase-related |
| HORVU4Hr1G022630 | 4,16 | -0,69 | 5'-AMP-activated protein kinase, catalytic alpha subunit [EC:2.7.11.11] |
| HORVU3Hr1G030770 | 5,31 | -0,39 | AAA ATPase |
| HORVU6Hr1G053910 | 3,23 | 1,52 | actin |
| HORVU0Hr1G017620 | 2,22 | -0,27 | actin related protein 2/3 complex, subunit 1A/1B |
| HORVU3Hr1G083380 | 4,67 | 0,92 | actin, other eukaryote |
| HORVU3Hr1G091320 | 8,31 | -0,73 | acyl-[acyl-carrier-protein] desaturase [EC:1.14.19.2] |
| HORVU5Hr1G057520 | 2,64 | 0,68 | agmatine deiminase [EC:3.5.3.12] |
| HORVU1Hr1G018540 | 3,25 | 1,17 | alanine transaminase [EC:2.6.1.2] |
| HORVU4Hr1G016770 | 6,68 | -1,89 | alcohol dehydrogenase [EC:1.1.1.1] |
| HORVU2Hr1G020900 | 4,21 | -0,58 | alcohol dehydrogenase related |
| HORVU0Hr1G010220 | 4,30 | -0,94 | alcohol dehydrogenase related |
| HORVU4Hr1G016810 | 4,76 | -1,07 | alcohol dehydrogenase related |
| HORVU3Hr1G085780 | 2,06 | -1,24 | aldo/keto reductase |
| HORVU3Hr1G054640 | 2,38 | 0,49 | aldo/keto reductase |
| HORVU2Hr1G080140 | 2,05 | 1,11 | aldose 1-epimerase [EC:5.1.3.3] |
| HORVU5Hr1G111850 | 2,00 | 1,37 | alkylated DNA repain protein ALKB-related |
| HORVU7Hr1G108960 | 2,52 | 0,23 | allantoate deiminase [EC:3.5.3.9] |
| HORVU3Hr1G067620 | 3,46 | 0,30 | alpha-amylase |
| HORVU5Hr1G068350 | 5,48 | 1,34 | alpha-amylase [EC:3.2.1.1] |
| HORVU5Hr1G113880 | 2,10 | 0,01 | alpha-mannosidase [EC:3.2.1.24] |
| HORVU5Hr1G045150 | 2,05 | 0,28 | alpha-N-arabinofuranosidase [EC:3.2.1.55] |
| HORVU4Hr1G052170 | 3,00 | -1,32 | alpha-N-arabinofuranosidase [EC:3.2.1.55] |
| HORVU2Hr1G032400 | 2,06 | -0,44 | alpha/beta hydrolase fold-containing protein |
| HORVU7Hr1G083370 | 2,14 | -0,56 | alpha/beta hydrolase fold-containing protein |
| HORVU5Hr1G012890 | 2,17 | 1,44 | alpha/beta hydrolase fold-containing protein |
| HORVU7Hr1G045640 | 5,66 | -1,76 | alpha/beta hydrolase fold-containing protein |
| HORVU3Hr1G098360 | 5,97 | 0,01 | alpha/beta hydrolase fold-containing protein |
| HORVU6Hr1G068910 | 8,09 | -1,95 | alpha/beta hydrolase fold-containing protein |
| HORVU3Hr1G088210 | 4,26 | -0,46 | amidase |
| HORVU2Hr1G028980 | 3,64 | -0,93 | amine oxidase |
| HORVU4Hr1G027180 | 4,59 | 0,78 | amino acid transporter |
| HORVU7Hr1G088510 | 4,71 | 1,49 | amino acid transporter |
| HORVU7Hr1G050270 | 2,95 | 1,69 | AN1-type zinc finger protein |
| HORVU4Hr1G003430 | 3,20 | 0,17 | ancient conserved domain protein-related |
| HORVU3Hr1G033250 | 2,61 | -0,98 | ankyrin repeat and protein kinase domain-containing protein |
| HORVU4Hr1G074130 | 6,05 | 0,01 | annexin |
| HORVU4Hr1G019140 | 2,08 | 0,25 | AP-4 complex subunit sigma-1 |
| HORVU3Hr1G031680 | 3,17 | 1,75 | aquaporin TIP |
| HORVU2Hr1G097780 | 5,54 | 0,27 | aquaporin TIP |
| HORVU1Hr1G043890 | 5,58 | 1,08 | aquaporin TIP |
| HORVU2Hr1G005320 | 3,20 | 1,01 | arginase [EC:3.5.3.1] |
| HORVU5Hr1G059900 | 2,12 | -0,41 | arsenite inducuble RNA associated protein AIP-1-related |
| HORVU6Hr1G066980 | 2,42 | 1,41 | aspartyl proteases |
| HORVU0Hr1G021860 | 2,50 | 1,03 | aspartyl proteases |
| HORVU7Hr1G042490 | 3,61 | 0,07 | aspartyl proteases |
| HORVU3Hr1G100350 | 5,05 | 1,18 | aspartyl proteases |
| HORVU7Hr1G116780 | 8,15 | 1,27 | aspartyl proteases |
| HORVU5Hr1G106850 | 2,47 | -1,20 | ATP-binding cassette transporter |
| HORVU7Hr1G092510 | 3,92 | -1,53 | ATP-binding cassette transporter |
| HORVU5Hr1G124650 | 3,97 | -0,81 | ATP-binding cassette transporter |
| HORVU7Hr1G085680 | 3,36 | 0,97 | ATP-binding cassette transporter subfamily A (ABCA) |
| HORVU3Hr1G065320 | 2,62 | -1,65 | ATP-binding cassette, subfamily B (MDR/TAP) |
| HORVU3Hr1G065390 | 3,66 | 0,65 | ATP-binding cassette, subfamily B (MDR/TAP) |
| HORVU5Hr1G048300 | 2,57 | 0,83 | ATP-dependent Clp protease |
| HORVU5Hr1G081860 | 2,12 | 0,03 | ATP-dependent Clp protease ATP-binding subunit ClpC |
| HORVU1Hr1G094480 | 3,07 | 1,97 | ATP-dependent Clp protease [EC:3.4.21.92] |
| HORVU1Hr1G091570 | 2,03 | 0,26 | auxilin/cyclin G-associated kinase-related |
| HORVU2Hr1G019970 | 2,16 | -1,73 | auxin responsive GH3 gene family |
| HORVU0Hr1G023140 | 2,11 | 0,82 | basic amino acid/polyamine antiporter, APA |
| HORVU1Hr1G093760 | 2,76 | 1,72 | BCS1 AAA-type ATPase |
| HORVU3Hr1G052880 | 2,93 | 1,89 | BCS1 AAA-type ATPase |
| HORVU3Hr1G057540 | 3,26 | 0,16 | BCS1 AAA-type ATPase |
| HORVU2Hr1G046330 | 7,23 | -0,70 | BCS1 AAA-type ATPase |
| HORVU5Hr1G083170 | 2,22 | -0,37 | beta catenin-related armadillo repeat-containing |
| HORVU3Hr1G109540 | 6,34 | 0,56 | beta catenin-related armadillo repeat-containing |
| HORVU1Hr1G053070 | 5,83 | -0,45 | beta lactamase domain |
| HORVU6Hr1G026680 | 3,14 | 0,26 | beta-1,3-N-acetylglucosaminyltransferase |
| HORVU6Hr1G083140 | 7,48 | -0,84 | beta-1,3-N-acetylglucosaminyltransferase |
| HORVU6Hr1G053550 | 2,16 | 0,46 | beta-1,4-mannosyl-glycoprotein beta-1,4-N-acetylglucosaminyltransferase [EC:2.4.1.144] |
| HORVU2Hr1G072400 | 3,47 | 1,84 | beta-carotene 15,15'-monooxygenase [EC:1.14.99.36] |
| HORVU1Hr1G047030 | 3,60 | -1,41 | beta-carotene 15,15'-monooxygenase [EC:1.14.99.36] |
| HORVU5Hr1G096940 | 6,34 | 0,55 | beta-carotene 15,15'-monooxygenase [EC:1.14.99.36] |
| HORVU5Hr1G096930 | 6,73 | 1,79 | beta-carotene 15,15'-monooxygenase [EC:1.14.99.36] |
| HORVU6Hr1G059730 | 3,11 | 1,96 | beta-cyano-L-alanine hydratase/nitrilase [EC:3.5.5.1 3.5.5.4] |
| HORVU3Hr1G005350 | 5,21 | 1,22 | beta-fructofuranosidase [EC:3.2.1.26] |
| HORVU1Hr1G073280 | 3,96 | 0,77 | beta-glucosidase [EC:3.2.1.21] |
| HORVU3Hr1G075310 | 4,16 | 0,62 | beta-glucosidase [EC:3.2.1.21] |
| HORVU4Hr1G068420 | 4,46 | 1,25 | beta-glucosidase [EC:3.2.1.21] |
| HORVU2Hr1G089050 | 4,68 | -1,66 | beta-glucosidase [EC:3.2.1.21] |
| HORVU3Hr1G109930 | 3,04 | -0,90 | beta-mannan synthase [EC:2.4.1.32] |
| HORVU7Hr1G076680 | 2,18 | -0,60 | bile acid beta-glucosidase-related |
| HORVU1Hr1G038870 | 4,18 | 1,07 | calcium-activated chloride channel regulator |
| HORVU1Hr1G029850 | 2,64 | 0,75 | calcium-binding protein CML |
| HORVU5Hr1G048000 | 3,75 | -0,92 | calcium-binding protein CML |
| HORVU5Hr1G032550 | 2,33 | -0,64 | calcium-dependent protein kinase [EC:2.7.11.1] |
| HORVU5Hr1G110900 | 3,41 | 1,97 | calcium-dependent protein kinase [EC:2.7.11.1] |
| HORVU4Hr1G026640 | 4,14 | -1,37 | calcium-dependent protein kinase [EC:2.7.11.1] |
| HORVU1Hr1G092710 | 4,28 | 0,49 | calcium-dependent protein kinase [EC:2.7.11.1] |
| HORVU7Hr1G029930 | 2,22 | -1,59 | callose synthase [EC:2.4.1.-] |
| HORVU5Hr1G054510 | 3,79 | -0,93 | camp-response element binding protein-related |
| HORVU4Hr1G021720 | 3,86 | 1,61 | camp-response element binding protein-related |
| HORVU3Hr1G057090 | 3,70 | 1,91 | carbonic anhydrase [EC:4.2.1.1] |
| HORVU3Hr1G031560 | 2,01 | -0,06 | casein kinase-related |
| HORVU4Hr1G010300 | 3,77 | 0,29 | cathepsin B [EC:3.4.22.1] |
| HORVU1Hr1G090910 | 3,17 | -1,49 | CCCH zinc finger/TIS11-related |
| HORVU3Hr1G059130 | 2,23 | 0,53 | cell division protein kinase |
| HORVU5Hr1G118270 | 2,52 | 1,01 | cellulose synthase A [EC:2.4.1.12] |
| HORVU5Hr1G064230 | 2,80 | 1,85 | cellulose synthase A [EC:2.4.1.12] |
| HORVU1Hr1G039250 | 4,69 | 0,48 | cellulose synthase A [EC:2.4.1.12] |
| HORVU2Hr1G088180 | 4,07 | 0,40 | centaurin/ARF |
| HORVU2Hr1G092250 | 2,01 | -1,74 | centromeric protein E |
| HORVU0Hr1G016310 | 5,05 | 1,23 | chitinase [EC:3.2.1.14] |
| HORVU0Hr1G007360 | 2,93 | 1,15 | chlorophyllide a oxygenase [EC:1.13.12.14] |
| HORVU3Hr1G108750 | 8,27 | 1,99 | chromate reductase |
| HORVU2Hr1G013450 | 3,64 | 0,24 | circadian protein CLOCK/ARNT/BMAL/PAS |
| HORVU2Hr1G096890 | 2,73 | 1,27 | cis-zeatin O-glucosyltransferase [EC:2.4.1.215] |
| HORVU5Hr1G088510 | 3,98 | 0,71 | cleavage and polyadenylation specificity factor subunit 3 [EC:3.1.27.-] |
| HORVU1Hr1G062190 | 2,09 | -0,85 | cohesin complex subunit SCC1 |
| HORVU7Hr1G085120 | 7,79 | -0,70 | coniferyl-aldehyde dehydrogenase [EC:1.2.1.68] |
| HORVU5Hr1G109720 | 2,85 | 0,58 | COP9 signalosome complex subunit 5 |
| HORVU2Hr1G070140 | 2,31 | 0,81 | copper transport protein ATOX1-related |
| HORVU6Hr1G009000 | 5,95 | 1,71 | copper transport protein ATOX1-related |
| HORVU6Hr1G052250 | 3,17 | -0,17 | CTL transporter |
| HORVU2Hr1G114940 | 4,79 | 0,04 | cyclic nucleotide gated channel, other eukaryote |
| HORVU5Hr1G068770 | 3,32 | -1,77 | cyclin-dependant kinase inhibitor 1 |
| HORVU0Hr1G003280 | 2,75 | 0,90 | cycline |
| HORVU4Hr1G066360 | 4,50 | -1,35 | cycline |
| HORVU1Hr1G094490 | 4,29 | 0,03 | cysteamine dioxygenase [EC:1.13.11.19] |
| HORVU3Hr1G069210 | 9,29 | -1,12 | cysteine desulfurylase |
| HORVU7Hr1G033620 | 6,98 | -1,14 | cysteine-rich secretory protein related |
| HORVU7Hr1G033530 | 7,80 | 0,04 | cysteine-rich secretory protein related |
| HORVU1Hr1G038700 | 2,35 | -0,83 | cysteinyl-tRNA synthetase [EC:6.1.1.16] |
| HORVU1Hr1G057860 | 2,08 | -0,89 | cytokinin dehydrogenase [EC:1.5.99.12] |
| HORVU3Hr1G075920 | 5,36 | -1,63 | cytokinin dehydrogenase [EC:1.5.99.12] |
| HORVU7Hr1G068230 | 2,36 | -1,24 | diacylglycerol kinase [EC:2.7.1.107] |
| HORVU5Hr1G020280 | 5,09 | -0,76 | diacylglycerol kinase [EC:2.7.1.107] |
| HORVU7Hr1G025990 | 2,12 | 0,49 | dienelactone hydrolase |
| HORVU0Hr1G012990 | 5,02 | 0,76 | dimethylaniline monooxygenase |
| HORVU7Hr1G041500 | 2,70 | 1,22 | diphosphoinositol-polyphosphate diphosphatase [EC:3.6.1.52] |
| HORVU7Hr1G117100 | 2,52 | 0,73 | DNA (cytosine-5-)-methyltransferase [EC:2.1.1.37] |
| HORVU5Hr1G020430 | 2,02 | 0,36 | DNA polymerase III subunit epsilon |
| HORVU6Hr1G082430 | 2,09 | -1,56 | DNA-directed RNA polymerase |
| HORVU5Hr1G114400 | 3,51 | -0,08 | DNA-directed RNA polymerase |
| HORVU6Hr1G067130 | 2,01 | 0,09 | DNAJ homolog subfamily C member |
| HORVU1Hr1G056020 | 2,14 | -0,05 | DNAJ homolog subfamily C member |
| HORVU7Hr1G033430 | 5,43 | -0,97 | DNAJ homolog subfamily C member |
| HORVU4Hr1G087120 | 2,78 | -0,33 | E3 ubiquitin-protein ligase SIAH1 [EC:6.3.2.19] |
| HORVU5Hr1G123390 | 2,19 | -1,71 | E3 ubiquitin-protein ligse UHRF-related |
| HORVU3Hr1G017630 | 2,70 | 1,92 | EBNA2 binding protein P100 |
| HORVU7Hr1G027770 | 3,61 | 1,03 | EF-hand calcium-binding domain containing protein |
| HORVU3Hr1G109170 | 4,59 | 1,07 | EF-hand calcium-binding domain containing protein |
| HORVU5Hr1G111520 | 5,53 | -0,67 | EF-hand calcium-binding domain containing protein |
| HORVU5Hr1G082460 | 3,33 | -0,12 | endo-1,4-beta-glucanase |
| HORVU6Hr1G081500 | 7,28 | -0,31 | endoglucanase [EC:3.2.1.4] |
| HORVU1Hr1G058880 | 2,87 | -0,46 | enoyl-CoA hydratase/3-hydroxyacyl-CoA dehydrogenase [EC:4.2.1.17 1.1.1.35 1.1.1.211] |
| HORVU7Hr1G032750 | 2,87 | 0,39 | EREBP-like factor |
| HORVU5Hr1G062940 | 3,09 | 1,99 | EREBP-like factor |
| HORVU4Hr1G010650 | 4,66 | -1,81 | ERO1-like protein alpha [EC:1.8.4.-] |
| HORVU1Hr1G025960 | 2,05 | -0,06 | euchromatic histone-lysine N-methyltransferase [EC:2.1.1.43] |
| HORVU4Hr1G074120 | 2,15 | 0,16 | euchromatic histone-lysine N-methyltransferase [EC:2.1.1.43] |
| HORVU6Hr1G036840 | 2,60 | 0,07 | eukaryotic translation initiation factor 2C |
| HORVU2Hr1G067350 | 2,69 | 1,35 | exocyst complexe protein EXO70 |
| HORVU2Hr1G067340 | 2,96 | 1,69 | exocyst complexe protein EXO70 |
| HORVU1Hr1G038500 | 4,62 | 0,52 | exotosin (heparan sulfate glycosyltransferase)-related |
| HORVU2Hr1G052430 | 2,19 | 0,51 | extended synaptotagmin-related |
| HORVU2Hr1G082480 | 2,95 | 0,89 | extended synaptotagmin-related |
| HORVU3Hr1G086220 | 7,31 | 0,94 | extended synaptotagmin-related |
| HORVU4Hr1G057200 | 2,72 | 1,57 | extracellular signal-regulated kinase 1/2 [EC:2.7.11.24] |
| HORVU4Hr1G020480 | 3,61 | -0,58 | F-box and leucine-rich repeat protein 2/20 |
| HORVU4Hr1G086000 | 3,09 | 0,34 | F-BOX and WD40 domain protein |
| HORVU3Hr1G084210 | 8,95 | 0,94 | ferredoxin |
| HORVU4Hr1G015330 | 2,48 | 0,89 | ferredoxin-related |
| HORVU3Hr1G042140 | 2,36 | 0,74 | fimbrin/plastin |
| HORVU0Hr1G015390 | 2,42 | -0,13 | formin-related |
| HORVU2Hr1G035230 | 4,25 | 1,67 | formin-related |
| HORVU0Hr1G000810 | 2,03 | -0,37 | GAG-POL-related retrotransposon |
| HORVU2Hr1G053920 | 2,49 | -1,47 | GAG/POL/ENV polyprotein |
| HORVU2Hr1G113350 | 4,86 | 0,95 | galactosylgalactosylxylosylprotein 3-beta-glucuronosyltransferase (BETA-1,3-glucuronosyltransferase) |
| HORVU3Hr1G072810 | 6,85 | 1,90 | gibberellin 2-oxidase [EC:1.14.11.13] |
| HORVU2Hr1G111520 | 2,22 | -1,29 | glucose-6-phosphate 1-epimerase [EC:5.1.3.15] |
| HORVU5Hr1G056390 | 4,63 | -1,11 | glucose-6-phosphate 1-epimerase [EC:5.1.3.15] |
| HORVU5Hr1G047150 | 3,44 | 0,16 | glucosyl/glucuronosyl transferases |
| HORVU7Hr1G085270 | 3,65 | 1,47 | glucosyl/glucuronosyl transferases |
| HORVU2Hr1G066780 | 4,22 | 0,81 | glucosyl/glucuronosyl transferases |
| HORVU7Hr1G038510 | 4,24 | 1,80 | glucosyl/glucuronosyl transferases |
| HORVU2Hr1G012220 | 4,61 | -0,93 | glucosyl/glucuronosyl transferases |
| HORVU2Hr1G012280 | 4,69 | -1,68 | glucosyl/glucuronosyl transferases |
| HORVU2Hr1G108330 | 5,46 | 1,21 | glucosyl/glucuronosyl transferases |
| HORVU5Hr1G096320 | 5,86 | -0,38 | glucosyl/glucuronosyl transferases |
| HORVU5Hr1G104580 | 6,20 | -0,13 | glucosyl/glucuronosyl transferases |
| HORVU3Hr1G065420 | 6,69 | 0,21 | glucosyl/glucuronosyl transferases |
| HORVU3Hr1G099530 | 7,96 | -1,62 | glucosyl/glucuronosyl transferases |
| HORVU3Hr1G070550 | 3,50 | 1,50 | glutamate carboxypeptidase II [EC:3.4.17.21] |
| HORVU3Hr1G003050 | 4,26 | -1,27 | glutamate decarboxylase [EC:4.1.1.15] |
| HORVU4Hr1G064790 | 8,32 | -1,89 | glutamate decarboxylase [EC:4.1.1.15] |
| HORVU6Hr1G009520 | 2,07 | -0,38 | glutamate receptor, ionotropic, other eukaryote |
| HORVU2Hr1G104920 | 2,28 | -0,87 | glutamate receptor, ionotropic, other eukaryote |
| HORVU6Hr1G015450 | 2,08 | -0,02 | glutamine amidotransferase [EC:2.6.-.-] |
| HORVU4Hr1G075840 | 2,23 | -1,74 | glutamine amidotransferase [EC:2.6.-.-] |
| HORVU2Hr1G115200 | 2,71 | -1,53 | glutaredoxin |
| HORVU1Hr1G087600 | 4,26 | 1,48 | glutaredoxin |
| HORVU5Hr1G058000 | 2,37 | -1,34 | glutathione S-transferase [EC:2.5.1.18] |
| HORVU3Hr1G107350 | 2,97 | -1,61 | glutathione S-transferase [EC:2.5.1.18] |
| HORVU5Hr1G103420 | 3,21 | 1,46 | glutathione S-transferase [EC:2.5.1.18] |
| HORVU1Hr1G049250 | 4,71 | -0,65 | glutathione S-transferase [EC:2.5.1.18] |
| HORVU3Hr1G095670 | 5,91 | 0,90 | glutathione S-transferase [EC:2.5.1.18] |
| HORVU1Hr1G049190 | 6,23 | 0,31 | glutathione S-transferase [EC:2.5.1.18] |
| HORVU1Hr1G064890 | 7,24 | 0,25 | glutathione S-transferase [EC:2.5.1.18] |
| HORVU1Hr1G021170 | 8,18 | -0,99 | glutathione S-transferase [EC:2.5.1.18] |
| HORVU4Hr1G057910 | 3,24 | -0,60 | glutathione S-transferase [EC:2.5.1.18] |
| HORVU7Hr1G049960 | 2,57 | 1,09 | glycerophosphoryl diester phosphodiesterase |
| HORVU1Hr1G016490 | 2,30 | -0,83 | glycogen synthase kinase 3 beta [EC:2.7.11.26] |
| HORVU4Hr1G052450 | 4,10 | 1,37 | glycogenin |
| HORVU4Hr1G007360 | 2,17 | -1,74 | glycolipid transfert protein |
| HORVU2Hr1G106410 | 2,44 | -0,30 | glycosyltransferase |
| HORVU1Hr1G080720 | 2,82 | 1,21 | glycosyltransferase |
| HORVU7Hr1G048470 | 2,86 | 1,28 | glycosyltransferase |
| HORVU1Hr1G063410 | 6,42 | 1,05 | glycosyltransferase |
| HORVU1Hr1G021890 | 4,42 | -0,96 | glycosyltransferase 14 family |
| HORVU4Hr1G059270 | 4,74 | -1,54 | glyoxalase family protein |
| HORVU2Hr1G076850 | 2,85 | 1,37 | GTPase, IMAP family member-related |
| HORVU2Hr1G031740 | 2,11 | 1,92 | guanosine-3',5'-BIS(diphosphate) 3’-pyrophosphohydrolase |
| HORVU7Hr1G093440 | 2,51 | 0,43 | guanyl-nucleotide exchange factor |
| HORVU7Hr1G047740 | 5,25 | 1,29 | gulonolactone oxidase |
| HORVU7Hr1G082860 | 3,79 | -1,04 | helicase-related |
| HORVU5Hr1G094490 | 4,05 | 0,04 | hemoglobin; BAX inhibitor-related |
| HORVU1Hr1G075830 | 2,18 | -1,37 | histidine-containing phosphotransfer protein |
| HORVU5Hr1G087870 | 2,33 | -1,12 | histone H4 |
| HORVU3Hr1G083480 | 2,84 | -1,69 | histone-lysine N-methyltransferase SETD2 [EC:2.1.1.43] |
| HORVU1Hr1G066650 | 2,06 | -0,59 | HMW glutenin subunit-related |
| HORVU4Hr1G090030 | 2,04 | 1,54 | homeobox-leucine zipper protein |
| HORVU5Hr1G070260 | 6,52 | 1,02 | homeobox-leucine zipper protein |
| HORVU7Hr1G110990 | 2,33 | 1,62 | homogenitisate phytyltransferase |
| HORVU2Hr1G077710 | 2,98 | 0,75 | HSP20 family protein |
| HORVU4Hr1G063350 | 3,34 | 1,34 | HSP20 family protein |
| HORVU4Hr1G015170 | 3,58 | 0,32 | HSP20 family protein |
| HORVU0Hr1G020420 | 3,63 | 1,00 | HSP20 family protein |
| HORVU3Hr1G007500 | 3,63 | 0,79 | HSP20 family protein |
| HORVU4Hr1G060760 | 3,77 | 1,18 | HSP20 family protein |
| HORVU3Hr1G007380 | 3,82 | 0,86 | HSP20 family protein |
| HORVU2Hr1G120170 | 4,37 | 0,96 | HSP20 family protein |
| HORVU3Hr1G006530 | 4,73 | 1,13 | HSP20 family protein |
| HORVU4Hr1G059260 | 2,88 | 0,82 | heat shock 70kDa protein 1/8 |
| HORVU1Hr1G081300 | 6,20 | -0,43 | heat shock transcription factor, other eukaryote |
| HORVU5Hr1G040210 | 2,48 | -0,27 | HVA22-like protein |
| HORVU7Hr1G045630 | 5,91 | -1,07 | HVA22-like protein |
| HORVU2Hr1G073410 | 6,40 | -0,54 | HVA22-like protein |
| HORVU3Hr1G004850 | 2,59 | 1,53 | hydroquinone glucosyltransferase [EC:2.4.1.218] |
| HORVU2Hr1G071750 | 3,22 | 1,96 | inhibitor of apoptosis |
| HORVU1Hr1G054930 | 3,82 | -0,03 | inhibitor of apoptosis |
| HORVU1Hr1G060960 | 2,09 | -1,37 | inosine-uridine preferring nucleoside hydrolase |
| HORVU5Hr1G061150 | 6,23 | 1,53 | interleukin-1 receptor-associated kinase 1 [EC:2.7.11.1] |
| HORVU7Hr1G031700 | 2,11 | -0,86 | ionotropic glutmate receptor |
| HORVU5Hr1G063430 | 3,93 | 0,00 | ionotropic glutmate receptor |
| HORVU1Hr1G089730 | 2,51 | -0,39 | iron-sulfur cluster assembly enzyme (NIFU homolog) |
| HORVU5Hr1G070160 | 3,02 | -0,96 | isoamylase [EC:3.2.1.68] |
| HORVU4Hr1G076840 | 3,08 | 0,89 | jasmonate ZIM domain-containing protein |
| HORVU4Hr1G083000 | 3,12 | 0,49 | KELCH-related |
| HORVU5Hr1G050510 | 2,74 | 0,17 | kinesin family member 4/7/21/27 |
| HORVU2Hr1G009940 | 6,13 | -0,45 | L-ascorbate peroxidase [EC:1.11.1.11] |
| HORVU6Hr1G015500 | 3,73 | -0,28 | L-lactate dehydrogenase [EC:1.1.1.27] |
| HORVU7Hr1G109650 | 2,80 | -1,60 | large subunit ribosomal protein L40e |
| HORVU2Hr1G099870 | 6,61 | 1,67 | Late embryogenesis abundant LEA-related |
| HORVU6Hr1G028220 | 2,07 | 1,43 | Leucine-rich repeat-containing protein |
| HORVU7Hr1G046370 | 2,17 | -0,49 | Leucine-rich repeat-containing protein |
| HORVU7Hr1G055790 | 2,23 | -1,07 | Leucine-rich repeat-containing protein |
| HORVU7Hr1G093710 | 2,24 | -0,71 | Leucine-rich repeat-containing protein |
| HORVU5Hr1G055260 | 2,41 | -0,41 | Leucine-rich repeat-containing protein |
| HORVU7Hr1G055680 | 2,63 | -1,20 | Leucine-rich repeat-containing protein |
| HORVU7Hr1G000280 | 2,83 | -1,42 | Leucine-rich repeat-containing protein |
| HORVU2Hr1G003540 | 3,99 | -0,01 | Leucine-rich repeat-containing protein |
| HORVU1Hr1G054390 | 4,65 | 0,20 | lipoxygenase [EC:1.13.11.12] |
| HORVU7Hr1G106770 | 3,47 | -1,30 | LL-diaminopimelate aminotransferase [EC:2.6.1.83] |
| HORVU1Hr1G016200 | 3,75 | 0,57 | long-chain acyl-CoA synthetase [EC:6.2.1.3] |
| HORVU3Hr1G050430 | 6,30 | -1,36 | lycopene epsilon cyclase [EC:1.14.-.-] |
| HORVU3Hr1G095200 | 2,55 | 1,44 | MADS BOX protein |
| HORVU5Hr1G098890 | 2,30 | -0,03 | malate dehydrogenase [EC:1.1.1.37] |
| HORVU7Hr1G008690 | 2,05 | -0,82 | male germ cell-associated kinase [EC:2.7.11.22] |
| HORVU3Hr1G024890 | 4,68 | -0,64 | member of 'GDXG' family of lipolytic enzymes |
| HORVU5Hr1G093500 | 5,16 | 1,88 | member of 'GDXG' family of lipolytic enzymes |
| HORVU7Hr1G057260 | 6,15 | 0,41 | member of 'GDXG' family of lipolytic enzymes |
| HORVU2Hr1G007570 | 6,10 | 0,42 | methyltransferase |
| HORVU1Hr1G089680 | 3,99 | -0,14 | MFS transporter, SP family, solute carrier family 2 (facilitated glucose transporter), member 8 |
| HORVU2Hr1G085160 | 2,17 | 0,93 | MFS transporter, SP family, solute carrier family 2 (myo-inositol transporter), member 13 |
| HORVU6Hr1G009670 | 2,54 | -1,80 | minor histocompatibility antigen H13 [EC:3.4.23.-] |
| HORVU2Hr1G076510 | 2,99 | 0,02 | mitogen-activated kinase |
| HORVU1Hr1G078750 | 3,20 | -1,20 | mitogen-activated kinase |
| HORVU3Hr1G113170 | 2,99 | -1,55 | MKIAA1688 protein |
| HORVU7Hr1G091040 | 2,14 | 1,93 | mlo protein |
| HORVU5Hr1G089780 | 3,10 | 1,91 | molybdopterin cofactor sulfurase (MOSC) |
| HORVU7Hr1G084950 | 2,12 | 0,96 | monooxygenase |
| HORVU4Hr1G072320 | 3,98 | 0,12 | monooxygenase |
| HORVU4Hr1G072340 | 5,92 | -1,82 | monooxygenase |
| HORVU6Hr1G057770 | 2,69 | -0,58 | MSS1/TRME-related GTP-binding protein |
| HORVU7Hr1G071750 | 2,50 | -0,05 | multi-copper oxidase |
| HORVU5Hr1G047540 | 3,14 | 0,61 | multidrug resistance protein |
| HORVU3Hr1G063430 | 4,29 | -0,33 | multidrug resistance protein, MATE family |
| HORVU2Hr1G020140 | 2,75 | -1,04 | MYB family transcription factor |
| HORVU3Hr1G038700 | 4,45 | -1,46 | myb proto-oncogene protein, plant |
| HORVU1Hr1G050840 | 3,74 | 0,44 | myosin heavy chain-related |
| HORVU4Hr1G076160 | 4,28 | -0,19 | N-terminal acetyltransferase |
| HORVU3Hr1G057200 | 4,43 | 1,44 | NAD dependent epimerase/dehydratase |
| HORVU7Hr1G093370 | 4,64 | 0,54 | NAD dependent epimerase/dehydratase |
| HORVU3Hr1G059840 | 2,33 | 1,03 | naphthoate synthase [EC:4.1.3.36] |
| HORVU3Hr1G055870 | 3,14 | 0,19 | neutral ceramidase [EC:3.5.1.23] |
| HORVU3Hr1G066440 | 2,04 | 0,71 | NGEP-related |
| HORVU3Hr1G032350 | 7,22 | -1,45 | nitrate, formate, iron dehydrogenase |
| HORVU7Hr1G037070 | 2,91 | -0,32 | nucleolar protein7; estrogen receptor coactivator-related |
| HORVU4Hr1G044040 | 2,59 | -0,71 | nucleoside-diphosphate kinase [EC:2.7.4.6] |
| HORVU3Hr1G068300 | 2,67 | 0,60 | nucleotide-binding protein NBP35(yeast)-related |
| HORVU2Hr1G126100 | 3,26 | 1,39 | oligopeptide transporter-related |
| HORVU6Hr1G067740 | 5,74 | 1,25 | oligopeptide transporter-related |
| HORVU3Hr1G082460 | 6,50 | -0,31 | oligopeptide transporter-related |
| HORVU2Hr1G086990 | 2,76 | 1,53 | organic solute transporter-related |
| HORVU7Hr1G042800 | 2,63 | 0,30 | osmotic stress potassium transporter |
| HORVU7Hr1G092940 | 2,65 | -1,29 | osmotic stress potassium transporter |
| HORVU2Hr1G018190 | 3,43 | 1,40 | osmotic stress potassium transporter |
| HORVU6Hr1G079030 | 2,71 | -0,17 | outer membrane lipoprotein Blc |
| HORVU6Hr1G056570 | 6,99 | -0,53 | outer membrane lipoprotein Blc |
| HORVU7Hr1G028880 | 2,60 | -0,04 | oxidoreductase, 2OG-FE(II) oxigenase family protein |
| HORVU0Hr1G017000 | 8,99 | 0,42 | oxidoreductase, 2OG-FE(II) oxigenase family protein |
| HORVU1Hr1G085660 | 3,24 | 0,48 | oxidoreductase |
| HORVU7Hr1G052500 | 4,89 | 0,49 | oxysterol-binding protein-related |
| HORVU1Hr1G055600 | 2,88 | -0,75 | palmitoyl-protein thioesterase [EC:3.1.2.22] |
| HORVU1Hr1G055630 | 2,75 | -0,54 | palmitoyl-protein thioesterase/dolichyldiphosphatase 1 |
| HORVU7Hr1G086520 | 3,63 | -0,43 | peptidyl-prolyl cis-trans isomerase |
| HORVU4Hr1G012330 | 2,98 | 0,19 | periplasmic beta-glucosidase |
| HORVU5Hr1G097260 | 3,37 | 1,77 | peroxidase [EC:1.11.1.7] |
| HORVU5Hr1G059970 | 3,98 | 0,95 | PHD finger transcription factor |
| HORVU5Hr1G078310 | 2,16 | -0,96 | phosphatidylethanolamine binding protein |
| HORVU7Hr1G108230 | 2,13 | 1,94 | phosphoglycerate kinase |
| HORVU2Hr1G013730 | 4,82 | 1,63 | phospholipase C, delta [EC:3.1.4.11] |
| HORVU3Hr1G039540 | 2,12 | -0,55 | phospholipid-transporting ATPase [EC:3.6.3.1] |
| HORVU3Hr1G078620 | 2,58 | 0,90 | PIN; auxin efflux carrier family |
| HORVU2Hr1G103000 | 7,84 | 0,95 | polyphenol oxidase [EC:1.10.3.1] |
| HORVU4Hr1G003420 | 2,25 | -0,43 | probable membrane protein DUF221 |
| HORVU2Hr1G069440 | 2,21 | 1,12 | programmed cell death protein 2 (PCDC2) |
| HORVU6Hr1G008590 | 2,05 | 0,83 | programmed cell death protein 11 (pre-rRNA precessing protein RRP5) |
| HORVU1Hr1G053440 | 6,59 | 1,38 | proline dehydrogenase [EC:1.5.99.8] |
| HORVU6Hr1G093570 | 2,06 | -0,40 | prolyl 4-hydroxylase [EC:1.14.11.2] |
| HORVU5Hr1G108610 | 4,40 | -0,38 | prolyl 4-hydroxylase [EC:1.14.11.2] |
| HORVU4Hr1G063870 | 2,58 | 0,98 | proprotein convertase subtilisin/kexin |
| HORVU5Hr1G061990 | 3,35 | -0,47 | proprotein convertase subtilisin/kexin |
| HORVU3Hr1G077950 | 3,78 | -0,53 | proprotein convertase subtilisin/kexin |
| HORVU7Hr1G090870 | 4,00 | -1,95 | proprotein convertase subtilisin/kexin |
| HORVU6Hr1G010420 | 5,31 | -0,57 | proteasome inhibitor |
| HORVU2Hr1G033730 | 2,19 | 0,11 | proteasome maturation protein (UMP1) |
| HORVU3Hr1G018110 | 2,69 | -0,66 | protein phosphatase 2C |
| HORVU6Hr1G039940 | 6,37 | 0,83 | protein phosphatase 2C |
| HORVU7Hr1G029330 | 2,20 | 1,28 | protein phosphatase 2C homolog 2/3 [EC:3.1.3.16] |
| HORVU3Hr1G035720 | 2,31 | 0,48 | protein phosphatase |
| HORVU3Hr1G035750 | 2,40 | -0,27 | protein phosphatase |
| HORVU5Hr1G037760 | 3,73 | -0,84 | protein phosphatase |
| HORVU5Hr1G057330 | 3,40 | -1,95 | puromycin-sensitive aminopeptidase [EC:3.4.11.-] |
| HORVU6Hr1G070500 | 2,02 | 1,39 | putative glutamine amidotransferase |
| HORVU5Hr1G019030 | 9,51 | -0,40 | pyrophosphate--fructose-6-phosphate 1-phosphotransferase [EC:2.7.1.90] |
| HORVU4Hr1G056050 | 2,34 | -0,35 | pyruvate decarboxylase [EC:4.1.1.1] |
| HORVU2Hr1G119460 | 3,12 | -0,11 | pyruvate kinase |
| HORVU2Hr1G040570 | 3,06 | -0,34 | pyruvate kinase [EC:2.7.1.40] |
| HORVU2Hr1G045810 | 3,27 | -0,06 | Rab family, other |
| HORVU5Hr1G009840 | 2,01 | -1,06 | RAB GDP-dissociation inhibitor |
| HORVU3Hr1G020780 | 2,39 | 1,23 | raffinose synthase [EC:2.4.1.82] |
| HORVU7Hr1G048710 | 2,50 | 0,07 | raffinose synthase [EC:2.4.1.82] |
| HORVU6Hr1G029520 | 2,74 | -0,71 | RAG1-activating protein 1; MTN3-related |
| HORVU5Hr1G056470 | 2,36 | -0,53 | Ras-related protein Rab-11A |
| HORVU7Hr1G052840 | 3,71 | 1,86 | Ras-related protein Rab-11A |
| HORVU4Hr1G079250 | 2,04 | 0,33 | Ras-related protein Rab-18 |
| HORVU4Hr1G005870 | 2,18 | 0,51 | RDS/ ROM family |
| HORVU2Hr1G036240 | 3,61 | -0,60 | RDS/ ROM family |
| HORVU5Hr1G000260 | 6,19 | 0,48 | RDS/ ROM family |
| HORVU5Hr1G093270 | 3,21 | 0,06 | recombination protein RecA |
| HORVU3Hr1G079900 | 2,42 | 0,15 | multifunctional cyclin-dependent kinase-related |
| HORVU2Hr1G070700 | 3,81 | 1,47 | multifunctional cyclin-dependent kinase-related |
| HORVU6Hr1G050370 | 2,64 | -1,27 | replication factor A1 |
| HORVU3Hr1G030580 | 2,57 | -1,20 | replication protein A-related |
| HORVU3Hr1G010030 | 3,62 | 1,40 | repressor of RNA polymerase III transcription MAF1 |
| HORVU5Hr1G062490 | 2,38 | 0,24 | respiratory burst oxidase [EC:1.6.3.- 1.11.1.-] |
| HORVU5Hr1G024550 | 7,82 | 0,80 | respiratory burst oxidase [EC:1.6.3.- 1.11.1.-] |
| HORVU6Hr1G085590 | 2,60 | 1,72 | response regulator of two-component system |
| HORVU4Hr1G009210 | 2,29 | 0,25 | retinaldehyde binding protein-related |
| HORVU6Hr1G008930 | 2,21 | -1,14 | reverse transcriptases |
| HORVU7Hr1G115180 | 2,69 | -0,13 | ribonuclease P protein subunit P38-related |
| HORVU5Hr1G049160 | 2,33 | 0,91 | ribosomal protein S6 kinase; serine/threonine-protein kinase |
| HORVU4Hr1G050280 | 3,47 | 1,16 | RING FINGER and protease associated domain-containing |
| HORVU7Hr1G109180 | 4,19 | 1,75 | RING FINGER and protease associated domain-containing |
| HORVU2Hr1G017770 | 5,72 | -1,25 | RING FINGER and protease associated domain-containing |
| HORVU3Hr1G028280 | 2,08 | 0,80 | RING FINGER domain-containing |
| HORVU0Hr1G016480 | 3,26 | 1,45 | RING FINGER domain-containing |
| HORVU1Hr1G052700 | 4,77 | 0,59 | RING FINGER domain-containing |
| HORVU7Hr1G089530 | 4,92 | 1,21 | RING FINGER domain-containing |
| HORVU1Hr1G029510 | 5,71 | -0,32 | RING FINGER domain-containing |
| HORVU5Hr1G106470 | 2,08 | -1,11 | RING FINGER domain-containing, polycomb group component |
| HORVU0Hr1G003000 | 7,09 | -0,81 | RING FINGER domain-containing; sulfurotransferase (SULT) |
| HORVU1Hr1G037550 | 3,27 | -0,42 | RING FINGER protein 41, 151 |
| HORVU4Hr1G060130 | 2,57 | 1,32 | RING ZINC FINGER protein |
| HORVU7Hr1G053300 | 2,84 | 1,24 | RPM1-interacting protein 4 |
| HORVU2Hr1G000040 | 2,21 | -0,71 | SAP30 |
| HORVU5Hr1G052230 | 2,35 | -1,79 | SEC14 related protein |
| HORVU0Hr1G006630 | 5,06 | 1,24 | SEC15 |
| HORVU6Hr1G067670 | 3,23 | -0,03 | secretory carrier-associated membrane (SCAMP) |
| HORVU4Hr1G010160 | 2,61 | -0,96 | serine protease family S10 serine carboxypeptidase |
| HORVU3Hr1G096830 | 4,43 | 1,78 | serine protease family S10 serine carboxypeptidase |
| HORVU4Hr1G064280 | 3,42 | 1,75 | serine protease inhibitor, serpin |
| HORVU3Hr1G093140 | 3,28 | -0,58 | serine-threonine protein kinase |
| HORVU6Hr1G055820 | 3,41 | -1,27 | serine-threonine protein kinase |
| HORVU7Hr1G038650 | 3,54 | 1,00 | serine-threonine protein kinase |
| HORVU3Hr1G108000 | 5,05 | 1,28 | serine-threonine kinase |
| HORVU1Hr1G078410 | 2,78 | 0,60 | serine-threonine protein kinase |
| HORVU6Hr1G025940 | 2,92 | -0,82 | serine-threonine protein kinase |
| HORVU1Hr1G071060 | 4,09 | -0,30 | serine-threonine protein kinase |
| HORVU2Hr1G110230 | 4,59 | 1,74 | serine/threonine-protein kinase SRK2 [EC:2.7.11.1] |
| HORVU7Hr1G109250 | 2,69 | 0,95 | signal peptidase I [EC:3.4.21.89] |
| HORVU4Hr1G002330 | 2,80 | 0,04 | SLC24A6, NCKX6; solute carrier family 24 (sodium/potassium/calcium exchanger), member 6 |
| HORVU4Hr1G085820 | 2,51 | -0,23 | solute carrier family 35 member B |
| HORVU6Hr1G064740 | 2,40 | -0,43 | solute carrier family 36 (proton-coupled amino acid transporter), member 1 |
| HORVU3Hr1G059700 | 2,29 | 1,41 | STAM-binding protein [EC:3.1.2.15] |
| HORVU5Hr1G053070 | 2,80 | 0,38 | steroidogenic acute regulatory protein (STAR) |
| HORVU7Hr1G094690 | 4,37 | 0,39 | sterol regulatory element-binding protein |
| HORVU3Hr1G059230 | 2,27 | -1,31 | structural maintenance of chromosomes SMC family member |
| HORVU7Hr1G033230 | 8,19 | 1,10 | sucrose synthase [EC:2.4.1.13] |
| HORVU3Hr1G002280 | 2,97 | 1,22 | sucrose-phosphate synthase [EC:2.4.1.14] |
| HORVU1Hr1G038990 | 2,58 | 0,55 | sugar kinase |
| HORVU5Hr1G000010 | 2,06 | 0,33 | sugar transporter |
| HORVU5Hr1G066360 | 2,87 | -0,84 | sulfate transporter |
| HORVU7Hr1G019890 | 3,20 | 1,98 | sulfate transporter |
| HORVU7Hr1G101500 | 5,21 | -1,33 | sulfide:quinone oxidoreductase |
| HORVU7Hr1G043140 | 3,06 | -0,28 | symplekin-related |
| HORVU2Hr1G034110 | 5,86 | -1,84 | synaptosomal associated protein |
| HORVU2Hr1G045350 | 5,91 | -1,91 | syntaxin 1B/2/3 |
| HORVU3Hr1G091910 | 2,11 | -0,54 | TBC1 domain family member GTPase-activating protein |
| HORVU6Hr1G088820 | 2,12 | -1,76 | TBC1 domain family member GTPase-activating protein |
| HORVU7Hr1G105880 | 2,10 | 1,71 | tetraspanin |
| HORVU3Hr1G062600 | 2,12 | 0,78 | THAP4 protein |
| HORVU3Hr1G018650 | 2,32 | 1,41 | thiamine pyrophosphate enzyme |
| HORVU3Hr1G049070 | 2,32 | -1,83 | THIJ/PFPI |
| HORVU7Hr1G085170 | 7,53 | -1,45 | thioredoxin |
| HORVU1Hr1G057910 | 5,79 | -0,81 | threonyl-tRNA synthetase |
| HORVU1Hr1G005120 | 2,65 | -1,30 | TIM23 |
| HORVU3Hr1G023740 | 2,10 | 0,02 | TPR repeat containing protein |
| HORVU3Hr1G080830 | 3,50 | 1,72 | trans-cinnamate 4-monooxygenase [EC:1.14.13.11] |
| HORVU7Hr1G093630 | 6,51 | -0,80 | trans-cinnamate 4-monooxygenase [EC:1.14.13.11] |
| HORVU5Hr1G036330 | 2,03 | 0,57 | transcription factor GATA (GATA binding factor) |
| HORVU1Hr1G076480 | 2,09 | 0,76 | trehalose-6- phosphate synthase |
| HORVU7Hr1G047400 | 2,24 | 0,61 | tRNA-specific adenosine deaminase [EC:3.5.4.-]; cytosine deaminase |
| HORVU4Hr1G059170 | 3,15 | 1,83 | Trp repressor binding protein |
| HORVU4Hr1G076420 | 6,26 | 1,74 | Trp repressor binding protein |
| HORVU4Hr1G020110 | 2,48 | 1,47 | TUBBY-related |
| HORVU5Hr1G119650 | 2,07 | -0,51 | two-component sensor histidine kinase |
| HORVU6Hr1G080180 | 2,62 | -2,00 | two-component sensor histidine kinase |
| HORVU1Hr1G071260 | 4,53 | 1,64 | tyrosine aminotransferase [EC:2.6.1.5] |
| HORVU3Hr1G075040 | 4,94 | 1,34 | tyrosine decarboxylase [EC:4.1.1.25] |
| HORVU7Hr1G081930 | 2,56 | -0,03 | ubiquitin |
| HORVU2Hr1G104390 | 3,93 | -0,65 | ubiquitin |
| HORVU5Hr1G056730 | 4,45 | -1,09 | ubiquitin |
| HORVU5Hr1G101740 | 2,60 | 0,41 | ubiquitin thioesterase OTU1 [EC:3.1.2.-] |
| HORVU3Hr1G023230 | 2,35 | 1,30 | UDP-glucosyl transferase 73C [EC:2.4.1.-] |
| HORVU6Hr1G025460 | 4,84 | 1,29 | DUF292 |
| HORVU4Hr1G062110 | 5,93 | -0,50 | DUF292 |
| HORVU3Hr1G051080 | 2,02 | 1,22 | unknown |
| HORVU5Hr1G039210 | 2,02 | -0,01 | unknown |
| HORVU7Hr1G099520 | 2,02 | -0,84 | unknown |
| HORVU7Hr1G097000 | 2,02 | -1,34 | unknown |
| HORVU2Hr1G019250 | 2,03 | -1,34 | unknown |
| HORVU1Hr1G043900 | 2,03 | -2,00 | unknown |
| HORVU5Hr1G099550 | 2,03 | 1,47 | unknown |
| HORVU3Hr1G081100 | 2,04 | 0,84 | unknown |
| HORVU2Hr1G012310 | 2,04 | -0,86 | unknown |
| HORVU7Hr1G113290 | 2,05 | -0,13 | unknown |
| HORVU6Hr1G023770 | 2,05 | -1,40 | unknown |
| HORVU4Hr1G085310 | 2,05 | -0,99 | unknown |
| HORVU0Hr1G000990 | 2,06 | 0,22 | unknown |
| HORVU6Hr1G073010 | 2,06 | 0,16 | unknown |
| HORVU7Hr1G057760 | 2,07 | 1,54 | unknown |
| HORVU5Hr1G011090 | 2,08 | -1,08 | unknown |
| HORVU5Hr1G076340 | 2,09 | -1,40 | unknown |
| HORVU4Hr1G009270 | 2,10 | -0,68 | unknown |
| HORVU1Hr1G070580 | 2,10 | 0,98 | unknown |
| HORVU6Hr1G023380 | 2,11 | 0,52 | unknown |
| HORVU2Hr1G047050 | 2,13 | -0,67 | unknown |
| HORVU1Hr1G053890 | 2,14 | -1,57 | unknown |
| HORVU3Hr1G071220 | 2,14 | -0,02 | unknown |
| HORVU2Hr1G043780 | 2,16 | 1,58 | unknown |
| HORVU5Hr1G021690 | 2,16 | -0,48 | unknown |
| HORVU4Hr1G072630 | 2,16 | 0,60 | unknown |
| HORVU3Hr1G113000 | 2,16 | -0,33 | unknown |
| HORVU7Hr1G089660 | 2,17 | 0,91 | unknown |
| HORVU7Hr1G119620 | 2,17 | -0,29 | unknown |
| HORVU2Hr1G023680 | 2,18 | -1,00 | unknown |
| HORVU3Hr1G062690 | 2,18 | 1,20 | unknown |
| HORVU6Hr1G012270 | 2,19 | -1,04 | unknown |
| HORVU3Hr1G083330 | 2,19 | -1,04 | unknown |
| HORVU5Hr1G096190 | 2,20 | 0,40 | unknown |
| HORVU3Hr1G080370 | 2,20 | -1,75 | unknown |
| HORVU5Hr1G006880 | 2,20 | -0,78 | unknown |
| HORVU5Hr1G023350 | 2,21 | 0,31 | unknown |
| HORVU2Hr1G099270 | 2,22 | -0,65 | unknown |
| HORVU1Hr1G035290 | 2,23 | -1,18 | unknown |
| HORVU5Hr1G093350 | 2,23 | -0,09 | unknown |
| HORVU2Hr1G100600 | 2,23 | -0,75 | unknown |
| HORVU7Hr1G041430 | 2,24 | -1,73 | unknown |
| HORVU6Hr1G038700 | 2,24 | 0,10 | unknown |
| HORVU4Hr1G000590 | 2,24 | -1,39 | unknown |
| HORVU2Hr1G082810 | 2,25 | 1,64 | unknown |
| HORVU7Hr1G117970 | 2,25 | 0,05 | unknown |
| HORVU5Hr1G068200 | 2,25 | -0,06 | unknown |
| HORVU6Hr1G076520 | 2,26 | -0,90 | unknown |
| HORVU0Hr1G010640 | 2,26 | 1,86 | unknown |
| HORVU4Hr1G053560 | 2,28 | 0,32 | unknown |
| HORVU3Hr1G017120 | 2,28 | 0,45 | unknown |
| HORVU3Hr1G013840 | 2,28 | -1,99 | unknown |
| HORVU1Hr1G088280 | 2,29 | 0,80 | unknown |
| HORVU4Hr1G020030 | 2,30 | 0,01 | unknown |
| HORVU7Hr1G080670 | 2,30 | 1,71 | unknown |
| HORVU5Hr1G119270 | 2,31 | -1,15 | unknown |
| HORVU7Hr1G093830 | 2,31 | -1,71 | unknown |
| HORVU7Hr1G029900 | 2,32 | -1,69 | unknown |
| HORVU6Hr1G019450 | 2,32 | -0,72 | unknown |
| HORVU2Hr1G090280 | 2,33 | -1,12 | unknown |
| HORVU6Hr1G013580 | 2,34 | -0,23 | unknown |
| HORVU3Hr1G078520 | 2,34 | -1,64 | unknown |
| HORVU4Hr1G042640 | 2,34 | -1,34 | unknown |
| HORVU2Hr1G093180 | 2,35 | -1,15 | unknown |
| HORVU7Hr1G006590 | 2,37 | -1,12 | unknown |
| HORVU7Hr1G052530 | 2,37 | -1,04 | unknown |
| HORVU6Hr1G034990 | 2,38 | -0,80 | unknown |
| HORVU6Hr1G016750 | 2,38 | -0,33 | unknown |
| HORVU1Hr1G064850 | 2,40 | -1,50 | unknown |
| HORVU2Hr1G098080 | 2,40 | 0,06 | unknown |
| HORVU3Hr1G117890 | 2,41 | 1,42 | unknown |
| HORVU0Hr1G039620 | 2,41 | -1,81 | unknown |
| HORVU4Hr1G065800 | 2,42 | -1,58 | unknown |
| HORVU5Hr1G095000 | 2,42 | -1,28 | unknown |
| HORVU6Hr1G088710 | 2,42 | -1,11 | unknown |
| HORVU4Hr1G009380 | 2,43 | 0,56 | unknown |
| HORVU3Hr1G066650 | 2,44 | -1,36 | unknown |
| HORVU7Hr1G030370 | 2,44 | 0,75 | unknown |
| HORVU0Hr1G008850 | 2,44 | 1,38 | unknown |
| HORVU1Hr1G094050 | 2,44 | 1,19 | unknown |
| HORVU4Hr1G056950 | 2,44 | -1,24 | unknown |
| HORVU5Hr1G058170 | 2,45 | 1,90 | unknown |
| HORVU3Hr1G084970 | 2,45 | 1,04 | unknown |
| HORVU2Hr1G046590 | 2,45 | -0,34 | unknown |
| HORVU5Hr1G085220 | 2,46 | 0,72 | unknown |
| HORVU2Hr1G068200 | 2,46 | 0,25 | unknown |
| HORVU6Hr1G066810 | 2,46 | 0,44 | unknown |
| HORVU1Hr1G038950 | 2,46 | -0,96 | unknown |
| HORVU4Hr1G057290 | 2,48 | -0,22 | unknown |
| HORVU3Hr1G060570 | 2,48 | -1,06 | unknown |
| HORVU3Hr1G056650 | 2,48 | 0,16 | unknown |
| HORVU7Hr1G114030 | 2,48 | -0,36 | unknown |
| HORVU5Hr1G023990 | 2,50 | 1,23 | unknown |
| HORVU4Hr1G011250 | 2,50 | 0,60 | unknown |
| HORVU3Hr1G108630 | 2,51 | 0,58 | unknown |
| HORVU2Hr1G094960 | 2,52 | 0,48 | unknown |
| HORVU0Hr1G017690 | 2,52 | -1,01 | unknown |
| HORVU5Hr1G112930 | 2,53 | 1,50 | unknown |
| HORVU0Hr1G003010 | 2,54 | -0,56 | unknown |
| HORVU5Hr1G077450 | 2,54 | -0,69 | unknown |
| HORVU7Hr1G052350 | 2,55 | -0,06 | unknown |
| HORVU6Hr1G009480 | 2,55 | -0,31 | unknown |
| HORVU3Hr1G099220 | 2,56 | 1,46 | unknown |
| HORVU2Hr1G084260 | 2,56 | -0,30 | unknown |
| HORVU2Hr1G060640 | 2,56 | -1,12 | unknown |
| HORVU3Hr1G064130 | 2,59 | 0,48 | unknown |
| HORVU5Hr1G092660 | 2,59 | -1,55 | unknown |
| HORVU5Hr1G056490 | 2,59 | -0,96 | unknown |
| HORVU3Hr1G034670 | 2,60 | -0,03 | unknown |
| HORVU3Hr1G059720 | 2,61 | 0,21 | unknown |
| HORVU7Hr1G050160 | 2,62 | -1,70 | unknown |
| HORVU5Hr1G032370 | 2,62 | -0,86 | unknown |
| HORVU1Hr1G010940 | 2,66 | 1,69 | unknown |
| HORVU7Hr1G093080 | 2,67 | 1,24 | unknown |
| HORVU7Hr1G080780 | 2,68 | 1,36 | unknown |
| HORVU7Hr1G096140 | 2,68 | 1,62 | unknown |
| HORVU5Hr1G112900 | 2,70 | -0,40 | unknown |
| HORVU7Hr1G040430 | 2,70 | 1,35 | unknown |
| HORVU2Hr1G094630 | 2,71 | 1,90 | unknown |
| HORVU3Hr1G006740 | 2,72 | -1,10 | unknown |
| HORVU4Hr1G053570 | 2,72 | -0,09 | unknown |
| HORVU2Hr1G088540 | 2,73 | -0,45 | unknown |
| HORVU4Hr1G061850 | 2,73 | 0,01 | unknown |
| HORVU3Hr1G107320 | 2,74 | 1,38 | unknown |
| HORVU5Hr1G070810 | 2,77 | 1,09 | unknown |
| HORVU3Hr1G019070 | 2,77 | 0,92 | unknown |
| HORVU7Hr1G088730 | 2,77 | -0,84 | unknown |
| HORVU4Hr1G051010 | 2,80 | 0,55 | unknown |
| HORVU5Hr1G061660 | 2,81 | -0,85 | unknown |
| HORVU5Hr1G099070 | 2,82 | 0,62 | unknown |
| HORVU2Hr1G100720 | 2,82 | -0,85 | unknown |
| HORVU4Hr1G087500 | 2,82 | 0,54 | unknown |
| HORVU3Hr1G073960 | 2,83 | 0,37 | unknown |
| HORVU5Hr1G079460 | 2,83 | 0,46 | unknown |
| HORVU3Hr1G057700 | 2,83 | -0,67 | unknown |
| HORVU2Hr1G125360 | 2,84 | 0,84 | unknown |
| HORVU2Hr1G004660 | 2,85 | -1,31 | unknown |
| HORVU7Hr1G116310 | 2,85 | 1,41 | unknown |
| HORVU1Hr1G050780 | 2,86 | 0,41 | unknown |
| HORVU5Hr1G122510 | 2,86 | -1,63 | unknown |
| HORVU0Hr1G021630 | 2,86 | -0,75 | unknown |
| HORVU7Hr1G002110 | 2,87 | 1,03 | unknown |
| HORVU5Hr1G050870 | 2,87 | 0,46 | unknown |
| HORVU2Hr1G028330 | 2,88 | 0,98 | unknown |
| HORVU4Hr1G074780 | 2,89 | 0,17 | unknown |
| HORVU2Hr1G019400 | 2,90 | 1,30 | unknown |
| HORVU5Hr1G051140 | 2,92 | -1,14 | unknown |
| HORVU5Hr1G092310 | 2,93 | -1,06 | unknown |
| HORVU3Hr1G049520 | 2,95 | -1,58 | unknown |
| HORVU5Hr1G023960 | 2,95 | -1,59 | unknown |
| HORVU5Hr1G099180 | 2,96 | -0,63 | unknown |
| HORVU6Hr1G088610 | 3,01 | 0,72 | unknown |
| HORVU2Hr1G045170 | 3,02 | 1,22 | unknown |
| HORVU7Hr1G114510 | 3,04 | 0,51 | unknown |
| HORVU4Hr1G003440 | 3,05 | -1,25 | unknown |
| HORVU5Hr1G076100 | 3,06 | -0,66 | unknown |
| HORVU2Hr1G075220 | 3,09 | 0,79 | unknown |
| HORVU4Hr1G076820 | 3,10 | 0,22 | unknown |
| HORVU2Hr1G038860 | 3,10 | -1,30 | unknown |
| HORVU3Hr1G045460 | 3,10 | -0,55 | unknown |
| HORVU6Hr1G020330 | 3,10 | 1,54 | unknown |
| HORVU4Hr1G069280 | 3,11 | -0,97 | unknown |
| HORVU5Hr1G048030 | 3,15 | 1,54 | unknown |
| HORVU6Hr1G035610 | 3,15 | 1,39 | unknown |
| HORVU1Hr1G028900 | 3,17 | 0,21 | unknown |
| HORVU2Hr1G032680 | 3,18 | -0,81 | unknown |
| HORVU6Hr1G028660 | 3,19 | -0,02 | unknown |
| HORVU4Hr1G020290 | 3,20 | -1,51 | unknown |
| HORVU4Hr1G057520 | 3,20 | -0,31 | unknown |
| HORVU3Hr1G003980 | 3,20 | 1,51 | unknown |
| HORVU5Hr1G097500 | 3,21 | 1,12 | unknown |
| HORVU2Hr1G028190 | 3,22 | 0,21 | unknown |
| HORVU5Hr1G058020 | 3,22 | 0,16 | unknown |
| HORVU1Hr1G036340 | 3,24 | -0,59 | unknown |
| HORVU7Hr1G077970 | 3,24 | -0,23 | unknown |
| HORVU2Hr1G088760 | 3,26 | -0,66 | unknown |
| HORVU3Hr1G079540 | 3,29 | 1,18 | unknown |
| HORVU6Hr1G082310 | 3,29 | -1,23 | unknown |
| HORVU1Hr1G045220 | 3,31 | 0,99 | unknown |
| HORVU3Hr1G060730 | 3,32 | 1,32 | unknown |
| HORVU7Hr1G098440 | 3,32 | 1,03 | unknown |
| HORVU7Hr1G113530 | 3,33 | 1,22 | unknown |
| HORVU6Hr1G019510 | 3,34 | 0,26 | unknown |
| HORVU2Hr1G033060 | 3,38 | -0,91 | unknown |
| HORVU1Hr1G071750 | 3,39 | -0,93 | unknown |
| HORVU3Hr1G031140 | 3,40 | -0,76 | unknown |
| HORVU5Hr1G058710 | 3,40 | -1,93 | unknown |
| HORVU4Hr1G023850 | 3,40 | 0,81 | unknown |
| HORVU6Hr1G032890 | 3,41 | -1,49 | unknown |
| HORVU1Hr1G042030 | 3,45 | 1,91 | unknown |
| HORVU0Hr1G039260 | 3,47 | 0,81 | unknown |
| HORVU2Hr1G058850 | 3,48 | 0,92 | unknown |
| HORVU7Hr1G071530 | 3,49 | 0,65 | unknown |
| HORVU2Hr1G063820 | 3,50 | 1,82 | unknown |
| HORVU2Hr1G015940 | 3,58 | 0,16 | unknown |
| HORVU3Hr1G086690 | 3,60 | 1,46 | unknown |
| HORVU2Hr1G105650 | 3,61 | 0,75 | unknown |
| HORVU2Hr1G102880 | 3,61 | -0,05 | unknown |
| HORVU4Hr1G053150 | 3,62 | 1,74 | unknown |
| HORVU1Hr1G013270 | 3,62 | 0,67 | unknown |
| HORVU5Hr1G057740 | 3,64 | 1,07 | unknown |
| HORVU5Hr1G106720 | 3,66 | -1,07 | unknown |
| HORVU5Hr1G040160 | 3,67 | 1,96 | unknown |
| HORVU3Hr1G096850 | 3,71 | 0,91 | unknown |
| HORVU1Hr1G085640 | 3,72 | -0,95 | unknown |
| HORVU1Hr1G052290 | 3,72 | 1,85 | unknown |
| HORVU5Hr1G079570 | 3,75 | -0,58 | unknown |
| HORVU6Hr1G069940 | 3,75 | -0,52 | unknown |
| HORVU3Hr1G023780 | 3,77 | 1,62 | unknown |
| HORVU6Hr1G027970 | 3,77 | 0,04 | unknown |
| HORVU5Hr1G046850 | 3,79 | 0,77 | unknown |
| HORVU7Hr1G085980 | 3,79 | -1,34 | unknown |
| HORVU5Hr1G093120 | 3,83 | 0,26 | unknown |
| HORVU7Hr1G099220 | 3,88 | 1,03 | unknown |
| HORVU6Hr1G074690 | 3,89 | 1,97 | unknown |
| HORVU4Hr1G009300 | 3,92 | -0,33 | unknown |
| HORVU7Hr1G024980 | 3,96 | 0,02 | unknown |
| HORVU2Hr1G118700 | 3,98 | -0,21 | unknown |
| HORVU5Hr1G036790 | 3,98 | -1,04 | unknown |
| HORVU7Hr1G114950 | 4,01 | 0,11 | unknown |
| HORVU5Hr1G060980 | 4,02 | 1,72 | unknown |
| HORVU0Hr1G039930 | 4,03 | -0,59 | unknown |
| HORVU2Hr1G066080 | 4,03 | 1,06 | unknown |
| HORVU4Hr1G090660 | 4,03 | 0,12 | unknown |
| HORVU1Hr1G065600 | 4,04 | 0,56 | unknown |
| HORVU4Hr1G083070 | 4,04 | 1,16 | unknown |
| HORVU5Hr1G020320 | 4,08 | -1,89 | unknown |
| HORVU3Hr1G002980 | 4,09 | 0,41 | unknown |
| HORVU3Hr1G071490 | 4,11 | 1,55 | unknown |
| HORVU3Hr1G087480 | 4,11 | -0,18 | unknown |
| HORVU4Hr1G024380 | 4,12 | 0,84 | unknown |
| HORVU3Hr1G112810 | 4,13 | -1,25 | unknown |
| HORVU3Hr1G007210 | 4,15 | -0,36 | unknown |
| HORVU5Hr1G098190 | 4,16 | 1,84 | unknown |
| HORVU7Hr1G113300 | 4,17 | -0,02 | unknown |
| HORVU3Hr1G069660 | 4,20 | 1,40 | unknown |
| HORVU5Hr1G103850 | 4,21 | 0,79 | unknown |
| HORVU6Hr1G063250 | 4,23 | 0,50 | unknown |
| HORVU3Hr1G087740 | 4,24 | 0,72 | unknown |
| HORVU2Hr1G020420 | 4,26 | -1,06 | unknown |
| HORVU1Hr1G089830 | 4,26 | 1,93 | unknown |
| HORVU4Hr1G027260 | 4,29 | 1,52 | unknown |
| HORVU2Hr1G027810 | 4,29 | 0,01 | unknown |
| HORVU7Hr1G091520 | 4,33 | 0,46 | unknown |
| HORVU5Hr1G056100 | 4,37 | -0,62 | unknown |
| HORVU6Hr1G062220 | 4,38 | 1,94 | unknown |
| HORVU4Hr1G014530 | 4,39 | 0,43 | unknown |
| HORVU2Hr1G105740 | 4,41 | 1,65 | unknown |
| HORVU3Hr1G026920 | 4,42 | 1,25 | unknown |
| HORVU7Hr1G089240 | 4,46 | 0,14 | unknown |
| HORVU6Hr1G070290 | 4,46 | 1,47 | unknown |
| HORVU2Hr1G061850 | 4,49 | -0,10 | unknown |
| HORVU1Hr1G043920 | 4,49 | 1,31 | unknown |
| HORVU6Hr1G033070 | 4,51 | -1,38 | unknown |
| HORVU4Hr1G078350 | 4,54 | 1,46 | unknown |
| HORVU6Hr1G034570 | 4,58 | 0,78 | unknown |
| HORVU3Hr1G021700 | 4,58 | -1,61 | unknown |
| HORVU1Hr1G026650 | 4,59 | 0,32 | unknown |
| HORVU1Hr1G043080 | 4,59 | -1,63 | unknown |
| HORVU4Hr1G002770 | 4,65 | -1,34 | unknown |
| HORVU4Hr1G070130 | 4,66 | -0,92 | unknown |
| HORVU5Hr1G100790 | 4,67 | -0,21 | unknown |
| HORVU4Hr1G054870 | 4,73 | -1,74 | unknown |
| HORVU5Hr1G115100 | 4,78 | -1,53 | unknown |
| HORVU3Hr1G047220 | 4,84 | -0,70 | unknown |
| HORVU3Hr1G038480 | 4,84 | -0,28 | unknown |
| HORVU2Hr1G102620 | 4,87 | 1,63 | unknown |
| HORVU4Hr1G062970 | 4,88 | -0,98 | unknown |
| HORVU7Hr1G012610 | 4,88 | 1,48 | unknown |
| HORVU5Hr1G080790 | 4,88 | -0,55 | unknown |
| HORVU2Hr1G018670 | 4,91 | 1,50 | unknown |
| HORVU4Hr1G061340 | 4,91 | 0,29 | unknown |
| HORVU3Hr1G026710 | 4,93 | -0,44 | unknown |
| HORVU4Hr1G016840 | 4,95 | -0,89 | unknown |
| HORVU2Hr1G111600 | 5,02 | 1,69 | unknown |
| HORVU5Hr1G107290 | 5,04 | 1,25 | unknown |
| HORVU4Hr1G008590 | 5,05 | -1,47 | unknown |
| HORVU4Hr1G071360 | 5,09 | 1,18 | unknown |
| HORVU2Hr1G012550 | 5,12 | 1,97 | unknown |
| HORVU2Hr1G124740 | 5,15 | 0,36 | unknown |
| HORVU3Hr1G062700 | 5,15 | -0,17 | unknown |
| HORVU5Hr1G063950 | 5,16 | -1,43 | unknown |
| HORVU6Hr1G064620 | 5,19 | 1,60 | unknown |
| HORVU4Hr1G081570 | 5,21 | -0,10 | unknown |
| HORVU6Hr1G029540 | 5,22 | 0,17 | unknown |
| HORVU7Hr1G040860 | 5,23 | -0,61 | unknown |
| HORVU4Hr1G078310 | 5,25 | 1,06 | unknown |
| HORVU2Hr1G106780 | 5,30 | -1,15 | unknown |
| HORVU7Hr1G040030 | 5,31 | 1,53 | unknown |
| HORVU7Hr1G042740 | 5,31 | 0,86 | unknown |
| HORVU4Hr1G017240 | 5,34 | 0,51 | unknown |
| HORVU6Hr1G073540 | 5,40 | 0,24 | unknown |
| HORVU5Hr1G096950 | 5,40 | 0,60 | unknown |
| HORVU2Hr1G103780 | 5,40 | -1,57 | unknown |
| HORVU2Hr1G033070 | 5,43 | -0,46 | unknown |
| HORVU3Hr1G056640 | 5,45 | 0,84 | unknown |
| HORVU4Hr1G002780 | 5,45 | -1,78 | unknown |
| HORVU6Hr1G011860 | 5,46 | -0,96 | unknown |
| HORVU2Hr1G079510 | 5,52 | 0,88 | unknown |
| HORVU1Hr1G025440 | 5,53 | -1,40 | unknown |
| HORVU5Hr1G012120 | 5,55 | 1,96 | unknown |
| HORVU4Hr1G066140 | 5,56 | 1,73 | unknown |
| HORVU2Hr1G078380 | 5,61 | -0,26 | unknown |
| HORVU3Hr1G049730 | 5,64 | 0,61 | unknown |
| HORVU6Hr1G088960 | 5,64 | -1,38 | unknown |
| HORVU7Hr1G104830 | 5,67 | 0,82 | unknown |
| HORVU4Hr1G017260 | 5,71 | -0,23 | unknown |
| HORVU2Hr1G098160 | 5,76 | 1,13 | unknown |
| HORVU1Hr1G051270 | 5,76 | -1,07 | unknown |
| HORVU4Hr1G055270 | 5,77 | 1,78 | unknown |
| HORVU7Hr1G121420 | 5,86 | -0,14 | unknown |
| HORVU0Hr1G021850 | 5,94 | 0,50 | unknown |
| HORVU7Hr1G089850 | 6,08 | -0,43 | unknown |
| HORVU2Hr1G091910 | 6,16 | 0,16 | unknown |
| HORVU7Hr1G086250 | 6,18 | 1,54 | unknown |
| HORVU0Hr1G022020 | 6,22 | 1,58 | unknown |
| HORVU7Hr1G075720 | 6,24 | -0,70 | unknown |
| HORVU3Hr1G069320 | 6,29 | 1,08 | unknown |
| HORVU2Hr1G067700 | 6,30 | -1,76 | unknown |
| HORVU3Hr1G070700 | 6,30 | 1,44 | unknown |
| HORVU4Hr1G048700 | 6,33 | 0,79 | unknown |
| HORVU5Hr1G044370 | 6,34 | -1,46 | unknown |
| HORVU1Hr1G042810 | 6,37 | -0,09 | unknown |
| HORVU2Hr1G117400 | 6,48 | 1,75 | unknown |
| HORVU4Hr1G000850 | 6,52 | 1,79 | unknown |
| HORVU4Hr1G054980 | 6,53 | -0,28 | unknown |
| HORVU1Hr1G019770 | 6,61 | 1,58 | unknown |
| HORVU5Hr1G011440 | 6,68 | 1,56 | unknown |
| HORVU2Hr1G041180 | 6,90 | 0,83 | unknown |
| HORVU4Hr1G077280 | 6,93 | 1,96 | unknown |
| HORVU3Hr1G014140 | 6,94 | 1,07 | unknown |
| HORVU3Hr1G050080 | 6,96 | 1,30 | unknown |
| HORVU5Hr1G068060 | 7,04 | -1,00 | unknown |
| HORVU2Hr1G108730 | 7,08 | 1,56 | unknown |
| HORVU4Hr1G073230 | 7,08 | 0,17 | unknown |
| HORVU1Hr1G085720 | 7,08 | -0,40 | unknown |
| HORVU2Hr1G027480 | 7,15 | 1,19 | unknown |
| HORVU7Hr1G001040 | 7,25 | -1,17 | unknown |
| HORVU1Hr1G091010 | 7,26 | -0,51 | unknown |
| HORVU6Hr1G034490 | 7,38 | 0,91 | unknown |
| HORVU3Hr1G021190 | 7,42 | -0,36 | unknown |
| HORVU1Hr1G081240 | 7,52 | 0,05 | unknown |
| HORVU5Hr1G048810 | 7,57 | -1,10 | unknown |
| HORVU4Hr1G050510 | 7,63 | 0,99 | unknown |
| HORVU5Hr1G067360 | 7,78 | -1,79 | unknown |
| HORVU0Hr1G023150 | 7,88 | -0,82 | unknown |
| HORVU1Hr1G005230 | 8,11 | -1,24 | unknown |
| HORVU6Hr1G011850 | 8,23 | -1,99 | unknown |
| HORVU5Hr1G010880 | 8,26 | 0,22 | unknown |
| HORVU2Hr1G033090 | 8,34 | -0,40 | unknown |
| HORVU7Hr1G101580 | 8,40 | 0,60 | unknown |
| HORVU2Hr1G116800 | 8,62 | 0,94 | unknown |
| HORVU3Hr1G056110 | 8,66 | 0,06 | unknown |
| HORVU3Hr1G026540 | 8,75 | 0,45 | unknown |
| HORVU2Hr1G101140 | 8,97 | -1,45 | unknown |
| HORVU1Hr1G058940 | 9,10 | 1,55 | unknown |
| HORVU5Hr1G013600 | 9,14 | -0,30 | unknown |
| HORVU4Hr1G054200 | 9,18 | -0,60 | unknown |
| HORVU3Hr1G001920 | 9,18 | 0,74 | unknown |
| HORVU3Hr1G002000 | 11,95 | -1,26 | unknown |
| HORVU1Hr1G011430 | 2,57 | -0,12 | uridine nucleosidase [EC:3.2.2.3] |
| HORVU2Hr1G109990 | 3,57 | -0,28 | V-type H+-transporting ATPase subunit I [EC:3.6.3.14] |
| HORVU1Hr1G046630 | 4,11 | -1,58 | valyl-tRNA synthetase [EC:6.1.1.9] |
| HORVU7Hr1G096840 | 2,07 | -0,44 | vernalization-insensitive protein 3 |
| HORVU6Hr1G060760 | 2,79 | 0,94 | vesicle-associated membrane protein-associated protein |
| HORVU3Hr1G029210 | 3,66 | 1,83 | WASP-1 |
| HORVU1Hr1G060850 | 2,07 | 1,23 | WD repeat-containing protein 23 |
| HORVU3Hr1G083500 | 4,16 | 0,83 | WDSAM1 protein |
| HORVU7Hr1G040790 | 5,24 | 1,20 | WDSAM1 protein |
| HORVU6Hr1G089130 | 2,13 | -0,59 | xenotropic and polytropic retrovirus receptor 1-related |
| HORVU2Hr1G031400 | 2,51 | -0,97 | xenotropic and polytropic retrovirus receptor 1-related |
| HORVU6Hr1G078650 | 2,92 | 0,07 | xyloglucan fucosyltransferase [EC:2.4.1.-] |
| HORVU6Hr1G067470 | 6,00 | 1,11 | xyloglucan:xyloglucosyl transferase [EC:2.4.1.207] |
| HORVU1Hr1G023520 | 2,05 | 0,62 | zinc finger five domain containing protein |
| HORVU3Hr1G006100 | 3,01 | 1,94 | zinc finger five domain containing protein |
| HORVU3Hr1G055350 | 4,72 | -0,03 | zinc finger five domain containing protein |
| HORVU1Hr1G029460 | 5,04 | 1,54 | zinc finger five domain containing protein |
| HORVU3Hr1G028020 | 7,42 | 1,74 | zinc finger five domain containing protein |
| HORVU2Hr1G023770 | 9,33 | 0,53 | zinc finger five domain containing protein |
| HORVU6Hr1G046420 | 2,41 | 0,50 | zinc finger five domain containing protein |
| HORVU1Hr1G028920 | 2,87 | -0,44 | zinc/iron transporter |
