# Supplementary material for: Differential Expression Profiling of Microspores During the Early Stages of Isolated Microspore Culture Using the Responsive Barley Cultivar Gobernadora
Source: G3 (Bethesda). 2018 Mar 12;8(5):1603–14. doi: 10.1534/g3.118.200208 (PMC5940152; doi:10.1534/g3.118.200208)
Supplement: Supplementary file 6 [file 1603TableS6.docx]

Supplementary Table 6: Gene functional annotation for genes in cluster 6

| Gene stable ID (cluster 6) | logFC D2-D0 | logFC D5-D2 | Gene function annotation |
| --- | --- | --- | --- |
| HORVU7Hr1G082830 | 0,49 | 2,90 | 1,4-alpha-glucan branching enzyme [EC:2.4.1.18] |
| HORVU7Hr1G095960 | -1,30 | 2,17 | 12-oxophytodienoic acid reductase [EC:1.3.1.42] |
| HORVU7Hr1G046970 | 0,03 | 2,84 | 2-acylglycerol O-acyltransferase 2 [EC:2.3.1.22] |
| HORVU6Hr1G006560 | 1,90 | 2,96 | 2-oxoisovalerate dehydrogenase E1 component, beta subunit [EC:1.2.4.4] |
| HORVU2Hr1G012440 | 0,84 | 3,54 | 2-oxoisovalerate dehydrogenase E2 component (dihydrolipoyl transacylase) [EC:2.3.1.168] |
| HORVU5Hr1G012900 | 0,38 | 3,14 | 3-methylcrotonyl-CoA carboxylase alpha subunit [EC:6.4.1.4] |
| HORVU5Hr1G094300 | -0,05 | 3,57 | 3'-phosphoadenosine 5'-phosphosulfate synthase [EC:2.7.7.4 2.7.1.25] |
| HORVU6Hr1G030390 | 0,29 | 3,70 | 4-coumarate--CoA ligase [EC:6.2.1.12] |
| HORVU7Hr1G111130 | 1,60 | 4,99 | 4-coumarate--CoA ligase [EC:6.2.1.12] |
| HORVU2Hr1G122370 | -0,15 | 2,03 | 5-methylthioribose kinase [EC:2.7.1.100] |
| HORVU1Hr1G017380 | 0,72 | 2,96 | 5'-AMP-activated protein kinase, catalytic alpha subunit [EC:2.7.11.11] |
| HORVU2Hr1G027080 | 1,19 | 3,30 | 5'-AMP-activated protein kinase, catalytic alpha subunit [EC:2.7.11.11] |
| HORVU1Hr1G050110 | 1,22 | 3,77 | abscisic acid receptor PYR/PYL family |
| HORVU3Hr1G105880 | -0,81 | 2,90 | acetyl-CoA carboxylase / biotin carboxylase [EC:6.4.1.2 6.3.4.14] |
| HORVU5Hr1G042180 | -0,86 | 2,49 | acid phosphatase related |
| HORVU1Hr1G047440 | -1,31 | 2,58 | actin, other eukaryote |
| HORVU1Hr1G074350 | -0,18 | 2,31 | actin, other eukaryote |
| HORVU5Hr1G025960 | 1,79 | 4,30 | acyl carrier protein / zinc finger protein 593-related |
| HORVU7Hr1G083490 | -0,11 | 2,81 | acyl-CoA oxidase [EC:1.3.3.6] |
| HORVU7Hr1G029110 | 0,01 | 2,17 | acyl-CoA oxidase [EC:1.3.3.6] |
| HORVU0Hr1G003890 | -1,41 | 2,96 | adenylyl-sulfate reductase (glutathione) [EC:1.8.4.9] |
| HORVU7Hr1G112900 | -0,58 | 4,67 | adiponectin receptor |
| HORVU7Hr1G115600 | 1,83 | 7,04 | adiponectin receptor |
| HORVU4Hr1G075360 | -0,68 | 2,05 | alanine-glyoxylate transaminase / (R)-3-amino-2-methylpropionate-pyruvate transaminase [EC:2.6.1.44 2.6.1.40] |
| HORVU7Hr1G042880 | -1,34 | 4,76 | aldehyde dehydrogenase (NAD+) [EC:1.2.1.3] |
| HORVU6Hr1G001270 | -0,32 | 8,07 | aldehyde dehydrogenase-related |
| HORVU6Hr1G081000 | 0,39 | 4,54 | allene oxide cyclase [EC:5.3.99.6] |
| HORVU7Hr1G039260 | 0,35 | 2,24 | alpha-1,4-galacturonosyltransferase [EC:2.4.1.43] |
| HORVU7Hr1G082850 | 0,73 | 2,37 | alpha-amylase; GAG-POL-related retrotransposon |
| HORVU3Hr1G038060 | -0,20 | 3,69 | alpha-glucosidase |
| HORVU2Hr1G032780 | -1,83 | 3,03 | alpha/beta hydrolase fold-containing protein |
| HORVU3Hr1G103960 | -0,28 | 9,70 | alpha/beta hydrolase fold-containing protein |
| HORVU3Hr1G058320 | -0,22 | 2,49 | alpha/beta hydrolase fold-containing protein |
| HORVU6Hr1G033060 | -0,02 | 5,35 | alpha/beta hydrolase fold-containing protein |
| HORVU0Hr1G040440 | 0,29 | 2,39 | alpha/beta hydrolase fold-containing protein |
| HORVU4Hr1G086270 | 0,43 | 3,26 | alpha/beta hydrolase fold-containing protein |
| HORVU4Hr1G070070 | 0,95 | 4,50 | alpha/beta hydrolase fold-containing protein |
| HORVU1Hr1G044770 | 1,43 | 5,29 | alpha/beta hydrolase fold-containing protein |
| HORVU1Hr1G044740 | 1,75 | 2,95 | alpha/beta hydrolase fold-containing protein |
| HORVU3Hr1G081570 | -1,57 | 10,63 | amino acid transporter |
| HORVU7Hr1G082330 | -1,05 | 2,55 | aminotransferase class III |
| HORVU5Hr1G072920 | 1,94 | 2,40 | AN1-type zinc finger protein |
| HORVU3Hr1G105860 | 1,52 | 2,56 | annexin |
| HORVU3Hr1G089160 | 1,18 | 7,27 | AP2-like factor, ANT lineage |
| HORVU7Hr1G111060 | 1,19 | 8,31 | AP2-like factor, ANT lineage |
| HORVU3Hr1G060080 | -0,65 | 2,03 | apoptosis inhibitor 5-related |
| HORVU2Hr1G096360 | -1,26 | 6,43 | aquaporin PIP |
| HORVU2Hr1G089940 | 1,54 | 4,64 | aquaporin PIP |
| HORVU6Hr1G077070 | 0,71 | 3,04 | arabidopsis histidine kinase 2/3/4 (cytokinin receptor) [EC:2.7.13.3] |
| HORVU3Hr1G094870 | 1,49 | 6,38 | arabidopsis histidine kinase 2/3/4 (cytokinin receptor) [EC:2.7.13.3] |
| HORVU2Hr1G118550 | 1,55 | 2,33 | asparagine synthetase; glutamine amidotransferase |
| HORVU2Hr1G118570 | 1,63 | 2,36 | asparagine synthetase; glutamine amidotransferase |
| HORVU3Hr1G022510 | -0,73 | 10,59 | aspartyl proteases |
| HORVU3Hr1G006450 | 0,40 | 8,87 | aspartyl proteases |
| HORVU3Hr1G100400 | 0,50 | 9,35 | aspartyl proteases |
| HORVU4Hr1G017390 | 0,55 | 2,17 | aspartyl proteases |
| HORVU7Hr1G019770 | 0,96 | 8,12 | aspartyl proteases |
| HORVU3Hr1G006440 | 1,03 | 8,69 | aspartyl proteases |
| HORVU3Hr1G052320 | 1,34 | 8,67 | aspartyl proteases |
| HORVU1Hr1G017570 | -0,33 | 3,99 | aspartyl proteases; phytepsin [EC:3.4.23.40] |
| HORVU3Hr1G022800 | -0,22 | 3,77 | ATP-binding cassette transporter |
| HORVU5Hr1G056280 | 0,48 | 3,36 | ATP-binding cassette transporter |
| HORVU1Hr1G030200 | 1,24 | 2,49 | ATP-binding cassette transporter |
| HORVU1Hr1G009920 | 1,24 | 3,55 | ATP-binding cassette transporter |
| HORVU2Hr1G115180 | -0,67 | 6,56 | ATP-binding cassette, subfamily B (MDR/TAP), member 1 |
| HORVU1Hr1G017460 | 0,72 | 2,83 | ATP-binding cassette, subfamily B (MDR/TAP), member 1 |
| HORVU3Hr1G038610 | -0,67 | 5,98 | ATP-dependent Clp protease, protease subunit [EC:3.4.21.92] |
| HORVU4Hr1G053660 | 0,59 | 2,88 | ATP-dependent Clp protease, protease subunit [EC:3.4.21.92] |
| HORVU7Hr1G017670 | 1,25 | 3,38 | ATP-dependent Clp protease, protease subunit [EC:3.4.21.92] |
| HORVU4Hr1G024950 | -0,60 | 2,09 | ATP-dependent Clp protease |
| HORVU7Hr1G084210 | 1,97 | 5,45 | ATP-dependent RNA helicase [EC:3.6.4.13] |
| HORVU4Hr1G090840 | -1,37 | 3,18 | ATP-dependent RNA helicase DDX18/HAS1 [EC:3.6.4.13] |
| HORVU1Hr1G076690 | 0,71 | 6,94 | auxin response factor |
| HORVU3Hr1G074230 | 1,25 | 9,12 | auxin responsive GH3 gene family |
| HORVU7Hr1G026970 | -0,25 | 11,19 | auxin-responsive protein IAA |
| HORVU1Hr1G086070 | -0,25 | 5,00 | auxin-responsive protein IAA |
| HORVU1Hr1G025670 | 1,48 | 6,84 | auxin-responsive protein IAA |
| HORVU2Hr1G046240 | -0,37 | 2,46 | BCS1 AAA-type ATPase |
| HORVU5Hr1G035300 | -0,14 | 2,48 | BCS1 AAA-type ATPase |
| HORVU2Hr1G046290 | 0,67 | 4,82 | BCS1 AAA-type ATPase |
| HORVU2Hr1G099100 | -1,48 | 2,24 | beta catenin-related armadillo repeat-containing |
| HORVU1Hr1G069990 | 0,85 | 2,42 | beta catenin-related armadillo repeat-containing |
| HORVU5Hr1G067750 | 1,73 | 2,34 | beta-1,3-N-acetylglucosaminyltransferase |
| HORVU1Hr1G055140 | 1,17 | 4,43 | beta-amylase [EC:3.2.1.2] |
| HORVU7Hr1G104660 | 1,29 | 8,25 | beta-carotene 15,15'-monooxygenase [EC:1.14.99.36] |
| HORVU3Hr1G081960 | 0,19 | 5,11 | beta-galactosidase related |
| HORVU4Hr1G074280 | 0,92 | 4,43 | beta-galactosidase related |
| HORVU5Hr1G095130 | -0,96 | 4,46 | beta-glucosidase [EC:3.2.1.21] |
| HORVU2Hr1G088980 | -0,39 | 2,16 | beta-glucosidase [EC:3.2.1.21] |
| HORVU3Hr1G097010 | 0,06 | 2,81 | beta-glucosidase [EC:3.2.1.21] |
| HORVU4Hr1G006480 | 0,97 | 4,39 | beta-glucosidase [EC:3.2.1.21] |
| HORVU7Hr1G038440 | -0,83 | 2,17 | beta-mannan synthase [EC:2.4.1.32] |
| HORVU0Hr1G002630 | -1,17 | 8,10 | BRCA1-associated RING domain protein 1 [EC:6.3.2.19] |
| HORVU1Hr1G069020 | -0,90 | 2,33 | BRCA1-associated RING domain protein 1 [EC:6.3.2.19] |
| HORVU5Hr1G043640 | -0,31 | 3,29 | Ca2+-transporting ATPase [EC:3.6.3.8] |
| HORVU5Hr1G018160 | 0,16 | 2,95 | Ca2+-transporting ATPase [EC:3.6.3.8] |
| HORVU6Hr1G057680 | -0,90 | 3,64 | calcium-activated chloride channel regulator |
| HORVU3Hr1G007420 | -0,20 | 3,69 | calcium-binding protein CML |
| HORVU6Hr1G014700 | -0,16 | 2,25 | calcium-dependent protein kinase [EC:2.7.11.1] |
| HORVU3Hr1G055730 | 1,04 | 2,98 | calcium-dependent protein kinase [EC:2.7.11.1] |
| HORVU2Hr1G051690 | -0,16 | 2,17 | calmodulin-binding transcription activator (CAMTA) |
| HORVU6Hr1G069450 | -0,50 | 2,91 | calpain |
| HORVU3Hr1G089830 | 1,80 | 3,21 | calreticulin |
| HORVU2Hr1G072210 | 1,97 | 4,48 | carboxycyclohexadienyl dehydratase [EC:4.2.1.91] |
| HORVU4Hr1G010390 | -1,59 | 4,75 | cathepsin B [EC:3.4.22.1] |
| HORVU5Hr1G082810 | -0,35 | 2,23 | cathepsin F [EC:3.4.22.41] |
| HORVU2Hr1G066680 | 1,16 | 3,06 | cathepsin F [EC:3.4.22.41] |
| HORVU3Hr1G086970 | 0,82 | 2,55 | cation efflux protein / zinc transportor |
| HORVU6Hr1G072110 | 0,19 | 10,72 | CCAAT-binding transcription factor-related |
| HORVU3Hr1G089130 | -0,35 | 2,13 | CGI-141-related/lipase containing protein |
| HORVU5Hr1G060060 | -0,34 | 2,53 | CGI-141-related/lipase containing protein |
| HORVU7Hr1G041570 | 0,87 | 2,15 | CGI-141-related/lipase containing protein |
| HORVU6Hr1G088200 | 0,84 | 2,45 | chaperone-activity of BC1 complex (CABC1)-related |
| HORVU7Hr1G006490 | -1,41 | 2,29 | chaperonin GroEL |
| HORVU2Hr1G007840 | -1,09 | 4,14 | chaperonin; rubisco subunit binding-protein alpha subunit, ruba |
| HORVU1Hr1G062030 | 1,41 | 4,24 | chitinase [EC:3.2.1.14] |
| HORVU1Hr1G052430 | 1,47 | 5,07 | chitinase-related |
| HORVU1Hr1G059890 | 1,86 | 4,39 | chlorophyll synthase [EC:2.5.1.62] |
| HORVU7Hr1G030380 | 1,23 | 2,67 | cinnamoyl-CoA reductase [EC:1.2.1.44] |
| HORVU5Hr1G113010 | -0,56 | 3,28 | cofilin |
| HORVU3Hr1G089480 | -0,12 | 2,06 | cohesin complex subunit SCC1 |
| HORVU3Hr1G011560 | -1,68 | 3,19 | complement component 1 |
| HORVU6Hr1G010680 | -1,19 | 2,50 | copine |
| HORVU1Hr1G024460 | -0,20 | 2,72 | copine |
| HORVU1Hr1G036920 | -0,11 | 2,06 | copper transport protein ATOX1-related |
| HORVU5Hr1G095480 | 0,03 | 6,13 | copper transport protein ATOX1-related |
| HORVU7Hr1G118340 | 0,77 | 4,58 | copper transport protein ATOX1-related |
| HORVU4Hr1G072060 | 1,40 | 2,35 | copper transport protein ATOX1-related |
| HORVU4Hr1G025770 | 1,48 | 6,99 | copper transport protein ATOX1-related |
| HORVU3Hr1G085210 | -1,97 | 4,24 | coronatine-insensitive protein 1 |
| HORVU1Hr1G073230 | -0,17 | 3,86 | coronatine-insensitive protein 1 |
| HORVU2Hr1G079220 | -0,12 | 2,13 | cryptochrome 1 |
| HORVU1Hr1G077290 | 0,41 | 4,14 | CT120 protein |
| HORVU2Hr1G122730 | -0,38 | 4,50 | ctl transporter |
| HORVU5Hr1G050270 | 0,48 | 7,33 | cyclin D3, plant |
| HORVU5Hr1G046190 | 0,79 | 2,24 | cyclin-dependent kinase [EC:2.7.11.22] |
| HORVU1Hr1G040110 | 0,03 | 2,13 | cyclin-dependent kinase inhibitor 1 |
| HORVU2Hr1G030130 | 0,19 | 2,86 | cycline |
| HORVU5Hr1G114900 | 0,66 | 2,83 | cycloeucalenol cycloisomerase [EC:5.5.1.9] |
| HORVU4Hr1G036280 | -1,56 | 2,11 | cystathionine gamma-synthase [EC:2.5.1.48] |
| HORVU3Hr1G070020 | 0,38 | 2,50 | cystatin family member |
| HORVU3Hr1G088270 | 1,41 | 8,64 | cysteine protease family C1-related |
| HORVU3Hr1G088080 | 1,83 | 9,02 | cysteine protease family C1-related |
| HORVU2Hr1G091720 | 0,50 | 3,91 | cytochrome b-561 |
| HORVU2Hr1G081650 | -1,47 | 10,78 | cytochrome P450, family 724, subfamily B, polypeptide 1 [EC:1.14.13.-] |
| HORVU3Hr1G019850 | 0,91 | 7,53 | cytokinin dehydrogenase [EC:1.5.99.12] |
| HORVU4Hr1G051250 | 0,79 | 2,59 | dedicator of cytokinesis |
| HORVU7Hr1G120030 | -0,64 | 4,24 | delta24-sterol reductase [EC:1.3.1.72] |
| HORVU2Hr1G020240 | 0,60 | 2,15 | developmentally regulated GTP-binding protein-related |
| HORVU5Hr1G106790 | -1,01 | 2,62 | developmentally regulated GTP-binding protein-related; mitochondrial GTPase 2(yeast)/OBG-related |
| HORVU6Hr1G083620 | -0,43 | 2,26 | diacylglycerol kinase [EC:2.7.1.107] |
| HORVU6Hr1G073660 | 1,49 | 6,04 | diphosphoinositol-polyphosphate diphosphatase [EC:3.6.1.52] |
| HORVU6Hr1G095380 | 1,15 | 5,96 | DNA integrity scanning protein |
| HORVU7Hr1G076850 | 0,01 | 5,02 | DNA-directed RNA polymerase I subunit 2 |
| HORVU5Hr1G023090 | -0,46 | 3,34 | DNA-directed RNA polymerase II subunit RPB9 |
| HORVU3Hr1G083740 | -1,88 | 2,28 | DNAJ homolog subfamily C member |
| HORVU3Hr1G010620 | -0,83 | 2,35 | DNAJ homolog subfamily C member |
| HORVU7Hr1G116960 | 0,83 | 2,20 | DNAJ homolog subfamily C member |
| HORVU3Hr1G010600 | 0,95 | 3,05 | DNAJ homolog subfamily C member |
| HORVU4Hr1G061820 | 1,60 | 8,55 | dynamin |
| HORVU5Hr1G065280 | -1,12 | 2,02 | E3 ubiquitin-protein ligase RNF144 [EC:6.3.2.19] |
| HORVU6Hr1G073610 | -1,50 | 3,00 | EF-hand calcium-binding domain containing protein |
| HORVU7Hr1G101740 | -0,14 | 2,26 | EF-hand calcium-binding domain containing protein |
| HORVU4Hr1G022930 | 0,22 | 2,89 | elongation factor EF-P |
| HORVU5Hr1G024470 | -0,06 | 3,02 | elongation factor EF-Ts |
| HORVU3Hr1G029470 | 1,30 | 7,26 | endo-1,4-beta-glucanase |
| HORVU5Hr1G019300 | -1,15 | 2,06 | endoribonuclease Dicer [EC:3.1.26.-] |
| HORVU7Hr1G037180 | -0,66 | 4,83 | EREBP-like factor |
| HORVU6Hr1G074970 | 0,76 | 4,32 | EREBP-like factor |
| HORVU6Hr1G062380 | 1,69 | 3,73 | EREBP-like factor |
| HORVU1Hr1G022550 | 1,31 | 2,59 | ethylene receptor [EC:2.7.13.-] |
| HORVU3Hr1G096250 | -0,78 | 3,52 | euchromatic histone-lysine N-methyltransferase [EC:2.1.1.43] |
| HORVU5Hr1G045170 | 0,12 | 6,63 | euchromatic histone-lysine N-methyltransferase [EC:2.1.1.43] |
| HORVU3Hr1G038830 | -1,65 | 2,42 | eukaryotic translation initiation factor 2C |
| HORVU0Hr1G005350 | -1,21 | 6,37 | eukaryotic translation initiation factor 2C |
| HORVU1Hr1G095300 | 0,89 | 2,72 | eukaryotic translation initiation factor 2C |
| HORVU6Hr1G073590 | 1,84 | 2,10 | eukaryotic translation initiation factor SUI1 |
| HORVU1Hr1G070390 | -0,24 | 2,66 | exocyst complex protein EXO70 |
| HORVU3Hr1G096140 | -0,01 | 2,20 | exostosin (heparan sulfate glycosyltransferase)-related |
| HORVU7Hr1G044270 | -0,76 | 2,81 | extended synaptotagmin-related |
| HORVU2Hr1G025580 | 0,10 | 2,29 | extended synaptotagmin-related |
| HORVU7Hr1G042390 | 0,23 | 2,00 | extensin, proline-rich protein |
| HORVU3Hr1G091200 | 1,15 | 2,62 | F-box and leucine-rich repeat protein 1 (S-phase kinase-associated protein 2) |
| HORVU1Hr1G065030 | 1,23 | 2,52 | F-box and leucine-rich repeat protein 1 (S-phase kinase-associated protein 2) |
| HORVU3Hr1G079270 | 1,18 | 2,81 | FAD NAD binding oxidoreductase |
| HORVU4Hr1G001450 | -0,02 | 9,56 | fatty acyl-CoA reductase [EC:1.2.1.-]; male sterility protein 2 -related |
| HORVU1Hr1G031230 | -1,68 | 4,20 | ferrochelatase [EC:4.99.1.1] |
| HORVU6Hr1G076870 | -1,44 | 3,43 | formin-related |
| HORVU1Hr1G002300 | -0,93 | 5,79 | formin-related |
| HORVU6Hr1G085030 | 0,49 | 2,30 | formin-related |
| HORVU5Hr1G032980 | 1,04 | 8,61 | fructose-bisphosphate aldolase, class I [EC:4.1.2.13] |
| HORVU1Hr1G060810 | 0,68 | 5,94 | gibberellin receptor GID1 [EC:3.-.-.-] |
| HORVU5Hr1G057590 | -0,08 | 3,60 | GLUCOSE-METHANOL-CHOLINE (GMC) OXIDOREDUCTASE |
| HORVU7Hr1G039800 | -0,40 | 2,89 | GLUCOSYL/GLUCURONOSYL TRANSFERASES |
| HORVU0Hr1G040210 | 0,59 | 3,94 | GLUCOSYL/GLUCURONOSYL TRANSFERASES |
| HORVU5Hr1G096310 | 1,06 | 4,14 | GLUCOSYL/GLUCURONOSYL TRANSFERASES |
| HORVU3Hr1G070790 | 0,22 | 2,35 | glutamate carboxypeptidase II [EC:3.4.17.21] |
| HORVU4Hr1G008700 | 1,16 | 3,26 | glutamate decarboxylase [EC:4.1.1.15] |
| HORVU4Hr1G061100 | -0,17 | 5,08 | glutamate dehydrogenase |
| HORVU1Hr1G015590 | -0,32 | 2,48 | glutamate--cysteine ligase [EC:6.3.2.2] |
| HORVU1Hr1G038060 | 0,44 | 3,83 | glutamine synthetase |
| HORVU4Hr1G081100 | -0,18 | 2,60 | glutathione S-transferase [EC:2.5.1.18] |
| HORVU4Hr1G057890 | 0,35 | 3,49 | glutathione S-transferase [EC:2.5.1.18] |
| HORVU2Hr1G045200 | 0,37 | 4,74 | glutathione S-transferase [EC:2.5.1.18] |
| HORVU2Hr1G124330 | 1,31 | 6,67 | glutathione S-transferase [EC:2.5.1.18] |
| HORVU1Hr1G021150 | 1,72 | 6,54 | glutathione S-transferase [EC:2.5.1.18] |
| HORVU7Hr1G108570 | 1,86 | 4,68 | glutathione S-transferase [EC:2.5.1.18] |
| HORVU5Hr1G116920 | 1,53 | 5,60 | glycerol-3-phosphate acyltransferase [EC:2.3.1.15] |
| HORVU2Hr1G023650 | 1,65 | 6,86 | glycerol-3-phosphate acyltransferase [EC:2.3.1.15] |
| HORVU3Hr1G104790 | -0,12 | 4,66 | glycerol-3-phosphate dehydrogenase (NAD+) [EC:1.1.1.8] |
| HORVU2Hr1G097240 | 0,96 | 2,08 | glycogenin |
| HORVU1Hr1G054100 | 1,89 | 2,35 | glycogenin |
| HORVU1Hr1G014230 | 1,15 | 2,25 | Glycosyl hydrolase 43 family member |
| HORVU3Hr1G074490 | 1,95 | 2,20 | Glycosyl hydrolase 43 family member |
| HORVU3Hr1G055670 | -0,25 | 2,70 | glycosyltransferase |
| HORVU3Hr1G003040 | 0,52 | 5,70 | glycosyltransferase |
| HORVU3Hr1G003140 | 0,64 | 5,48 | glycosyltransferase |
| HORVU5Hr1G053930 | 0,97 | 4,74 | glycosyltransferase |
| HORVU5Hr1G013550 | 1,25 | 5,26 | glycosyltransferase |
| HORVU2Hr1G102460 | 1,47 | 5,98 | glycosyltransferase |
| HORVU3Hr1G007960 | 0,15 | 5,73 | GMP synthase-related |
| HORVU3Hr1G007970 | 1,68 | 5,32 | GMP synthase-related |
| HORVU2Hr1G076870 | -0,24 | 8,40 | GTPase, imap family member-related |
| HORVU3Hr1G099760 | -0,29 | 9,72 | gulonolactone oxidase |
| HORVU5Hr1G000780 | 0,74 | 3,19 | H+-transporting ATPase [EC:3.6.3.6] |
| HORVU1Hr1G028200 | 0,12 | 3,89 | hexokinase [EC:2.7.1.1] |
| HORVU1Hr1G064700 | -1,45 | 5,36 | hexosaminidase [EC:3.2.1.52] |
| HORVU2Hr1G038140 | 0,73 | 3,50 | histidine ammonia-lyase |
| HORVU3Hr1G112520 | -0,27 | 9,91 | histidine decarboxylase [EC:4.1.1.22] |
| HORVU1Hr1G074110 | -0,64 | 2,29 | histone deacetylase |
| HORVU1Hr1G095130 | -1,79 | 3,03 | HISTONE H1/H5 |
| HORVU7Hr1G112470 | -1,38 | 2,59 | histone H2A |
| HORVU1Hr1G005870 | -1,28 | 2,90 | histone H2A |
| HORVU1Hr1G035130 | -1,11 | 2,22 | histone H2A |
| HORVU7Hr1G100100 | -0,80 | 2,20 | histone H2A |
| HORVU6Hr1G011490 | -0,34 | 2,16 | histone H2A |
| HORVU7Hr1G030120 | -0,04 | 2,24 | histone H2A |
| HORVU6Hr1G009020 | 0,38 | 2,87 | histone H2A |
| HORVU6Hr1G029220 | 0,70 | 3,01 | histone H2A |
| HORVU3Hr1G087170 | -1,02 | 2,32 | histone H2A; histone H4 |
| HORVU1Hr1G085540 | -1,86 | 3,08 | histone H2B |
| HORVU1Hr1G078530 | -1,72 | 2,41 | histone H2B |
| HORVU1Hr1G058500 | -1,38 | 2,17 | histone H2B |
| HORVU4Hr1G073130 | -1,16 | 2,39 | histone H2B |
| HORVU7Hr1G024990 | -1,98 | 2,19 | histone H3 |
| HORVU1Hr1G073670 | -1,83 | 2,22 | histone H3 |
| HORVU7Hr1G025330 | -1,71 | 2,21 | histone H3 |
| HORVU4Hr1G073150 | -1,60 | 2,07 | histone H3 |
| HORVU1Hr1G080190 | -1,48 | 2,00 | histone H3 |
| HORVU7Hr1G032270 | -1,44 | 2,58 | histone H3 |
| HORVU3Hr1G063270 | -1,40 | 2,10 | histone H3 |
| HORVU1Hr1G022400 | -1,40 | 2,75 | histone H3 |
| HORVU1Hr1G058490 | -1,31 | 2,27 | histone H3 |
| HORVU7Hr1G025160 | -1,23 | 2,49 | histone H3 |
| HORVU7Hr1G100450 | -1,14 | 2,09 | histone H3 |
| HORVU6Hr1G031580 | -0,82 | 2,68 | histone H3 |
| HORVU7Hr1G021840 | -1,27 | 2,90 | histone H3;cysteine synthase A [EC:2.5.1.47] |
| HORVU6Hr1G013530 | -1,87 | 2,09 | histone H4 |
| HORVU5Hr1G087830 | -1,84 | 2,00 | histone H4 |
| HORVU1Hr1G080200 | -1,67 | 2,03 | histone H4 |
| HORVU6Hr1G011710 | -1,10 | 2,16 | histone H4 |
| HORVU1Hr1G052030 | -1,06 | 2,12 | histone H4 |
| HORVU6Hr1G029210 | -0,87 | 2,37 | histone H4 |
| HORVU3Hr1G023460 | -0,81 | 2,11 | histone H4 |
| HORVU2Hr1G097990 | -0,72 | 2,66 | histone H4 |
| HORVU1Hr1G029090 | -0,62 | 2,12 | histone H4 |
| HORVU6Hr1G011020 | 0,47 | 2,32 | histone H4 |
| HORVU4Hr1G003800 | 0,09 | 2,83 | homeobox protein transcription factor |
| HORVU5Hr1G001090 | 1,74 | 7,19 | homeobox protein transcription factor |
| HORVU7Hr1G073440 | -0,51 | 9,17 | homeobox-leucine zipper protein |
| HORVU6Hr1G079410 | 0,01 | 7,50 | homeobox-leucine zipper protein |
| HORVU5Hr1G080700 | 0,73 | 6,76 | homeobox-leucine zipper protein |
| HORVU6Hr1G065300 | 1,40 | 5,68 | homeobox-leucine zipper protein |
| HORVU5Hr1G067010 | 1,49 | 4,35 | homeobox-leucine zipper protein |
| HORVU3Hr1G027500 | 0,21 | 2,53 | homo-oligomeric flavin containing cys decarboxylase family |
| HORVU5Hr1G098090 | 0,09 | 2,37 | hydroperoxide dehydratase [EC:4.2.1.92] |
| HORVU4Hr1G066270 | 0,69 | 3,71 | hydroperoxide dehydratase [EC:4.2.1.92] |
| HORVU1Hr1G017700 | 1,15 | 2,57 | hydrophorbic protein RCI2 (low temperature and salt responsive protein LTI6)-related |
| HORVU7Hr1G030530 | 1,25 | 2,21 | hydrophorbic protein RCI2 (low temperature and salt responsive protein LTI6)-related |
| HORVU2Hr1G090000 | 1,66 | 3,81 | IAA-amino acid hydrolase [EC:3.5.1.-] |
| HORVU4Hr1G011280 | 0,61 | 2,20 | inosine-5-monophosphate dehydrogenase related |
| HORVU6Hr1G081290 | 1,10 | 2,04 | interleukin-1 receptor-associated kinase 4 [EC:2.7.11.1] |
| HORVU7Hr1G078730 | 1,63 | 3,48 | interleukin-1 receptor-associated kinase 4 [EC:2.7.11.1] |
| HORVU2Hr1G045340 | -1,36 | 4,26 | isocitrate lyase [EC:4.1.3.1] |
| HORVU2Hr1G069590 | 0,82 | 2,17 | isocitrate lyaseI/malate synthase |
| HORVU1Hr1G013820 | 1,85 | 2,11 | isovaleryl-CoA dehydrogenase [EC:1.3.99.10] |
| HORVU1Hr1G092890 | -0,03 | 2,87 | jasmonic acid-amino synthetase |
| HORVU3Hr1G040720 | 0,50 | 9,11 | kelch repeat domain |
| HORVU4Hr1G054600 | 1,46 | 2,22 | kinesin family member C2/C3 |
| HORVU4Hr1G057450 | -1,07 | 3,21 | large subunit ribosomal protein L10 |
| HORVU4Hr1G084830 | 0,73 | 2,37 | large subunit ribosomal protein L11 |
| HORVU3Hr1G071530 | 0,26 | 2,04 | large subunit ribosomal protein L13 |
| HORVU6Hr1G062040 | -0,57 | 2,64 | large subunit ribosomal protein L19 |
| HORVU3Hr1G095330 | -0,26 | 2,03 | large subunit ribosomal protein L27 |
| HORVU1Hr1G000040 | 1,39 | 5,05 | large subunit ribosomal protein L28 |
| HORVU6Hr1G018830 | -0,12 | 3,87 | large subunit ribosomal protein L3 |
| HORVU3Hr1G056580 | -1,34 | 3,94 | large subunit ribosomal protein L31 |
| HORVU7Hr1G114170 | 1,20 | 3,63 | large subunit ribosomal protein L35 |
| HORVU4Hr1G040950 | 0,31 | 3,03 | large subunit ribosomal protein L6 |
| HORVU3Hr1G059810 | -0,24 | 2,09 | large subunit ribosomal protein L7/L12 |
| HORVU6Hr1G093030 | 0,63 | 2,96 | large subunit ribosomal protein L9 |
| HORVU2Hr1G098800 | -0,98 | 2,14 | lecithin-cholesterol acyltransferase-related |
| HORVU3Hr1G048520 | -0,63 | 2,00 | legumain [EC:3.4.22.34] |
| HORVU5Hr1G066250 | 0,02 | 7,38 | legumain [EC:3.4.22.34] |
| HORVU5Hr1G018350 | -0,19 | 3,89 | leucine-rich repeat-containing protein |
| HORVU3Hr1G006320 | -1,10 | 2,12 | leucyl-tRNA synthetase [EC:6.1.1.4] |
| HORVU7Hr1G050670 | -0,20 | 5,72 | lipoxygenase |
| HORVU7Hr1G050660 | 0,38 | 5,31 | lipoxygenase |
| HORVU4Hr1G005920 | 1,93 | 2,87 | lipoxygenase |
| HORVU7Hr1G050680 | 0,82 | 4,47 | lipoxygenase [EC:1.13.11.12] |
| HORVU4Hr1G076570 | 1,28 | 2,18 | lipoxygenase [EC:1.13.11.12] |
| HORVU3Hr1G059140 | 0,71 | 3,79 | long-chain acyl-CoA synthetase [EC:6.2.1.3] |
| HORVU4Hr1G025820 | 1,49 | 2,68 | long-chain acyl-CoA synthetase [EC:6.2.1.3] |
| HORVU5Hr1G076400 | -1,56 | 2,16 | mads box protein |
| HORVU2Hr1G063800 | 0,50 | 5,18 | mads-box transcription factor, plant |
| HORVU0Hr1G003910 | -0,33 | 2,25 | maternally affected uncoordination |
| HORVU5Hr1G095530 | -0,50 | 2,45 | meiotic recombination protein SPO11 |
| HORVU5Hr1G069040 | -0,15 | 5,35 | member of 'GDXG' family of lipolytic enzymes |
| HORVU5Hr1G017510 | -0,10 | 3,01 | member of 'GDXG' family of lipolytic enzymes |
| HORVU6Hr1G005900 | 1,10 | 6,88 | member of 'GDXG' family of lipolytic enzymes |
| HORVU3Hr1G022640 | 1,55 | 4,38 | member of 'GDXG' family of lipolytic enzymes |
| HORVU3Hr1G085590 | -0,29 | 2,00 | membrane associated ring finger |
| HORVU2Hr1G127190 | -0,10 | 2,58 | membrane associated ring finger |
| HORVU0Hr1G000340 | 0,58 | 5,14 | methionine-gamma-lyase [EC:4.4.1.11] |
| HORVU2Hr1G099850 | 0,17 | 3,18 | methionyl aminopeptidase [EC:3.4.11.18] |
| HORVU2Hr1G047450 | 0,59 | 2,13 | methionyl aminopeptidase [EC:3.4.11.18] |
| HORVU3Hr1G056960 | -1,03 | 2,87 | methyltetrahydrofolate:homocysteine methyltransferase |
| HORVU4Hr1G011120 | -1,94 | 2,06 | methyltransferase |
| HORVU3Hr1G090970 | -0,83 | 5,16 | methyltransferase |
| HORVU2Hr1G127220 | 0,20 | 2,43 | methyltransferase |
| HORVU1Hr1G091460 | 1,78 | 2,03 | MFS transporter, SP family, solute carrier family 2 (facilitated glucose transporter), member 8 |
| HORVU1Hr1G029770 | -1,50 | 2,09 | minichromosome maintenance protein 6; DNA replication licenssing factor MCM6 |
| HORVU6Hr1G065800 | -0,28 | 2,12 | MKIAA1688 protein |
| HORVU5Hr1G057800 | -0,25 | 3,36 | multi-copper oxidase |
| HORVU7Hr1G049240 | 0,78 | 5,63 | multidrug resistance protein, MATE family |
| HORVU1Hr1G053310 | -0,46 | 2,70 | mur ligase family member |
| HORVU5Hr1G018220 | 0,94 | 2,66 | myb family transcription factor |
| HORVU2Hr1G028470 | 1,27 | 7,41 | myb proto-oncogene protein, plant |
| HORVU2Hr1G089310 | -1,82 | 3,41 | myb-like DNA-binding protein myb |
| HORVU4Hr1G002350 | 1,31 | 3,68 | N utilization substance protein B-related |
| HORVU3Hr1G019440 | -0,01 | 2,56 | N-myc downstream regulated |
| HORVU7Hr1G113480 | 1,53 | 5,39 | N-terminal acetyltransferase |
| HORVU1Hr1G050830 | 0,01 | 2,71 | NAD dependent epimerase/dehydratase |
| HORVU7Hr1G030500 | 1,49 | 4,59 | NAD dependent epimerase/dehydratase |
| HORVU3Hr1G037600 | -0,28 | 3,34 | NADPH oxidase |
| HORVU6Hr1G088160 | 1,22 | 2,09 | NADPH2:quinone reductase [EC:1.6.5.5] |
| HORVU4Hr1G069320 | 1,82 | 3,06 | natural resistance-associated macrophage protein |
| HORVU2Hr1G015980 | 1,34 | 7,54 | nicotianamine synthase [EC:2.5.1.43] |
| HORVU4Hr1G055070 | 0,32 | 3,62 | nitrate,formate,iron dehydrogenase |
| HORVU2Hr1G065330 | 1,89 | 7,49 | nitrate,formate,iron dehydrogenase |
| HORVU6Hr1G081080 | -0,38 | 3,06 | nuclear transcription factor Y, alpha |
| HORVU2Hr1G032130 | 0,76 | 2,12 | nuclear transcription factor Y, alpha |
| HORVU1Hr1G089700 | -1,79 | 7,81 | O-methyltransferase |
| HORVU7Hr1G048970 | 1,20 | 7,13 | O-methyltransferase |
| HORVU4Hr1G004460 | 1,52 | 3,13 | oligopeptide transporter-related |
| HORVU3Hr1G096860 | 1,08 | 2,53 | osmotic stress potassium transporter; KUP system potassium uptake protein |
| HORVU4Hr1G084810 | 0,54 | 4,73 | oxidoreductase, 2OG-FE(II) oxygenase family protein |
| HORVU4Hr1G013840 | 1,70 | 4,68 | oxidoreductase, 2OG-FE(II) oxygenase family protein |
| HORVU1Hr1G015050 | 1,83 | 2,43 | oxidoreductase, 2OG-FE(II) oxygenase family protein |
| HORVU5Hr1G079670 | 1,90 | 5,81 | pathogen-inducible salicylic acid glucosyltransferase [EC:2.4.1.-] |
| HORVU6Hr1G077290 | 0,36 | 2,68 | peptidyl-prolyl isomerase D (cyclophilin D) [EC:5.2.1.8] |
| HORVU4Hr1G088840 | -0,63 | 3,04 | PER1-related |
| HORVU6Hr1G075010 | -0,90 | 6,51 | periplasmic beta-glucosidase-related |
| HORVU2Hr1G115640 | 1,79 | 2,86 | periplasmic beta-glucosidase-related |
| HORVU5Hr1G070290 | -1,49 | 7,65 | peroxidase [EC:1.11.1.7] |
| HORVU2Hr1G124930 | 0,00 | 9,73 | peroxidase [EC:1.11.1.7] |
| HORVU3Hr1G074940 | 0,15 | 7,15 | peroxidase [EC:1.11.1.7] |
| HORVU2Hr1G124970 | 0,22 | 9,27 | peroxidase [EC:1.11.1.7] |
| HORVU4Hr1G022280 | 0,44 | 4,66 | peroxidase [EC:1.11.1.7] |
| HORVU4Hr1G016940 | 0,51 | 9,18 | peroxidase [EC:1.11.1.7] |
| HORVU2Hr1G013740 | 0,82 | 7,71 | peroxidase [EC:1.11.1.7] |
| HORVU5Hr1G046900 | 1,36 | 6,99 | peroxidase [EC:1.11.1.7] |
| HORVU0Hr1G016330 | 0,39 | 3,81 | phenylalanine ammonia-lyase [EC:4.3.1.24] |
| HORVU2Hr1G038120 | 1,15 | 3,56 | phenylalanine ammonia-lyase [EC:4.3.1.24] |
| HORVU5Hr1G071480 | -1,00 | 4,07 | phenylalanyl-tRNA sunthetase |
| HORVU5Hr1G117610 | 1,14 | 2,72 | phenylalanyl-tRNA sunthetase |
| HORVU3Hr1G007200 | 1,48 | 6,46 | phosphatidylinositol N-acetylglucosaminyltransferase subunit P (down syndrome critical region protein 5)-related |
| HORVU5Hr1G067890 | -1,29 | 3,17 | phosphatidylinositol-4-phosphate 5-kinase related |
| HORVU1Hr1G000140 | 1,13 | 2,27 | phospholipid-translocating ATPase [EC:3.6.3.1] |
| HORVU1Hr1G040670 | -0,75 | 2,02 | phosphoribosylformylglycinamidine synthase [EC:6.3.5.3]; tegument protein |
| HORVU7Hr1G038700 | -0,99 | 3,57 | pin; auxin efflux carrier family |
| HORVU2Hr1G056710 | -0,28 | 2,02 | polyribonucleotide nucleotidyltransferase |
| HORVU4Hr1G005110 | 0,27 | 3,48 | pqq oxidoreductase-related |
| HORVU3Hr1G013690 | 0,05 | 2,04 | preprotein translocase subunit YidC |
| HORVU3Hr1G094600 | -0,84 | 2,76 | programmed cell death 2 |
| HORVU7Hr1G074850 | -1,26 | 2,19 | programmed cell death 4 |
| HORVU5Hr1G103450 | -1,74 | 2,30 | prokaryotic DNA topoisomerase |
| HORVU6Hr1G081850 | -1,57 | 3,34 | proprotein convertase subtilisin/kexin |
| HORVU7Hr1G097470 | -0,25 | 4,25 | proprotein convertase subtilisin/kexin |
| HORVU5Hr1G097150 | 0,00 | 7,46 | proprotein convertase subtilisin/kexin |
| HORVU6Hr1G081750 | 0,43 | 9,70 | proprotein convertase subtilisin/kexin |
| HORVU2Hr1G094670 | 1,67 | 4,95 | proprotein convertase subtilisin/kexin |
| HORVU3Hr1G025400 | 0,24 | 3,56 | protease family S9B,C dipeptidyl-peptidase IV-related |
| HORVU1Hr1G084450 | 0,04 | 5,35 | protein phosphatase 2 (formerly 2A), regulatory subunit B' |
| HORVU4Hr1G056210 | -0,26 | 2,52 | protein phosphatase 2C |
| HORVU2Hr1G079200 | 0,30 | 3,16 | protein phosphatase 2C |
| HORVU5Hr1G097060 | 0,41 | 2,32 | protein phosphatase 2C |
| HORVU3Hr1G048950 | 0,79 | 2,07 | protein phosphatase 2C |
| HORVU6Hr1G066850 | 1,12 | 3,00 | protein phosphatase 2C |
| HORVU2Hr1G014150 | 1,20 | 3,30 | protein phosphatase 2C |
| HORVU5Hr1G047410 | 1,59 | 6,24 | protein phosphatase 2C |
| HORVU2Hr1G100100 | 1,91 | 2,38 | protein phosphatase 2C |
| HORVU1Hr1G016640 | 0,83 | 2,03 | protein phosphatase 2C homolog 2/3 [EC:3.1.3.16] |
| HORVU4Hr1G059220 | 0,77 | 4,20 | protein xylosyltransferase [EC:2.4.2.26] |
| HORVU5Hr1G120520 | -0,89 | 2,38 | pto-interacting protein 1 [EC:2.7.11.1] |
| HORVU2Hr1G026340 | -0,76 | 2,41 | pyroglutamyl-peptidase [EC:3.4.19.3] |
| HORVU1Hr1G005090 | 1,94 | 6,05 | pyruvate dehydrogenase phosphatase [EC:3.1.3.43] |
| HORVU5Hr1G053480 | 0,14 | 3,26 | rapsyn-related |
| HORVU7Hr1G089120 | -0,67 | 2,38 | ras-related protein rab-11A |
| HORVU3Hr1G071740 | -0,42 | 2,23 | ras-related protein rab-11A |
| HORVU2Hr1G116340 | -0,29 | 8,16 | RBR family (ring finger and ibr domain-containing) |
| HORVU2Hr1G124270 | -1,42 | 3,18 | regulator of chromosome condensation |
| HORVU2Hr1G076180 | 1,00 | 4,32 | regulator of chromosome condensation |
| HORVU1Hr1G002740 | 1,71 | 2,41 | regulator of chromosome condensation |
| HORVU3Hr1G068780 | -0,07 | 2,75 | regulator of chromosome condensation; nitrate, formate, iron dehydrogenaseNASE |
| HORVU3Hr1G019230 | 0,26 | 5,04 | regulatory protein NPR1 |
| HORVU1Hr1G020660 | 1,25 | 2,64 | replication factor A1, RFA1 |
| HORVU1Hr1G081950 | 0,48 | 2,07 | respiratory burst oxidase [EC:1.6.3.- 1.11.1.-] |
| HORVU4Hr1G090610 | 0,29 | 2,33 | RFWD3 protein |
| HORVU5Hr1G080690 | -1,81 | 4,69 | rhomboid-related |
| HORVU1Hr1G021990 | -0,46 | 2,52 | ribonuclease P protein subunit P38-related |
| HORVU7Hr1G071790 | 1,05 | 2,47 | ribonuclease P protein subunit P38-related |
| HORVU5Hr1G021050 | 1,22 | 6,28 | ribonuclease P protein subunit P38-related |
| HORVU2Hr1G028430 | -1,97 | 3,06 | ribonuclease T2 [EC:3.1.27.1] |
| HORVU7Hr1G027430 | -1,41 | 2,63 | ribonucleoside-diphosphate reductase subunit M1 [EC:1.17.4.1] |
| HORVU2Hr1G038570 | -0,09 | 3,76 | ribosome recycling factor |
| HORVU7Hr1G017880 | -1,72 | 2,25 | ribosome-binding factor A |
| HORVU3Hr1G064370 | 1,05 | 4,31 | ring finger domain-containing |
| HORVU7Hr1G117640 | -0,75 | 3,17 | ring finger domain-containing |
| HORVU0Hr1G040490 | 0,79 | 5,24 | ring finger domain-containing |
| HORVU0Hr1G005330 | 1,28 | 2,73 | ring finger domain-containing |
| HORVU7Hr1G031290 | 1,63 | 3,36 | ring finger domain-containing |
| HORVU6Hr1G022590 | 1,94 | 5,44 | ring finger domain-containing |
| HORVU3Hr1G115020 | -0,01 | 2,38 | RNA binding protein pumilio-related |
| HORVU3Hr1G092200 | -0,60 | 2,67 | RNA splicing protein MRS2, mitochondrial |
| HORVU4Hr1G077840 | 0,05 | 2,95 | RW1 protein homolog |
| HORVU3Hr1G017580 | -1,77 | 3,21 | saposin |
| HORVU5Hr1G093650 | -1,59 | 2,90 | sec-independent protein translocase protein TatA |
| HORVU3Hr1G065830 | -0,49 | 2,08 | SEC14 related protein |
| HORVU3Hr1G082110 | 0,53 | 6,94 | SEC14 related protein |
| HORVU5Hr1G061580 | 1,53 | 9,44 | secretory carrier-associated membrane protein (SCAMP) |
| HORVU3Hr1G093350 | 0,63 | 4,23 | selenium-binding protein |
| HORVU5Hr1G022360 | 0,29 | 2,19 | serine/threonine kinase |
| HORVU5Hr1G093660 | 0,95 | 5,87 | serine/threonine kinase |
| HORVU5Hr1G040090 | -0,62 | 2,30 | serine/threonine-protein kinase wnk (with no lysine)-related |
| HORVU2Hr1G107560 | 0,52 | 2,49 | set domain proteins |
| HORVU3Hr1G018640 | 0,67 | 9,63 | seven in absentia homolog |
| HORVU4Hr1G016950 | 0,97 | 7,60 | seven in absentia homolog |
| HORVU3Hr1G005400 | 0,32 | 2,03 | seven in absentia homolog; sentrin/sumo-specific protease |
| HORVU2Hr1G086380 | -0,55 | 2,09 | shikimate O-hydroxycinnamoyltransferase [EC:2.3.1.133] |
| HORVU4Hr1G021950 | 0,18 | 2,26 | signal recognition particle subunit SRP54 |
| HORVU4Hr1G006730 | -0,10 | 3,78 | small subunit ribosomal protein S13 |
| HORVU7Hr1G115040 | -0,08 | 3,13 | small subunit ribosomal protein S17 |
| HORVU4Hr1G038570 | 0,62 | 2,71 | small subunit ribosomal protein S5 |
| HORVU4Hr1G009620 | -1,00 | 3,09 | solute carrier family 35 |
| HORVU3Hr1G024590 | -0,30 | 3,41 | solute carrier family 35 |
| HORVU5Hr1G112210 | -0,72 | 2,82 | solute carrier family 36 (proton-coupled amino acid transporter), member 1; amino acid transporter |
| HORVU2Hr1G106560 | 1,71 | 8,68 | speckle-type POZ protein |
| HORVU1Hr1G093830 | -1,28 | 2,40 | SSM4 protein |
| HORVU6Hr1G075950 | -1,19 | 3,12 | starch synthase [EC:2.4.1.21] |
| HORVU6Hr1G089980 | 1,09 | 4,66 | sterol desaturase |
| HORVU7Hr1G112710 | 1,72 | 7,65 | sterol desaturase |
| HORVU6Hr1G058000 | 1,91 | 5,31 | sterol desaturase |
| HORVU5Hr1G075430 | 0,42 | 2,84 | sterol regulatory element-binding protein |
| HORVU7Hr1G083390 | 1,99 | 2,96 | sterol regulatory element-binding protein |
| HORVU5Hr1G067880 | -0,70 | 2,33 | subgroup I aminotransferase |
| HORVU1Hr1G072230 | 0,01 | 3,34 | subgroup I aminotransferase |
| HORVU1Hr1G042490 | 0,39 | 4,04 | subgroup I aminotransferase |
| HORVU4Hr1G071210 | 0,95 | 3,13 | sulfate transporter |
| HORVU3Hr1G012850 | -1,05 | 2,21 | SWI/SNF-related matrix-associated actin-dependent regulator of chromatin subfamily A member 5 [EC:3.6.4.-] |
| HORVU5Hr1G073350 | -0,65 | 2,15 | synaptotagmin |
| HORVU4Hr1G002160 | -0,10 | 3,41 | synaptotagmin |
| HORVU1Hr1G003170 | -1,12 | 2,09 | TBC1 domain family member GTPase-activating protein |
| HORVU5Hr1G123330 | 0,49 | 7,47 | thioredoxin |
| HORVU2Hr1G103310 | 0,58 | 2,88 | thioredoxin |
| HORVU5Hr1G123280 | 1,61 | 6,83 | thioredoxin |
| HORVU2Hr1G060510 | 0,46 | 4,12 | toll-linke receptor |
| HORVU2Hr1G015760 | -1,56 | 2,22 | topoisomerase (DNA) II binding protein 1 |
| HORVU3Hr1G027650 | -0,42 | 4,12 | TPR repeat containing protein |
| HORVU3Hr1G054240 | -0,32 | 2,97 | TPR repeat containing protein |
| HORVU2Hr1G020310 | 0,27 | 5,58 | TPR repeat containing protein |
| HORVU5Hr1G086150 | 1,55 | 6,84 | TPR repeat containing protein; ankyrin repeat protein |
| HORVU3Hr1G104830 | 0,48 | 10,63 | TRAF AND TNF receptor-associated protein |
| HORVU2Hr1G093230 | 0,91 | 3,43 | transcription factor GATA (GATA binding factor) |
| HORVU4Hr1G079070 | 1,09 | 2,36 | transcription factor GATA (GATA binding factor) |
| HORVU2Hr1G092420 | 1,45 | 2,72 | transcription factor GATA (GATA binding factor) |
| HORVU3Hr1G079010 | 1,93 | 6,29 | transcription factor TGA |
| HORVU5Hr1G110960 | -1,38 | 3,10 | transcription initiation factor TFIIE subunit beta |
| HORVU1Hr1G038370 | -0,55 | 2,02 | transcriptional regulator ATRX [EC:3.6.4.12] |
| HORVU6Hr1G084190 | -0,81 | 3,72 | trehalose-6-phosphate synthase |
| HORVU5Hr1G058300 | 1,02 | 2,89 | trehalose-phosphatase [EC:3.1.3.12] |
| HORVU6Hr1G085560 | -1,19 | 2,14 | two-component response regulator ARR-B family |
| HORVU4Hr1G066480 | -0,33 | 2,11 | two-component response regulator ARR-B family |
| HORVU3Hr1G081400 | 1,15 | 5,46 | ubiquitin |
| HORVU5Hr1G096370 | -0,55 | 2,54 | UDP glucose 6-dehydrogenase [EC:1.1.1.22] |
| HORVU7Hr1G029200 | -0,39 | 2,99 | uncharacterized nodulin-like protein |
| HORVU7Hr1G053470 | 0,70 | 4,07 | uncharacterized ring zinc finger-containing protein |
| HORVU2Hr1G011640 | -1,96 | 2,31 | unknown |
| HORVU3Hr1G017570 | -1,94 | 2,26 | unknown |
| HORVU2Hr1G002930 | -1,85 | 3,53 | unknown |
| HORVU7Hr1G030870 | -1,85 | 7,11 | unknown |
| HORVU6Hr1G061710 | -1,84 | 4,50 | unknown |
| HORVU6Hr1G064170 | -1,78 | 9,24 | unknown |
| HORVU1Hr1G002170 | -1,76 | 10,38 | unknown |
| HORVU5Hr1G096000 | -1,75 | 4,18 | unknown |
| HORVU6Hr1G054050 | -1,56 | 6,55 | unknown |
| HORVU3Hr1G096360 | -1,54 | 8,83 | unknown |
| HORVU6Hr1G070090 | -1,52 | 8,81 | unknown |
| HORVU6Hr1G083110 | -1,51 | 2,85 | unknown |
| HORVU1Hr1G093170 | -1,51 | 9,30 | unknown |
| HORVU7Hr1G073170 | -1,49 | 2,20 | unknown |
| HORVU2Hr1G061090 | -1,47 | 9,33 | unknown |
| HORVU6Hr1G064470 | -1,46 | 2,61 | unknown |
| HORVU3Hr1G034310 | -1,45 | 6,76 | unknown |
| HORVU2Hr1G080460 | -1,35 | 7,10 | unknown |
| HORVU1Hr1G041770 | -1,31 | 3,06 | unknown |
| HORVU7Hr1G025150 | -1,29 | 3,04 | unknown |
| HORVU5Hr1G017760 | -1,28 | 2,00 | unknown |
| HORVU5Hr1G097320 | -1,28 | 3,17 | unknown |
| HORVU2Hr1G029720 | -1,27 | 3,55 | unknown |
| HORVU4Hr1G074960 | -1,24 | 2,33 | unknown |
| HORVU7Hr1G082560 | -1,23 | 2,73 | unknown |
| HORVU2Hr1G075690 | -1,23 | 7,38 | unknown |
| HORVU7Hr1G099650 | -1,16 | 3,48 | unknown |
| HORVU7Hr1G092680 | -1,14 | 9,71 | unknown |
| HORVU1Hr1G012690 | -1,13 | 6,38 | unknown |
| HORVU5Hr1G092770 | -1,12 | 4,63 | unknown |
| HORVU1Hr1G008480 | -1,12 | 11,03 | unknown |
| HORVU3Hr1G074850 | -1,07 | 2,69 | unknown |
| HORVU5Hr1G104600 | -1,06 | 9,55 | unknown |
| HORVU1Hr1G038620 | -1,04 | 2,39 | unknown |
| HORVU5Hr1G105660 | -1,03 | 2,31 | unknown |
| HORVU6Hr1G089730 | -1,02 | 10,06 | unknown |
| HORVU3Hr1G000600 | -0,99 | 8,67 | unknown |
| HORVU5Hr1G081060 | -0,98 | 5,51 | unknown |
| HORVU4Hr1G027970 | -0,91 | 2,57 | unknown |
| HORVU5Hr1G109680 | -0,90 | 3,21 | unknown |
| HORVU4Hr1G026200 | -0,86 | 7,70 | unknown |
| HORVU2Hr1G085590 | -0,86 | 2,04 | unknown |
| HORVU5Hr1G072770 | -0,86 | 3,97 | unknown |
| HORVU5Hr1G057460 | -0,86 | 2,00 | unknown |
| HORVU6Hr1G038250 | -0,85 | 4,23 | unknown |
| HORVU7Hr1G055890 | -0,82 | 7,30 | unknown |
| HORVU5Hr1G113580 | -0,82 | 5,18 | unknown |
| HORVU4Hr1G073730 | -0,81 | 3,81 | unknown |
| HORVU3Hr1G114340 | -0,80 | 3,21 | unknown |
| HORVU2Hr1G036980 | -0,79 | 2,87 | unknown |
| HORVU7Hr1G025000 | -0,78 | 2,70 | unknown |
| HORVU2Hr1G036570 | -0,76 | 4,19 | unknown |
| HORVU5Hr1G049810 | -0,74 | 2,70 | unknown |
| HORVU5Hr1G097310 | -0,73 | 3,79 | unknown |
| HORVU3Hr1G108950 | -0,72 | 6,64 | unknown |
| HORVU5Hr1G091840 | -0,70 | 7,94 | unknown |
| HORVU6Hr1G031430 | -0,65 | 2,19 | unknown |
| HORVU2Hr1G003750 | -0,65 | 2,11 | unknown |
| HORVU2Hr1G070300 | -0,64 | 2,40 | unknown |
| HORVU6Hr1G012210 | -0,64 | 10,95 | unknown |
| HORVU7Hr1G054530 | -0,62 | 8,98 | unknown |
| HORVU6Hr1G060710 | -0,61 | 2,43 | unknown |
| HORVU5Hr1G083210 | -0,58 | 2,63 | unknown |
| HORVU7Hr1G101980 | -0,57 | 10,51 | unknown |
| HORVU3Hr1G014380 | -0,57 | 2,26 | unknown |
| HORVU4Hr1G071470 | -0,54 | 2,00 | unknown |
| HORVU7Hr1G052580 | -0,51 | 2,22 | unknown |
| HORVU1Hr1G059720 | -0,47 | 2,00 | unknown |
| HORVU3Hr1G109300 | -0,43 | 2,08 | unknown |
| HORVU6Hr1G087420 | -0,40 | 4,53 | unknown |
| HORVU2Hr1G090310 | -0,38 | 6,56 | unknown |
| HORVU7Hr1G116600 | -0,34 | 2,25 | unknown |
| HORVU2Hr1G033880 | -0,34 | 6,79 | unknown |
| HORVU3Hr1G091930 | -0,32 | 8,35 | unknown |
| HORVU5Hr1G048630 | -0,32 | 3,32 | unknown |
| HORVU4Hr1G048970 | -0,32 | 2,11 | unknown |
| HORVU3Hr1G081170 | -0,30 | 6,48 | unknown |
| HORVU6Hr1G058020 | -0,30 | 2,88 | unknown |
| HORVU3Hr1G023750 | -0,29 | 3,16 | unknown |
| HORVU4Hr1G076940 | -0,28 | 8,79 | unknown |
| HORVU1Hr1G049980 | -0,28 | 2,14 | unknown |
| HORVU7Hr1G025840 | -0,28 | 8,33 | unknown |
| HORVU4Hr1G014970 | -0,28 | 3,49 | unknown |
| HORVU5Hr1G075370 | -0,24 | 6,54 | unknown |
| HORVU5Hr1G067650 | -0,24 | 8,07 | unknown |
| HORVU1Hr1G075210 | -0,23 | 2,08 | unknown |
| HORVU5Hr1G078950 | -0,21 | 2,08 | unknown |
| HORVU7Hr1G083180 | -0,21 | 4,10 | unknown |
| HORVU1Hr1G025940 | -0,19 | 2,63 | unknown |
| HORVU5Hr1G050970 | -0,19 | 3,19 | unknown |
| HORVU3Hr1G079800 | -0,18 | 6,34 | unknown |
| HORVU7Hr1G090290 | -0,18 | 2,22 | unknown |
| HORVU6Hr1G033310 | -0,18 | 2,79 | unknown |
| HORVU4Hr1G053580 | -0,17 | 3,08 | unknown |
| HORVU7Hr1G095940 | -0,17 | 9,32 | unknown |
| HORVU7Hr1G099340 | -0,16 | 2,83 | unknown |
| HORVU4Hr1G063420 | -0,15 | 8,04 | unknown |
| HORVU2Hr1G099550 | -0,14 | 7,60 | unknown |
| HORVU1Hr1G077100 | -0,14 | 6,50 | unknown |
| HORVU7Hr1G103180 | -0,13 | 4,06 | unknown |
| HORVU3Hr1G001960 | -0,13 | 2,16 | unknown |
| HORVU7Hr1G036710 | -0,10 | 2,90 | unknown |
| HORVU7Hr1G030460 | -0,09 | 5,69 | unknown |
| HORVU2Hr1G035830 | -0,07 | 7,31 | unknown |
| HORVU6Hr1G060080 | -0,06 | 2,29 | unknown |
| HORVU4Hr1G084510 | -0,05 | 3,73 | unknown |
| HORVU1Hr1G073010 | -0,05 | 4,66 | unknown |
| HORVU0Hr1G003900 | 0,00 | 9,77 | unknown |
| HORVU1Hr1G081140 | 0,00 | 10,74 | unknown |
| HORVU3Hr1G006640 | 0,00 | 10,57 | unknown |
| HORVU4Hr1G086380 | 0,00 | 10,30 | unknown |
| HORVU5Hr1G014510 | 0,00 | 10,66 | unknown |
| HORVU7Hr1G120450 | 0,01 | 10,47 | unknown |
| HORVU3Hr1G028780 | 0,01 | 2,77 | unknown |
| HORVU5Hr1G053230 | 0,03 | 2,09 | unknown |
| HORVU5Hr1G056130 | 0,04 | 2,96 | unknown |
| HORVU3Hr1G050310 | 0,04 | 4,78 | unknown |
| HORVU4Hr1G008300 | 0,04 | 2,29 | unknown |
| HORVU3Hr1G095880 | 0,05 | 9,00 | unknown |
| HORVU7Hr1G076860 | 0,08 | 4,54 | unknown |
| HORVU5Hr1G103400 | 0,10 | 3,31 | unknown |
| HORVU2Hr1G100620 | 0,12 | 3,55 | unknown |
| HORVU2Hr1G115620 | 0,16 | 2,85 | unknown |
| HORVU7Hr1G102010 | 0,16 | 9,84 | unknown |
| HORVU1Hr1G086930 | 0,16 | 2,91 | unknown |
| HORVU4Hr1G026770 | 0,18 | 5,35 | unknown |
| HORVU7Hr1G014410 | 0,19 | 6,73 | unknown |
| HORVU4Hr1G016500 | 0,19 | 9,38 | unknown |
| HORVU2Hr1G039930 | 0,20 | 4,54 | unknown |
| HORVU0Hr1G016430 | 0,20 | 8,02 | unknown |
| HORVU2Hr1G102660 | 0,21 | 2,41 | unknown |
| HORVU1Hr1G005510 | 0,21 | 6,23 | unknown |
| HORVU2Hr1G073590 | 0,21 | 2,74 | unknown |
| HORVU6Hr1G000480 | 0,21 | 7,52 | unknown |
| HORVU7Hr1G052770 | 0,22 | 6,41 | unknown |
| HORVU4Hr1G005320 | 0,22 | 8,14 | unknown |
| HORVU2Hr1G120150 | 0,22 | 2,11 | unknown |
| HORVU1Hr1G047710 | 0,23 | 6,69 | unknown |
| HORVU5Hr1G094460 | 0,25 | 4,53 | unknown |
| HORVU2Hr1G049410 | 0,27 | 4,05 | unknown |
| HORVU7Hr1G070080 | 0,27 | 6,87 | unknown |
| HORVU5Hr1G098840 | 0,29 | 8,41 | unknown |
| HORVU2Hr1G123200 | 0,29 | 4,85 | unknown |
| HORVU3Hr1G081590 | 0,29 | 2,94 | unknown |
| HORVU4Hr1G055410 | 0,30 | 3,26 | unknown |
| HORVU7Hr1G023610 | 0,31 | 2,66 | unknown |
| HORVU2Hr1G113830 | 0,33 | 2,21 | unknown |
| HORVU7Hr1G051430 | 0,35 | 3,65 | unknown |
| HORVU0Hr1G003220 | 0,35 | 2,07 | unknown |
| HORVU2Hr1G082330 | 0,36 | 2,91 | unknown |
| HORVU4Hr1G009990 | 0,38 | 2,13 | unknown |
| HORVU1Hr1G005260 | 0,40 | 4,26 | unknown |
| HORVU1Hr1G068830 | 0,40 | 5,99 | unknown |
| HORVU4Hr1G020080 | 0,41 | 2,34 | unknown |
| HORVU4Hr1G019610 | 0,43 | 2,01 | unknown |
| HORVU5Hr1G065740 | 0,43 | 8,47 | unknown |
| HORVU0Hr1G005320 | 0,43 | 5,41 | unknown |
| HORVU3Hr1G107830 | 0,45 | 8,89 | unknown |
| HORVU5Hr1G009890 | 0,45 | 2,08 | unknown |
| HORVU7Hr1G043550 | 0,46 | 2,12 | unknown |
| HORVU3Hr1G082030 | 0,48 | 7,94 | unknown |
| HORVU5Hr1G057840 | 0,49 | 7,87 | unknown |
| HORVU2Hr1G045970 | 0,52 | 5,51 | unknown |
| HORVU7Hr1G089500 | 0,52 | 5,45 | unknown |
| HORVU3Hr1G094140 | 0,53 | 8,13 | unknown |
| HORVU2Hr1G022080 | 0,54 | 10,08 | unknown |
| HORVU3Hr1G003860 | 0,54 | 4,04 | unknown |
| HORVU3Hr1G034040 | 0,56 | 6,27 | unknown |
| HORVU4Hr1G072370 | 0,56 | 7,53 | unknown |
| HORVU4Hr1G043800 | 0,57 | 2,42 | unknown |
| HORVU3Hr1G083720 | 0,58 | 6,84 | unknown |
| HORVU1Hr1G056710 | 0,59 | 7,58 | unknown |
| HORVU4Hr1G009570 | 0,59 | 2,33 | unknown |
| HORVU4Hr1G025180 | 0,60 | 3,21 | unknown |
| HORVU5Hr1G104970 | 0,60 | 8,22 | unknown |
| HORVU7Hr1G112410 | 0,60 | 2,75 | unknown |
| HORVU0Hr1G017670 | 0,60 | 8,10 | unknown |
| HORVU1Hr1G043580 | 0,61 | 3,67 | unknown |
| HORVU3Hr1G116730 | 0,62 | 7,13 | unknown |
| HORVU1Hr1G054250 | 0,62 | 5,84 | unknown |
| HORVU2Hr1G035810 | 0,62 | 4,98 | unknown |
| HORVU1Hr1G050640 | 0,62 | 2,77 | unknown |
| HORVU2Hr1G042120 | 0,64 | 4,08 | unknown |
| HORVU4Hr1G015000 | 0,65 | 2,10 | unknown |
| HORVU2Hr1G009580 | 0,67 | 3,00 | unknown |
| HORVU1Hr1G019380 | 0,68 | 6,24 | unknown |
| HORVU3Hr1G061690 | 0,70 | 2,98 | unknown |
| HORVU1Hr1G051330 | 0,71 | 4,55 | unknown |
| HORVU4Hr1G068870 | 0,71 | 2,20 | unknown |
| HORVU0Hr1G021800 | 0,72 | 2,34 | unknown |
| HORVU5Hr1G073550 | 0,75 | 4,42 | unknown |
| HORVU0Hr1G025440 | 0,76 | 2,56 | unknown |
| HORVU6Hr1G020750 | 0,76 | 2,43 | unknown |
| HORVU4Hr1G050980 | 0,77 | 4,11 | unknown |
| HORVU1Hr1G060920 | 0,78 | 5,54 | unknown |
| HORVU7Hr1G070780 | 0,80 | 2,12 | unknown |
| HORVU3Hr1G007290 | 0,81 | 3,88 | unknown |
| HORVU2Hr1G014450 | 0,82 | 4,34 | unknown |
| HORVU1Hr1G075670 | 0,82 | 2,20 | unknown |
| HORVU2Hr1G014670 | 0,83 | 3,38 | unknown |
| HORVU3Hr1G095070 | 0,83 | 5,12 | unknown |
| HORVU6Hr1G010890 | 0,84 | 4,18 | unknown |
| HORVU6Hr1G077790 | 0,84 | 7,25 | unknown |
| HORVU2Hr1G112580 | 0,84 | 7,47 | unknown |
| HORVU5Hr1G085510 | 0,85 | 2,08 | unknown |
| HORVU3Hr1G072880 | 0,85 | 7,26 | unknown |
| HORVU3Hr1G088440 | 0,86 | 4,46 | unknown |
| HORVU2Hr1G092360 | 0,87 | 2,52 | unknown |
| HORVU6Hr1G073730 | 0,87 | 2,07 | unknown |
| HORVU1Hr1G069310 | 0,87 | 5,91 | unknown |
| HORVU3Hr1G115860 | 0,88 | 4,09 | unknown |
| HORVU3Hr1G078960 | 0,89 | 5,79 | unknown |
| HORVU4Hr1G008180 | 0,89 | 2,68 | unknown |
| HORVU2Hr1G101150 | 0,90 | 3,72 | unknown |
| HORVU0Hr1G005920 | 0,90 | 2,25 | unknown |
| HORVU2Hr1G072670 | 0,90 | 5,14 | unknown |
| HORVU4Hr1G059950 | 0,91 | 5,34 | unknown |
| HORVU1Hr1G063740 | 0,93 | 2,67 | unknown |
| HORVU5Hr1G047060 | 0,93 | 5,65 | unknown |
| HORVU5Hr1G074450 | 0,94 | 8,61 | unknown |
| HORVU5Hr1G103820 | 0,95 | 7,74 | unknown |
| HORVU5Hr1G097730 | 0,96 | 3,83 | unknown |
| HORVU5Hr1G014500 | 0,97 | 7,42 | unknown |
| HORVU5Hr1G072630 | 0,98 | 7,75 | unknown |
| HORVU4Hr1G054770 | 0,98 | 2,34 | unknown |
| HORVU5Hr1G081450 | 0,98 | 9,09 | unknown |
| HORVU3Hr1G026420 | 0,98 | 2,36 | unknown |
| HORVU4Hr1G067340 | 1,00 | 4,87 | unknown |
| HORVU4Hr1G023390 | 1,00 | 2,11 | unknown |
| HORVU2Hr1G017400 | 1,01 | 2,23 | unknown |
| HORVU3Hr1G089770 | 1,01 | 7,04 | unknown |
| HORVU5Hr1G011400 | 1,01 | 5,00 | unknown |
| HORVU3Hr1G032760 | 1,02 | 2,07 | unknown |
| HORVU7Hr1G084610 | 1,02 | 3,65 | unknown |
| HORVU7Hr1G095220 | 1,02 | 3,00 | unknown |
| HORVU4Hr1G005450 | 1,02 | 3,31 | unknown |
| HORVU3Hr1G060310 | 1,04 | 2,62 | unknown |
| HORVU4Hr1G077420 | 1,05 | 2,21 | unknown |
| HORVU3Hr1G061840 | 1,07 | 2,75 | unknown |
| HORVU1Hr1G077230 | 1,07 | 2,66 | unknown |
| HORVU7Hr1G001020 | 1,08 | 2,85 | unknown |
| HORVU1Hr1G018140 | 1,08 | 2,50 | unknown |
| HORVU4Hr1G053250 | 1,09 | 7,56 | unknown |
| HORVU7Hr1G036780 | 1,09 | 4,45 | unknown |
| HORVU1Hr1G026780 | 1,09 | 4,07 | unknown |
| HORVU3Hr1G020640 | 1,10 | 2,16 | unknown |
| HORVU4Hr1G010080 | 1,10 | 6,24 | unknown |
| HORVU5Hr1G022760 | 1,13 | 2,34 | unknown |
| HORVU7Hr1G101240 | 1,15 | 6,11 | unknown |
| HORVU1Hr1G090250 | 1,15 | 4,12 | unknown |
| HORVU2Hr1G093930 | 1,15 | 4,92 | unknown |
| HORVU4Hr1G084600 | 1,16 | 2,31 | unknown |
| HORVU3Hr1G060950 | 1,16 | 7,14 | unknown |
| HORVU0Hr1G039910 | 1,17 | 3,02 | unknown |
| HORVU7Hr1G095060 | 1,19 | 2,11 | unknown |
| HORVU5Hr1G017820 | 1,19 | 3,94 | unknown |
| HORVU3Hr1G019510 | 1,20 | 3,48 | unknown |
| HORVU1Hr1G051360 | 1,21 | 5,68 | unknown |
| HORVU5Hr1G093460 | 1,22 | 3,53 | unknown |
| HORVU5Hr1G089280 | 1,26 | 2,24 | unknown |
| HORVU4Hr1G021190 | 1,26 | 5,24 | unknown |
| HORVU4Hr1G050520 | 1,26 | 6,64 | unknown |
| HORVU2Hr1G101100 | 1,27 | 3,60 | unknown |
| HORVU4Hr1G005800 | 1,28 | 4,15 | unknown |
| HORVU4Hr1G070970 | 1,29 | 7,68 | unknown |
| HORVU1Hr1G044080 | 1,29 | 6,16 | unknown |
| HORVU5Hr1G119180 | 1,29 | 3,66 | unknown |
| HORVU5Hr1G109400 | 1,30 | 2,88 | unknown |
| HORVU3Hr1G074570 | 1,31 | 2,08 | unknown |
| HORVU5Hr1G000240 | 1,34 | 4,47 | unknown |
| HORVU3Hr1G020230 | 1,35 | 3,62 | unknown |
| HORVU1Hr1G000090 | 1,35 | 3,37 | unknown |
| HORVU0Hr1G017080 | 1,38 | 3,25 | unknown |
| HORVU3Hr1G071320 | 1,39 | 3,81 | unknown |
| HORVU2Hr1G060460 | 1,40 | 2,97 | unknown |
| HORVU4Hr1G050250 | 1,42 | 3,71 | unknown |
| HORVU1Hr1G090930 | 1,46 | 6,71 | unknown |
| HORVU5Hr1G010350 | 1,47 | 4,03 | unknown |
| HORVU4Hr1G022780 | 1,47 | 7,66 | unknown |
| HORVU3Hr1G019320 | 1,47 | 2,52 | unknown |
| HORVU7Hr1G043320 | 1,47 | 4,61 | unknown |
| HORVU7Hr1G052750 | 1,47 | 3,38 | unknown |
| HORVU4Hr1G051360 | 1,48 | 3,74 | unknown |
| HORVU7Hr1G042300 | 1,49 | 7,98 | unknown |
| HORVU2Hr1G118950 | 1,51 | 2,07 | unknown |
| HORVU3Hr1G078900 | 1,51 | 3,42 | unknown |
| HORVU1Hr1G039370 | 1,52 | 2,16 | unknown |
| HORVU0Hr1G004850 | 1,52 | 4,28 | unknown |
| HORVU7Hr1G098490 | 1,52 | 2,59 | unknown |
| HORVU6Hr1G084280 | 1,53 | 8,47 | unknown |
| HORVU4Hr1G072480 | 1,53 | 3,02 | unknown |
| HORVU7Hr1G077430 | 1,54 | 8,26 | unknown |
| HORVU7Hr1G096460 | 1,55 | 3,89 | unknown |
| HORVU4Hr1G088670 | 1,55 | 2,69 | unknown |
| HORVU2Hr1G017450 | 1,56 | 7,24 | unknown |
| HORVU0Hr1G016130 | 1,58 | 2,54 | unknown |
| HORVU5Hr1G095910 | 1,58 | 2,20 | unknown |
| HORVU5Hr1G109880 | 1,60 | 4,82 | unknown |
| HORVU2Hr1G099590 | 1,62 | 7,39 | unknown |
| HORVU3Hr1G020210 | 1,64 | 4,35 | unknown |
| HORVU6Hr1G055910 | 1,65 | 2,48 | unknown |
| HORVU6Hr1G084070 | 1,66 | 7,10 | unknown |
| HORVU5Hr1G075200 | 1,68 | 2,86 | unknown |
| HORVU2Hr1G041080 | 1,69 | 6,07 | unknown |
| HORVU5Hr1G018870 | 1,69 | 4,30 | unknown |
| HORVU5Hr1G042030 | 1,69 | 2,17 | unknown |
| HORVU3Hr1G081580 | 1,70 | 3,42 | unknown |
| HORVU1Hr1G017660 | 1,71 | 6,29 | unknown |
| HORVU2Hr1G099290 | 1,71 | 4,89 | unknown |
| HORVU2Hr1G077990 | 1,72 | 2,37 | unknown |
| HORVU7Hr1G049860 | 1,72 | 4,56 | unknown |
| HORVU3Hr1G069650 | 1,73 | 5,01 | unknown |
| HORVU3Hr1G029260 | 1,74 | 5,58 | unknown |
| HORVU0Hr1G004670 | 1,74 | 6,27 | unknown |
| HORVU7Hr1G074330 | 1,75 | 5,96 | unknown |
| HORVU1Hr1G068910 | 1,76 | 3,81 | unknown |
| HORVU6Hr1G075970 | 1,77 | 3,79 | unknown |
| HORVU4Hr1G067180 | 1,77 | 5,56 | unknown |
| HORVU2Hr1G092800 | 1,77 | 4,98 | unknown |
| HORVU4Hr1G012170 | 1,79 | 4,92 | unknown |
| HORVU3Hr1G069240 | 1,80 | 7,66 | unknown |
| HORVU5Hr1G046050 | 1,82 | 4,38 | unknown |
| HORVU4Hr1G078400 | 1,83 | 3,72 | unknown |
| HORVU1Hr1G076390 | 1,83 | 5,05 | unknown |
| HORVU1Hr1G064640 | 1,84 | 7,07 | unknown |
| HORVU1Hr1G073460 | 1,84 | 3,72 | unknown |
| HORVU6Hr1G016710 | 1,84 | 6,42 | unknown |
| HORVU5Hr1G124380 | 1,85 | 5,45 | unknown |
| HORVU1Hr1G050620 | 1,86 | 2,09 | unknown |
| HORVU1Hr1G014730 | 1,86 | 4,29 | unknown |
| HORVU4Hr1G060740 | 1,87 | 3,42 | unknown |
| HORVU5Hr1G096010 | 1,89 | 2,25 | unknown |
| HORVU7Hr1G116880 | 1,89 | 2,82 | unknown |
| HORVU5Hr1G051370 | 1,90 | 8,59 | unknown |
| HORVU1Hr1G043090 | 1,90 | 6,38 | unknown |
| HORVU3Hr1G096510 | 1,91 | 3,09 | unknown |
| HORVU7Hr1G091100 | 1,92 | 6,31 | unknown |
| HORVU3Hr1G106140 | 1,93 | 7,09 | unknown |
| HORVU3Hr1G068890 | 1,94 | 8,78 | unknown |
| HORVU2Hr1G080480 | 1,94 | 3,59 | unknown |
| HORVU3Hr1G077170 | 1,95 | 2,17 | unknown |
| HORVU3Hr1G084830 | 1,95 | 4,92 | unknown |
| HORVU1Hr1G075900 | 1,96 | 3,41 | unknown |
| HORVU7Hr1G096310 | 1,97 | 5,09 | unknown |
| HORVU7Hr1G035190 | 1,98 | 4,73 | unknown |
| HORVU3Hr1G007540 | 1,99 | 4,18 | unknown |
| HORVU7Hr1G106280 | 2,00 | 3,50 | unknown |
| HORVU3Hr1G083560 | -0,18 | 2,18 | urate oxidase [EC:1.7.3.3] |
| HORVU6Hr1G006880 | 1,18 | 4,44 | vitellogenic carboxypeptidase-like protein [EC:3.4.16.-] |
| HORVU3Hr1G066240 | -0,65 | 3,62 | voltage-gated clc-type chloride channel eric |
| HORVU3Hr1G022900 | -1,96 | 3,37 | wd40 repeat protein |
| HORVU6Hr1G034250 | 0,19 | 8,02 | wdsam1 protein |
| HORVU1Hr1G074180 | 1,99 | 3,02 | wdsam1 protein |
| HORVU2Hr1G037990 | -0,66 | 2,20 | wnk lysine deficient protein kinase [EC:2.7.11.1] |
| HORVU7Hr1G070010 | -1,21 | 3,45 | x-box transcription factor-related |
| HORVU7Hr1G005270 | 0,93 | 5,68 | x-box transcription factor-related |
| HORVU5Hr1G071140 | 1,86 | 4,76 | x-box transcription factor-related |
| HORVU4Hr1G005530 | 1,65 | 4,55 | xanthine-uracil / vitamin C permease family member |
| HORVU5Hr1G055560 | 1,74 | 2,46 | xanthine-uracil / vitamin C permease family member |
| HORVU2Hr1G108420 | -1,43 | 7,24 | xyloglucan:xyloglucosyl transferase [EC:2.4.1.207] |
| HORVU4Hr1G090820 | -0,07 | 9,35 | xyloglucan:xyloglucosyl transferase [EC:2.4.1.207] |
| HORVU6Hr1G093230 | 0,80 | 7,03 | xyloglucan:xyloglucosyl transferase [EC:2.4.1.207] |
| HORVU2Hr1G034630 | 1,98 | 3,06 | zinc finger dhhc domain containing protein |
| HORVU7Hr1G041690 | -0,25 | 9,95 | zinc finger five domain containing protein |
| HORVU3Hr1G071400 | -0,18 | 4,56 | zinc finger five domain containing protein |
| HORVU2Hr1G025800 | 0,46 | 10,12 | zinc finger five domain containing protein |
| HORVU1Hr1G014950 | 0,94 | 2,02 | zinc finger protein with krab and scan domains |
| HORVU1Hr1G024390 | 0,75 | 3,04 | zinc/iron transporter |
