# Supplementary material for: Differential Expression Profiling of Microspores During the Early Stages of Isolated Microspore Culture Using the Responsive Barley Cultivar Gobernadora
Source: G3 (Bethesda). 2018 Mar 12;8(5):1603–14. doi: 10.1534/g3.118.200208 (PMC5940152; doi:10.1534/g3.118.200208)
Supplement: Supplementary file 7 [file 1603TableS7.docx]

Supplementary Table 7: Gene functional annotation for genes in cluster 7

| Gene stable ID (cluster 7) | logFC D2-D0 | logFC D5-D2 | Gene function annotation |
| --- | --- | --- | --- |
| HORVU4Hr1G048470 | 2,49 | -3,77 | 1-phosphatidylinositol-4-phosphate 5-kinase [EC:2.7.1.68] |
| HORVU4Hr1G046320 | 2,77 | -3,64 | 1-phosphatidylinositol-4-phosphate 5-kinase [EC:2.7.1.68] |
| HORVU5Hr1G042990 | 7,67 | -6,09 | 1-phosphatidylinositol-4-phosphate 5-kinase [EC:2.7.1.68] |
| HORVU2Hr1G072500 | 2,10 | -2,22 | 1,4-alpha-glucan branching enzyme [EC:2.4.1.18] |
| HORVU2Hr1G021700 | 2,05 | -3,47 | 4-alpha-glucanotransferase [EC:2.4.1.25] |
| HORVU3Hr1G070300 | 3,45 | -2,02 | 6-phosphofructokinase [EC:2.7.1.11] |
| HORVU5Hr1G000180 | 5,20 | -3,57 | actin |
| HORVU5Hr1G047480 | 2,62 | -2,64 | adenylyl-sulfate reductase (glutathione) [EC:1.8.4.9] |
| HORVU5Hr1G024040 | 3,08 | -2,36 | alcohol dehydrogenase related |
| HORVU4Hr1G016780 | 4,78 | -2,55 | alcohol dehydrogenase related |
| HORVU3Hr1G098340 | 4,18 | -2,11 | alpha/beta hydrolase fold-containing protein |
| HORVU3Hr1G094560 | 6,63 | -4,32 | AMP-activated protein kinase, gama regulatory subunit |
| HORVU1Hr1G025890 | 3,19 | -3,59 | anion exchange protein |
| HORVU7Hr1G081770 | 2,83 | -2,65 | aquaporin TIP |
| HORVU3Hr1G116790 | 5,42 | -3,95 | aquaporin TIP |
| HORVU7Hr1G055220 | 2,66 | -2,12 | arf-GAP with coiled-coil, ANK repeat and PH domain-containing protein |
| HORVU6Hr1G021620 | 5,38 | -2,64 | arsenite-resistance protein 2 |
| HORVU2Hr1G066860 | 3,43 | -2,53 | aspartyl protease |
| HORVU3Hr1G056630 | 6,86 | -4,63 | aspartyl protease; phytepsin [EC:3.4.23.40] |
| HORVU5Hr1G049450 | 4,99 | -3,58 | ATP-binding cassette transporter |
| HORVU5Hr1G049470 | 5,00 | -3,32 | ATP-binding cassette transporter |
| HORVU1Hr1G073490 | 2,37 | -2,48 | auxin influx carrier (AUX1 LAX family) |
| HORVU7Hr1G061800 | 4,99 | -3,55 | beta catenin-related armadillo repeat-containing |
| HORVU4Hr1G084390 | 5,21 | -3,51 | beta-amylase [EC:3.2.1.2] |
| HORVU1Hr1G038920 | 7,54 | -3,06 | beta-amylase [EC:3.2.1.2] |
| HORVU7Hr1G057460 | 4,16 | -3,28 | beta-galactosidase related |
| HORVU7Hr1G009630 | 5,02 | -4,25 | beta-galactosidase related |
| HORVU7Hr1G091730 | 5,90 | -4,11 | beta-glucosidase [EC:3.2.1.21] |
| HORVU4Hr1G069990 | 2,35 | -2,52 | Ca2+-transporting ATPase [EC:3.6.3.8] |
| HORVU1Hr1G080790 | 4,19 | -2,89 | calcineurin B |
| HORVU3Hr1G077070 | 4,73 | -2,11 | calcium binding protein |
| HORVU7Hr1G008910 | 7,77 | -6,23 | calcium-binding protein CML |
| HORVU7Hr1G056060 | 2,09 | -2,61 | calcium-dependent protein kinase [EC:2.7.11.1] |
| HORVU5Hr1G029260 | 5,50 | -4,38 | calcium-dependent protein kinase [EC:2.7.11.1] |
| HORVU1Hr1G061970 | 3,45 | -3,37 | carboxymethylenebutenolidase [EC:3.1.1.45] |
| HORVU6Hr1G093390 | 2,31 | -4,40 | cell cycle control protein 50 |
| HORVU6Hr1G091350 | 3,44 | -2,07 | centaurin/ARF |
| HORVU2Hr1G063790 | 4,76 | -6,25 | centaurin/ARF |
| HORVU2Hr1G034610 | 4,07 | -3,76 | CGI-141-related/lipase containing protein |
| HORVU2Hr1G012100 | 9,10 | -5,55 | clatherin light chain |
| HORVU2Hr1G097130 | 2,92 | -3,23 | cofilin |
| HORVU6Hr1G063480 | 8,37 | -4,09 | cysteine synthase A [EC:2.5.1.47] |
| HORVU3Hr1G092270 | 6,47 | -4,10 | cysteine-rich repeat secretory protein |
| HORVU7Hr1G008210 | 3,25 | -4,00 | disease resistance protein RPM1 |
| HORVU4Hr1G020790 | 4,99 | -2,63 | disulfide oxidoreductase |
| HORVU0Hr1G000620 | 2,29 | -3,38 | DnaJ homolog subfamily B member 13 |
| HORVU3Hr1G113710 | 5,16 | -6,98 | DnaJ homolog subfamily B member 4 |
| HORVU5Hr1G123360 | 2,41 | -2,22 | E3 ubiquitin-protein ligase UHRF-related |
| HORVU2Hr1G122390 | 3,30 | -4,68 | EH domain-containing protein 1 |
| HORVU7Hr1G091570 | 2,32 | -2,04 | endo-1,4-beta-glucanase |
| HORVU2Hr1G120100 | 2,34 | -2,85 | endo-1,4-beta-glucanase |
| HORVU2Hr1G006770 | 4,44 | -3,81 | engulfment and cell motility |
| HORVU7Hr1G017640 | 6,18 | -2,71 | enolase [EC:4.2.1.11] |
| HORVU1Hr1G010840 | 6,17 | -4,72 | EPSIN/ENT-related |
| HORVU2Hr1G118510 | 3,05 | -2,87 | exocyst complexe protein EXO70 |
| HORVU5Hr1G039240 | 3,73 | -3,08 | exocyst complexe protein EXO70 |
| HORVU2Hr1G071380 | 3,86 | -2,46 | exotosin(heparan sulfate glycosyltransferase)-related |
| HORVU2Hr1G034860 | 6,78 | -4,82 | extensin, proline-rich protein |
| HORVU1Hr1G090940 | 2,27 | -2,79 | extracellular signal-regulated kinase 1/2  [EC:2.7.11.24] |
| HORVU5Hr1G093830 | 7,57 | -4,66 | F-BOX AND WD40 DOMAIN PROTEIN |
| HORVU5Hr1G004830 | 2,94 | -3,22 | F-type H+-transporting ATPase subunit epsilon [EC:3.6.3.14] |
| HORVU6Hr1G052360 | 4,35 | -2,51 | fimbrin/plastin |
| HORVU3Hr1G042970 | 5,55 | -5,39 | galacturan 1,4-alpha-galacturonidase [EC:3.2.1.67] |
| HORVU7Hr1G094750 | 7,56 | -6,94 | galacturan 1,4-alpha-galacturonidase [EC:3.2.1.67] |
| HORVU2Hr1G012340 | 6,49 | -2,52 | glucosyl/glucuronosyl transferase |
| HORVU2Hr1G078560 | 6,89 | -3,50 | glutamate decarboxylase [EC:4.1.1.15] |
| HORVU1Hr1G060210 | 4,87 | -4,18 | glutaredoxin |
| HORVU7Hr1G002370 | 7,17 | -3,74 | glutathione S-transferase [EC:2.5.1.18] |
| HORVU1Hr1G001560 | 8,95 | -3,24 | glutathione S-transferase [EC:2.5.1.18] |
| HORVU1Hr1G080970 | 3,87 | -2,91 | glycerophosphoryl diester phorphodiesterase |
| HORVU7Hr1G080190 | 3,18 | -2,58 | GTP-binding protein SAR1 [EC:3.6.5.-] |
| HORVU0Hr1G017350 | 3,69 | -2,74 | H+-transporting ATPase [EC:3.6.3.6] |
| HORVU5Hr1G078400 | 3,69 | -4,84 | heat shock 70kDa protein 5 |
| HORVU7Hr1G098810 | 3,70 | -5,38 | heat shock 70kDa protein 5 |
| HORVU2Hr1G122760 | 4,13 | -4,92 | heat shock 70kDa protein 5 |
| HORVU7Hr1G107190 | 4,95 | -2,38 | heat shock 70kDa protein 5 |
| HORVU5Hr1G083940 | 5,07 | -3,83 | homeobox-leucine zipper protein |
| HORVU5Hr1G067500 | 7,32 | -5,17 | HVA22-like proteins |
| HORVU6Hr1G070060 | 2,32 | -2,68 | hydroxymethylglutaryl-CoA reductase (NADPH) [EC:1.1.1.34] |
| HORVU5Hr1G052150 | 3,65 | -3,80 | indole-3-glycerol phosphate synthase [EC:4.1.1.48]; tryptophan biosynthesis protein |
| HORVU1Hr1G075660 | 4,69 | -3,85 | inorganic pyrophosphatase [EC:3.6.1.1] |
| HORVU5Hr1G075330 | 2,54 | -2,42 | inositol 5-phosphate |
| HORVU5Hr1G024150 | 2,35 | -3,21 | ISP4 like protein |
| HORVU2Hr1G012830 | 8,56 | -3,29 | L-lactate dehydrogenase [EC:1.1.1.27] |
| HORVU4Hr1G061980 | 2,53 | -2,66 | large subunit ribosomal protein L40e; ubiquitin |
| HORVU6Hr1G053210 | 2,28 | -4,56 | leucine-rich repeat-containing protein |
| HORVU1Hr1G083100 | 2,34 | -2,17 | leucine-rich repeat-containing protein |
| HORVU7Hr1G120540 | 2,55 | -2,91 | leucine-rich repeat-containing protein |
| HORVU7Hr1G023820 | 2,59 | -2,33 | leucine-rich repeat-containing protein |
| HORVU4Hr1G005500 | 2,70 | -2,65 | lysyl-tRNA synthetase, class II [EC:6.1.1.6] |
| HORVU4Hr1G003610 | 3,32 | -4,42 | MADS BOX protein |
| HORVU0Hr1G004410 | 8,43 | -5,58 | member of 'GDXG' family of lipolytic enzyme |
| HORVU3Hr1G052360 | 3,80 | -3,11 | membrane protein CH1 related |
| HORVU4Hr1G055610 | 2,63 | -2,35 | mitochondrial import inner membrane translocase subunit |
| HORVU6Hr1G088830 | 4,24 | -4,40 | MKIAA1688 protein |
| HORVU3Hr1G090450 | 5,28 | -2,14 | mlo protein |
| HORVU1Hr1G069130 | 7,99 | -3,73 | multi-copper oxidase |
| HORVU0Hr1G022350 | 8,09 | -2,38 | multidrug resistance protein, MATE family |
| HORVU1Hr1G017970 | 2,33 | -2,20 | myb proto-oncogene protein, plant |
| HORVU7Hr1G105520 | 4,22 | -4,94 | myb proto-oncogene protein, plant |
| HORVU5Hr1G036900 | 4,30 | -4,11 | myb proto-oncogene protein, plant |
| HORVU6Hr1G026450 | 2,44 | -3,28 | MYB-like DNA-binding protein MYB |
| HORVU5Hr1G017060 | 7,19 | -4,74 | myosin |
| HORVU7Hr1G038500 | 3,54 | -3,70 | myosin V |
| HORVU1Hr1G071340 | 4,14 | -2,64 | NADPH oxidase |
| HORVU1Hr1G042910 | 2,47 | -2,35 | osmotic stress potassium transporter |
| HORVU1Hr1G066230 | 6,11 | -4,17 | osmotic stress potassium transporter; KUP system potassium uptake protein |
| HORVU6Hr1G078640 | 8,20 | -3,42 | oxidoreductase, 2OG-FE(II) oxygenase family protein |
| HORVU7Hr1G019500 | 7,43 | -4,33 | pectate lyase [EC:4.2.2.2] |
| HORVU7Hr1G085860 | 8,30 | -5,08 | pectate lyase [EC:4.2.2.2] |
| HORVU5Hr1G022590 | 2,45 | -3,73 | pectinesterase [EC:3.1.1.11] |
| HORVU7Hr1G018530 | 6,87 | -5,22 | peroxidase [EC:1.11.1.7] |
| HORVU4Hr1G072190 | 8,51 | -4,34 | peroxidase [EC:1.11.1.7] |
| HORVU6Hr1G076470 | 8,70 | -4,16 | peroxidase [EC:1.11.1.7] |
| HORVU7Hr1G048090 | 8,90 | -4,49 | peroxidase [EC:1.11.1.7] |
| HORVU5Hr1G021130 | 3,02 | -2,50 | phosphatidylinositol N-acetylglucosaminytransferase subunit P (down syndrome critical region protein 5)-related |
| HORVU3Hr1G000940 | 7,53 | -5,49 | phosphoenolpyruvate carboxylase [EC:4.1.1.31] |
| HORVU1Hr1G014570 | 3,09 | -3,13 | phospholipase C, delta [EC:3.1.4.11] |
| HORVU4Hr1G085550 | 3,92 | -3,90 | phospholipase D [EC:3.1.4.4] |
| HORVU1Hr1G025370 | 5,23 | -3,06 | phospholipase D [EC:3.1.4.4] |
| HORVU5Hr1G049360 | 3,64 | -4,47 | phospholipase-related |
| HORVU1Hr1G075570 | 2,39 | -3,69 | profilin |
| HORVU4Hr1G067080 | 6,99 | -3,48 | programmed cell death 4 |
| HORVU5Hr1G007190 | 4,68 | -3,71 | proprotein convertase subtilisin/kexin |
| HORVU2Hr1G096710 | 8,18 | -6,86 | proprotein convertase subtilisin/kexin |
| HORVU2Hr1G048520 | 2,56 | -2,17 | protein neuralized; inhibitor of apoptosis |
| HORVU2Hr1G086370 | 3,01 | -3,38 | protein phosphatase 2C |
| HORVU4Hr1G015370 | 3,53 | -3,07 | protein phosphatase 2C |
| HORVU4Hr1G053970 | 2,50 | -2,05 | protein phosphatase 2C homolog 2/3 [EC:3.1.3.16] |
| HORVU6Hr1G014720 | 2,60 | -5,34 | protein regulator of cytokinesis 1 PRC1-related |
| HORVU4Hr1G059240 | 3,39 | -3,55 | RAB GDP-dissociation inhibitor |
| HORVU1Hr1G029920 | 2,36 | -3,77 | RAG1-activating protein 1 |
| HORVU4Hr1G002310 | 5,06 | -2,20 | RAG1-activating protein 1 |
| HORVU6Hr1G012690 | 2,71 | -2,02 | Ras-related C3 botulinum toxin substrate 1 |
| HORVU1Hr1G078890 | 2,48 | -3,01 | regulator of chromosome condensation |
| HORVU1Hr1G017470 | 4,99 | -3,86 | reticulon |
| HORVU1Hr1G031420 | 9,46 | -5,17 | ribosomal protein S6 kinase |
| HORVU6Hr1G040130 | 2,68 | -2,22 | ser/thr dehydratase, trp synthase |
| HORVU4Hr1G026300 | 2,20 | -2,38 | serine/threonine kinase |
| HORVU7Hr1G025390 | 2,95 | -2,10 | starch synthase [EC:2.4.1.21] |
| HORVU2Hr1G030870 | 2,59 | -2,09 | sucrose synthase [EC:2.4.1.13] |
| HORVU1Hr1G035760 | 6,63 | -5,33 | sugar transporter |
| HORVU3Hr1G038320 | 6,44 | -4,86 | symplekin-related |
| HORVU2Hr1G045440 | 8,11 | -3,28 | TBC1 domain family member GTPase-activating protein |
| HORVU2Hr1G097910 | 2,22 | -2,62 | threonine aldolase [EC:4.1.2.5] |
| HORVU5Hr1G050890 | 5,26 | -2,80 | TPR repeat containing protein |
| HORVU7Hr1G001280 | 6,86 | -4,16 | TPR repeat containing protein |
| HORVU5Hr1G094150 | 2,61 | -3,87 | two-component sensor histidine kinase |
| HORVU3Hr1G064710 | 5,84 | -5,05 | uncharacterized ring zinc finger-containing protein |
| HORVU6Hr1G081590 | 2,08 | -3,98 | unknown |
| HORVU3Hr1G032660 | 2,09 | -3,19 | unknown |
| HORVU3Hr1G055900 | 2,14 | -3,48 | unknown |
| HORVU3Hr1G093530 | 2,16 | -2,80 | unknown |
| HORVU6Hr1G022140 | 2,21 | -3,45 | unknown |
| HORVU1Hr1G073080 | 2,21 | -2,52 | unknown |
| HORVU2Hr1G079790 | 2,21 | -2,42 | unknown |
| HORVU7Hr1G045290 | 2,28 | -3,01 | unknown |
| HORVU4Hr1G041430 | 2,31 | -3,00 | unknown |
| HORVU7Hr1G102910 | 2,32 | -2,04 | unknown |
| HORVU3Hr1G075840 | 2,34 | -3,24 | unknown |
| HORVU2Hr1G120440 | 2,38 | -3,79 | unknown |
| HORVU6Hr1G025020 | 2,39 | -2,87 | unknown |
| HORVU6Hr1G030170 | 2,40 | -2,07 | unknown |
| HORVU2Hr1G117800 | 2,44 | -2,99 | unknown |
| HORVU0Hr1G012850 | 2,48 | -3,93 | unknown |
| HORVU0Hr1G021920 | 2,53 | -5,09 | unknown |
| HORVU3Hr1G076740 | 2,57 | -3,03 | unknown |
| HORVU3Hr1G111230 | 2,71 | -2,27 | unknown |
| HORVU1Hr1G073150 | 2,82 | -3,03 | unknown |
| HORVU2Hr1G109810 | 2,84 | -2,71 | unknown |
| HORVU1Hr1G084720 | 2,85 | -2,39 | unknown |
| HORVU5Hr1G005190 | 2,89 | -2,17 | unknown |
| HORVU6Hr1G053490 | 2,91 | -2,01 | unknown |
| HORVU5Hr1G058980 | 2,93 | -3,59 | unknown |
| HORVU1Hr1G086590 | 2,97 | -2,35 | unknown |
| HORVU2Hr1G099200 | 2,99 | -3,96 | unknown |
| HORVU7Hr1G046210 | 3,01 | -3,99 | unknown |
| HORVU1Hr1G071280 | 3,05 | -3,79 | unknown |
| HORVU2Hr1G084420 | 3,07 | -5,66 | unknown |
| HORVU3Hr1G086000 | 3,08 | -3,57 | unknown |
| HORVU2Hr1G030260 | 3,08 | -4,02 | unknown |
| HORVU0Hr1G015590 | 3,10 | -3,07 | unknown |
| HORVU2Hr1G008230 | 3,10 | -2,87 | unknown |
| HORVU6Hr1G006150 | 3,11 | -2,71 | unknown |
| HORVU2Hr1G097210 | 3,16 | -2,07 | unknown |
| HORVU4Hr1G034650 | 3,19 | -2,28 | unknown |
| HORVU2Hr1G081900 | 3,33 | -2,59 | unknown |
| HORVU4Hr1G083510 | 3,36 | -3,32 | unknown |
| HORVU4Hr1G058450 | 3,37 | -3,34 | unknown |
| HORVU4Hr1G050910 | 3,38 | -3,18 | unknown |
| HORVU0Hr1G003930 | 3,43 | -2,21 | unknown |
| HORVU2Hr1G032230 | 3,49 | -2,16 | unknown |
| HORVU4Hr1G015710 | 3,56 | -2,26 | unknown |
| HORVU6Hr1G067270 | 3,58 | -3,68 | unknown |
| HORVU3Hr1G012310 | 3,59 | -5,23 | unknown |
| HORVU4Hr1G002700 | 3,62 | -3,28 | unknown |
| HORVU7Hr1G106910 | 3,65 | -3,59 | unknown |
| HORVU1Hr1G052400 | 3,67 | -2,38 | unknown |
| HORVU2Hr1G097450 | 3,68 | -2,28 | unknown |
| HORVU7Hr1G089210 | 3,70 | -2,03 | unknown |
| HORVU6Hr1G035270 | 3,78 | -3,56 | unknown |
| HORVU1Hr1G070400 | 3,79 | -3,48 | unknown |
| HORVU2Hr1G119430 | 3,88 | -4,78 | unknown |
| HORVU3Hr1G052940 | 3,92 | -2,00 | unknown |
| HORVU6Hr1G063760 | 3,95 | -3,95 | unknown |
| HORVU4Hr1G083660 | 3,99 | -2,76 | unknown |
| HORVU1Hr1G050600 | 3,99 | -3,69 | unknown |
| HORVU7Hr1G078360 | 4,06 | -4,88 | unknown |
| HORVU1Hr1G020550 | 4,10 | -4,09 | unknown |
| HORVU3Hr1G058700 | 4,18 | -3,11 | unknown |
| HORVU1Hr1G073780 | 4,20 | -5,06 | unknown |
| HORVU4Hr1G008080 | 4,24 | -2,58 | unknown |
| HORVU4Hr1G020000 | 4,31 | -2,48 | unknown |
| HORVU4Hr1G022080 | 4,38 | -2,29 | unknown |
| HORVU6Hr1G076060 | 4,46 | -3,51 | unknown |
| HORVU7Hr1G108350 | 4,51 | -4,38 | unknown |
| HORVU0Hr1G019570 | 4,52 | -3,63 | unknown |
| HORVU7Hr1G036920 | 4,55 | -2,40 | unknown |
| HORVU4Hr1G012680 | 4,59 | -2,43 | unknown |
| HORVU1Hr1G090230 | 4,70 | -4,17 | unknown |
| HORVU5Hr1G124590 | 4,72 | -3,31 | unknown |
| HORVU5Hr1G056740 | 4,80 | -2,73 | unknown |
| HORVU3Hr1G057860 | 4,83 | -3,23 | unknown |
| HORVU5Hr1G008960 | 4,91 | -3,48 | unknown |
| HORVU5Hr1G107240 | 4,98 | -3,72 | unknown |
| HORVU7Hr1G116800 | 5,03 | -5,37 | unknown |
| HORVU3Hr1G085660 | 5,13 | -6,76 | unknown |
| HORVU7Hr1G058090 | 5,19 | -2,87 | unknown |
| HORVU2Hr1G122890 | 5,30 | -3,60 | unknown |
| HORVU1Hr1G091020 | 5,36 | -3,96 | unknown |
| HORVU4Hr1G068490 | 5,38 | -3,86 | unknown |
| HORVU2Hr1G049250 | 5,48 | -3,96 | unknown |
| HORVU3Hr1G034740 | 5,48 | -2,52 | unknown |
| HORVU6Hr1G000270 | 5,51 | -4,03 | unknown |
| HORVU5Hr1G092620 | 5,60 | -2,24 | unknown |
| HORVU5Hr1G054150 | 5,61 | -7,06 | unknown |
| HORVU7Hr1G074490 | 5,70 | -2,92 | unknown |
| HORVU5Hr1G111650 | 5,96 | -3,41 | unknown |
| HORVU2Hr1G113150 | 5,98 | -3,14 | unknown |
| HORVU3Hr1G053440 | 6,02 | -5,31 | unknown |
| HORVU3Hr1G089290 | 6,04 | -3,61 | unknown |
| HORVU1Hr1G038740 | 6,07 | -5,89 | unknown |
| HORVU4Hr1G088830 | 6,16 | -3,66 | unknown |
| HORVU6Hr1G050860 | 6,37 | -5,98 | unknown |
| HORVU5Hr1G010240 | 6,37 | -4,56 | unknown |
| HORVU6Hr1G014820 | 6,46 | -4,31 | unknown |
| HORVU1Hr1G027920 | 6,60 | -3,89 | unknown |
| HORVU3Hr1G061560 | 6,75 | -2,17 | unknown |
| HORVU1Hr1G017840 | 6,80 | -4,27 | unknown |
| HORVU5Hr1G115690 | 6,82 | -2,78 | unknown |
| HORVU6Hr1G072990 | 6,85 | -3,19 | unknown |
| HORVU5Hr1G071860 | 6,88 | -3,44 | unknown |
| HORVU5Hr1G071190 | 6,90 | -3,45 | unknown |
| HORVU1Hr1G035080 | 6,92 | -7,02 | unknown |
| HORVU3Hr1G032030 | 6,95 | -3,87 | unknown |
| HORVU3Hr1G050800 | 6,97 | -4,66 | unknown |
| HORVU5Hr1G125140 | 7,03 | -4,46 | unknown |
| HORVU1Hr1G031780 | 7,05 | -5,82 | unknown |
| HORVU7Hr1G069030 | 7,16 | -4,29 | unknown |
| HORVU5Hr1G024380 | 7,20 | -5,00 | unknown |
| HORVU4Hr1G000560 | 7,23 | -5,22 | unknown |
| HORVU4Hr1G064760 | 7,23 | -6,14 | unknown |
| HORVU3Hr1G066000 | 7,26 | -3,87 | unknown |
| HORVU7Hr1G092010 | 7,32 | -4,62 | unknown |
| HORVU5Hr1G014860 | 7,36 | -4,49 | unknown |
| HORVU5Hr1G115910 | 7,37 | -4,92 | unknown |
| HORVU5Hr1G116630 | 7,45 | -5,10 | unknown |
| HORVU3Hr1G026830 | 7,47 | -3,73 | unknown |
| HORVU6Hr1G063220 | 7,66 | -2,94 | unknown |
| HORVU6Hr1G060580 | 7,71 | -5,25 | unknown |
| HORVU3Hr1G033620 | 7,74 | -2,64 | unknown |
| HORVU1Hr1G088890 | 7,77 | -5,01 | unknown |
| HORVU5Hr1G025100 | 7,80 | -7,21 | unknown |
| HORVU6Hr1G016420 | 7,83 | -4,32 | unknown |
| HORVU0Hr1G022130 | 7,86 | -4,47 | unknown |
| HORVU5Hr1G121550 | 7,89 | -6,17 | unknown |
| HORVU6Hr1G094450 | 7,95 | -6,39 | unknown |
| HORVU6Hr1G094420 | 8,01 | -5,12 | unknown |
| HORVU6Hr1G025520 | 8,06 | -6,28 | unknown |
| HORVU3Hr1G116310 | 8,11 | -2,89 | unknown |
| HORVU2Hr1G065390 | 8,11 | -4,46 | unknown |
| HORVU6Hr1G014990 | 8,12 | -4,57 | unknown |
| HORVU5Hr1G107180 | 8,15 | -6,26 | unknown |
| HORVU2Hr1G106510 | 8,20 | -5,77 | unknown |
| HORVU3Hr1G040880 | 8,23 | -4,97 | unknown |
| HORVU5Hr1G060490 | 8,24 | -6,57 | unknown |
| HORVU2Hr1G105300 | 8,26 | -3,30 | unknown |
| HORVU4Hr1G086080 | 8,26 | -7,89 | unknown |
| HORVU5Hr1G031550 | 8,33 | -4,80 | unknown |
| HORVU5Hr1G059160 | 8,37 | -2,15 | unknown |
| HORVU3Hr1G095900 | 8,39 | -6,92 | unknown |
| HORVU3Hr1G115650 | 8,41 | -5,87 | unknown |
| HORVU3Hr1G087910 | 8,43 | -6,46 | unknown |
| HORVU5Hr1G115920 | 8,44 | -5,67 | unknown |
| HORVU6Hr1G015110 | 8,47 | -4,83 | unknown |
| HORVU0Hr1G028890 | 8,49 | -6,69 | unknown |
| HORVU2Hr1G007680 | 8,49 | -4,24 | unknown |
| HORVU5Hr1G025090 | 8,50 | -7,19 | unknown |
| HORVU0Hr1G007070 | 8,50 | -6,77 | unknown |
| HORVU1Hr1G002420 | 8,51 | -4,00 | unknown |
| HORVU3Hr1G095800 | 8,53 | -8,10 | unknown |
| HORVU5Hr1G024830 | 8,56 | -6,56 | unknown |
| HORVU1Hr1G093320 | 8,61 | -7,91 | unknown |
| HORVU6Hr1G010460 | 8,62 | -6,28 | unknown |
| HORVU4Hr1G087070 | 8,68 | -6,07 | unknown |
| HORVU0Hr1G038200 | 8,71 | -7,21 | unknown |
| HORVU0Hr1G007540 | 8,73 | -7,95 | unknown |
| HORVU5Hr1G000420 | 8,74 | -6,17 | unknown |
| HORVU2Hr1G080170 | 8,78 | -6,57 | unknown |
| HORVU4Hr1G086830 | 8,80 | -5,51 | unknown |
| HORVU5Hr1G024820 | 8,91 | -6,40 | unknown |
| HORVU5Hr1G025000 | 8,96 | -7,25 | unknown |
| HORVU3Hr1G012630 | 8,97 | -7,13 | unknown |
| HORVU4Hr1G086890 | 9,08 | -3,53 | unknown |
| HORVU1Hr1G027540 | 9,18 | -5,77 | unknown |
| HORVU6Hr1G005560 | 9,25 | -5,96 | unknown |
| HORVU5Hr1G085100 | 9,31 | -3,98 | unknown |
| HORVU7Hr1G094730 | 9,34 | -3,83 | unknown |
| HORVU0Hr1G026550 | 9,58 | -6,82 | unknown |
| HORVU5Hr1G061930 | 9,92 | -2,65 | unknown |
| HORVU5Hr1G025170 | 9,99 | -7,31 | unknown |
| HORVU0Hr1G032440 | 10,85 | -6,88 | unknown |
| HORVU1Hr1G069480 | 3,12 | -3,91 | V-type H+-transporting ATPase subunit E [EC:3.6.3.14] |
| HORVU3Hr1G117280 | 4,67 | -4,24 | vesicle protein sorting-associated |
| HORVU6Hr1G062050 | 3,66 | -3,81 | vesicle-associated membrane protein-associated protein |
| HORVU4Hr1G049770 | 2,02 | -2,33 | villin |
| HORVU2Hr1G083820 | 6,57 | -3,97 | voltage and ligand gated potassium channel |
| HORVU4Hr1G018960 | 3,25 | -3,92 | voltage and ligand gated potassium channel; cyclic nucleotide gated channel, other eukaryote |
| HORVU7Hr1G026180 | 3,11 | -2,46 | xyloglucan fucosyltransferase [EC:2.4.1.-] |
| HORVU7Hr1G076290 | 4,08 | -2,98 | zinc finger five domain containing protein |
| HORVU6Hr1G018750 | 6,59 | -2,55 | zinc finger five domain containing protein |
| HORVU2Hr1G068430 | 7,14 | -3,20 | zinc finger five domain containing protein |
