# Supplementary material for: Differential Expression Profiling of Microspores During the Early Stages of Isolated Microspore Culture Using the Responsive Barley Cultivar Gobernadora
Source: G3 (Bethesda). 2018 Mar 12;8(5):1603–14. doi: 10.1534/g3.118.200208 (PMC5940152; doi:10.1534/g3.118.200208)
Supplement: Supplementary file 8 [file 1603TableS8.docx]

Supplementary Table 8: Gene functional annotation for genes in cluster 8

| Gene stable ID (cluster 8) | logFC D2-D0 | logFC D5-D2 | Gene function annotation |
| --- | --- | --- | --- |
| HORVU3Hr1G116200 | -2,00 | 2,74 | 3-hydroxy acyl-CoA dehydratase [EC:4.2.1.-] |
| HORVU4Hr1G058070 | -4,78 | 4,31 | ATP-binding cassette transporter |
| HORVU7Hr1G092990 | -4,86 | 3,86 | beta-mannan synthase [EC:2.4.1.32] |
| HORVU1Hr1G049860 | -2,85 | 4,23 | BR-signaling kinase [EC:2.7.11.1] |
| HORVU4Hr1G023520 | -3,05 | 2,01 | CDC45-related protein |
| HORVU3Hr1G030890 | -3,42 | 3,69 | chlorophyll(ide) b reductase [EC:1.1.1.294] |
| HORVU5Hr1G060480 | -3,06 | 2,52 | chromosome transmission fidelity protein 4; WD repeat and HMG-BOX DNA binding domain |
| HORVU1Hr1G062110 | -2,04 | 3,45 | dienelactone hydrolase |
| HORVU1Hr1G059180 | -2,39 | 2,21 | DNA primase small subunit [EC:2.7.7.-] |
| HORVU7Hr1G115770 | -2,51 | 3,91 | DNA-directed RNA polymerase |
| HORVU3Hr1G020860 | -2,48 | 2,05 | DNA-directed RNA polymerase I subunit RPA43; histone-like transcription factor CCAAT-related |
| HORVU1Hr1G000520 | -2,16 | 2,40 | E3 ubiquitin-protein ligase UHRF1  [EC:6.3.2.19] |
| HORVU1Hr1G047220 | -2,19 | 8,05 | ferulate-5-hydroxylase  [EC:1.14.-.-] |
| HORVU3Hr1G063050 | -3,45 | 3,52 | glutamate synthase (NADPH/NADH) [EC:1.4.1.13 1.4.1.14] |
| HORVU4Hr1G066860 | -5,44 | 2,85 | glutamine synthetase [EC:6.3.1.2] |
| HORVU1Hr1G021100 | -2,81 | 6,15 | glutaredoxin |
| HORVU3Hr1G084990 | -6,11 | 8,82 | glycerol-3-phosphate acyltransferase [EC:2.3.1.15] |
| HORVU3Hr1G067110 | -2,04 | 2,40 | glycine dehydrogenase [EC:1.4.4.2] |
| HORVU1Hr1G021840 | -2,16 | 2,14 | GTPase, IMAP family member-related |
| HORVU4Hr1G059210 | -2,07 | 3,08 | HSP70 |
| HORVU1Hr1G049920 | -2,59 | 2,25 | histone H2B |
| HORVU4Hr1G067970 | -2,07 | 2,21 | histone H3 |
| HORVU1Hr1G074340 | -2,03 | 2,32 | histone H3 |
| HORVU1Hr1G020040 | -2,11 | 2,04 | histone H4 |
| HORVU4Hr1G067280 | -2,05 | 4,75 | homocysteine S-methyltransferase [EC:2.1.1.10] |
| HORVU4Hr1G057170 | -2,69 | 2,20 | L-ascorbate peroxidase [EC:1.11.1.11] |
| HORVU5Hr1G117080 | -6,99 | 4,48 | MFS transporter, PHS family, inorganic phosphate transporter |
| HORVU1Hr1G063700 | -2,22 | 2,23 | minichromosome maintenance protein 2; DNA replication licensing factor MCM2 |
| HORVU1Hr1G070110 | -2,01 | 2,11 | minichromosome maintenance protein 3; DNA replication licensing factor MCM3 |
| HORVU5Hr1G028260 | -2,04 | 2,07 | minichromosome maintenance protein 7 (cell division control protein 47); DNA replication licensing factor MCM7 |
| HORVU2Hr1G108840 | -2,60 | 4,87 | MKIAA1688 protein |
| HORVU4Hr1G069720 | -2,08 | 5,76 | N-terminal acetyltransferase |
| HORVU2Hr1G027640 | -2,24 | 5,01 | NOTUM-related |
| HORVU5Hr1G007890 | -2,81 | 11,12 | nuclear transcription factor Y, alpha |
| HORVU1Hr1G055440 | -3,69 | 2,23 | nucleoside-diphosphate kinase [EC:2.7.4.6] |
| HORVU2Hr1G112600 | -2,55 | 2,41 | peptidyl-tRNA hydrolase, PTH2 family [EC:3.1.1.29] |
| HORVU1Hr1G040720 | -4,70 | 3,70 | phytoene dehydrogenase |
| HORVU4Hr1G052840 | -2,44 | 2,69 | poly(A) polymerase [EC:2.7.7.19] |
| HORVU0Hr1G031140 | -3,03 | 2,27 | proliferating cell nuclear antigen |
| HORVU0Hr1G023490 | -5,71 | 8,34 | RBR family (RING FINGER IBR domain-containing) |
| HORVU4Hr1G067940 | -2,30 | 2,29 | replication factor A1 |
| HORVU6Hr1G094080 | -2,94 | 2,13 | replication factor A2 |
| HORVU5Hr1G101680 | -2,88 | 2,60 | reticulon |
| HORVU2Hr1G013270 | -3,28 | 2,59 | RING FINGER domain-containing, polycomp group component |
| HORVU4Hr1G083950 | -2,20 | 4,64 | serine protease family S10 serine carboxypeptidase |
| HORVU5Hr1G111670 | -2,04 | 2,10 | serine-threonine protein kinase |
| HORVU6Hr1G080770 | -3,00 | 3,17 | sterol carrier protein 2 (SCP-2) family protein |
| HORVU5Hr1G079180 | -2,96 | 3,22 | sterol-4alpha-carboxylate 3-dehydrogenase [EC:1.1.1.170] |
| HORVU2Hr1G112080 | -2,18 | 5,51 | sugar transporter |
| HORVU7Hr1G078760 | -2,65 | 2,16 | SWI/SNF-related chromatin binding protein |
| HORVU6Hr1G061720 | -2,21 | 4,49 | SWI/SNF-related matrix-associated actin-dependant regulator of chromosome subfamily |
| HORVU6Hr1G061730 | -2,07 | 4,35 | SWI/SNF-related matrix-associated actin-dependant regulator of chromosome subfamily |
| HORVU4Hr1G003660 | -2,90 | 2,52 | TPR repeat containing protein |
| HORVU7Hr1G072390 | -3,42 | 3,06 | TRAF-interacting protein; zn/ring finger protein 2 |
| HORVU2Hr1G120490 | -2,21 | 2,02 | two-component response regulator ARR-A family |
| HORVU5Hr1G079850 | -8,29 | 2,10 | unknown |
| HORVU2Hr1G094690 | -5,21 | 2,99 | unknown |
| HORVU5Hr1G050610 | -5,15 | 4,91 | unknown |
| HORVU7Hr1G008430 | -4,25 | 3,13 | unknown |
| HORVU6Hr1G090290 | -4,08 | 4,52 | unknown |
| HORVU2Hr1G089130 | -4,03 | 2,55 | unknown |
| HORVU1Hr1G056760 | -3,75 | 10,46 | unknown |
| HORVU5Hr1G112020 | -3,16 | 7,25 | unknown |
| HORVU2Hr1G109440 | -3,10 | 6,78 | unknown |
| HORVU4Hr1G061260 | -3,07 | 2,19 | unknown |
| HORVU7Hr1G008700 | -3,06 | 2,18 | unknown |
| HORVU2Hr1G011010 | -2,95 | 4,94 | unknown |
| HORVU3Hr1G000770 | -2,78 | 2,07 | unknown |
| HORVU3Hr1G014850 | -2,60 | 2,76 | unknown |
| HORVU7Hr1G045510 | -2,59 | 3,72 | unknown |
| HORVU5Hr1G053840 | -2,47 | 2,27 | unknown |
| HORVU6Hr1G089750 | -2,46 | 9,80 | unknown |
| HORVU7Hr1G000970 | -2,44 | 4,63 | unknown |
| HORVU5Hr1G050470 | -2,42 | 7,74 | unknown |
| HORVU1Hr1G095210 | -2,42 | 2,10 | unknown |
| HORVU4Hr1G059200 | -2,23 | 3,22 | unknown |
| HORVU2Hr1G005510 | -2,19 | 3,16 | unknown |
| HORVU3Hr1G052250 | -2,19 | 4,14 | unknown |
| HORVU0Hr1G021760 | -2,19 | 2,25 | unknown |
| HORVU2Hr1G104300 | -2,17 | 2,44 | unknown |
| HORVU1Hr1G043710 | -2,16 | 2,32 | unknown |
| HORVU3Hr1G089620 | -2,12 | 4,23 | unknown |
| HORVU7Hr1G006520 | -2,08 | 9,45 | unknown |
| HORVU1Hr1G026590 | -2,08 | 2,44 | unknown |
| HORVU6Hr1G071950 | -2,06 | 3,38 | unknown |
| HORVU4Hr1G000770 | -2,03 | 2,31 | unknown |
| HORVU6Hr1G018060 | -2,16 | 2,47 | wee1-like protein kinase [EC:2.7.11.1] |
| HORVU2Hr1G036210 | -2,01 | 2,19 | WNK lysine deficient protein kinase [EC:2.7.11.1] |
| HORVU5Hr1G042000 | -3,19 | 8,02 | xyloglucan 6-xylosyltransferase [EC:2.4.2.39] |
| HORVU4Hr1G072940 | -2,11 | 2,70 | YTH (YT521-B homology) domain-containing |
| HORVU7Hr1G024220 | -2,24 | 10,43 | zinc finger five domain containing protein |
